# Publisher Correction: Immune correlates analysis of the PREVENT-19 COVID-19 vaccine efficacy clinical trial

**DOI:** 10.1038/s41467-023-37367-2

**Published:** 2023-03-22

**Authors:** Youyi Fong, Yunda Huang, David Benkeser, Lindsay N. Carpp, Germán Áñez, Wayne Woo, Alice McGarry, Lisa M. Dunkle, Iksung Cho, Christopher R. Houchens, Karen Martins, Lakshmi Jayashankar, Flora Castellino, Christos J. Petropoulos, Andrew Leith, Deanne Haugaard, Bill Webb, Yiwen Lu, Chenchen Yu, Bhavesh Borate, Lars W. P. van der Laan, Nima S. Hejazi, April K. Randhawa, Michele P. Andrasik, James G. Kublin, Julia Hutter, Maryam Keshtkar-Jahromi, Tatiana H. Beresnev, Lawrence Corey, Kathleen M. Neuzil, Dean Follmann, Julie A. Ake, Cynthia L. Gay, Karen L. Kotloff, Richard A. Koup, Ruben O. Donis, Peter B. Gilbert, Immune Assays Team, Coronavirus Vaccine Prevention Network (CoVPN)/2019nCoV-301 Principal Investigators and Study Team, United States Government (USG)/CoVPN Biostatistics Team

**Affiliations:** 1 Vaccine and Infectious Disease Division, Fred Hutchinson Cancer Center, Seattle, WA USA; 2 Public Health Sciences Division, Fred Hutchinson Cancer Center, Seattle, WA USA; 3 Department of Global Health, University of Washington, Seattle, WA 98195 USA; 4 Department of Biostatistics and Bioinformatics, Rollins School of Public Health, Emory University, Atlanta, GA USA; 5 Novavax, Inc., Gaithersburg, MD USA; 6 Biomedical Advanced Research and Development Authority, Washington, DC USA; 7 LabCorp-Monogram Biosciences, South San Francisco, CA USA; 8 Nexelis, Seattle, WA USA; 9 Department of Statistics, University of Washington, Seattle, WA USA; 10 Department of Biostatistics, T.H. Chan School of Public Health, Harvard University, Boston, MA USA; 11 Division of Microbiology and Infectious Diseases, National Institute of Allergy and Infectious Diseases, NIH, Rockville, MD USA; 12 Department of Laboratory Medicine and Pathology, University of Washington, Seattle, WA USA; 13 Center for Vaccine Development and Global Health, University of Maryland School of Medicine, Baltimore, MD USA; 14 Biostatistics Research Branch, National Institute of Allergy and Infectious Diseases, National Institutes of Health, Bethesda, MD USA; 15 U.S. Military HIV Research Program, Walter Reed Army Institute of Research, Silver Spring, MD USA; 16 University of North Carolina School of Medicine, Chapel Hill, NC USA; 17 Vaccine Research Center, National Institute of Allergy and Infectious Diseases, National Institutes of Health, Bethesda, MD USA; 18 Department of Biostatistics, School of Public Health, University of Washington, Seattle, WA USA

**Keywords:** Vaccines, Statistics, Adaptive immunity, Infectious diseases

Correction to: *Nature Communications* 10.1038/s41467-022-35768-3, published online 19 January 2023

Supplementary Information file was supposed to contain the Statistical Analysis Plan; this was not uploaded to ejp so was not included in the Supplementary Information file. Updated file now contains all figures/tables from the Supplementary Information file, plus the Statistical Analysis Plan, corresponding to the manuscript.

## Supplementary information

Updated Supplementary Information

