# Supplementary material for: Immune correlates analysis of the PREVENT-19 COVID-19 vaccine efficacy clinical trial
Source: Nat Commun. 2023 Jan 19;14:331. doi: 10.1038/s41467-022-35768-3 (PMC9851580; doi:10.1038/s41467-022-35768-3)
Supplement: Supplementary file 1 — Supplementary Material [file 41467_2022_35768_MOESM1_ESM.pdf]

Supplementary Material for "Immune Correlates Analysis of the PREVENT-19 COVID-19 Vaccine Efficacy Clinical Trial" by Fong et al.

Immune Assays Team

Affiliation	Team Members
Biomedical Advanced Research and Development Authority (BARDA), Washington, DC	Oleg Borisov, Flora Castellino, Brett Chromy, Mark Delvecchio, Ruben O. Donis, Tremel Faison, Corey Hoffman, Christopher Houchens, Tom Hu, Pennie Hylton, Lakshmi Jayashankar, Aparna Kolhekar, James Little, Karen Martins, Jeanne Novak, Azhar Ravji, Carol Sabourin, Evan Sturtevant, Kimberly Taylor, Xiaomi Tong, John Treanor, Danielle Turley, Leah Watson, Daniel Wolfe
Boston Consulting Group, Boston, MA	Gian King, Andrew Li, Najaf Shah, Smruthi Suryaprakash, Jue Xiang Wang
Division of AIDS, NIAID, NIH, Bethesda, MD	Patricia D'Souza
Division of MID (Microbiology and Infectious Diseases), NIAID, NIH, Bethesda, MD	Janie Russell
Duke University, Durham, NC	David Beaumont, Kendall Bradley, Jiayu Chen, Xiaoju Daniell, Thomas Denny, Elizabeth Domin, Amanda Eaton, Kelsey Engel, Wenhong Feng, Juanfei Gao, Hongmei Gao, Kelli Greene, Sarah Hiles, Leihua Liu, Kristy Long, Kellen Lund, Charlene McDanal, David C. Montefiori, Marcella Sarzotti-Kelsoe, Francesca Suman, Haili Tang, Jin Tong, Olivia Widman
LabCorp-Monogram Biosciences, South San Francisco, CA, USA	Christos J. Petropoulos, Terri Wrin
The Tauri Group, an LMI company - Contract Support for U.S. Department of Defense (DOD) Joint Program Executive Office for Chemical, Biological, Radiological and Nuclear Defense (JPEO-CBRND) Joint Project Manager for Chemical, Biological, Radiological, and Nuclear Medical (JPM CBRN Medical), Fort Detrick, Maryland, USA	Christopher S. Badorrek, Gregory E. Rutkowski
Vaccine Research Center, NIAID, NIH, Bethesda, MD	Obrimpong Amoa-Awua, Manjula Basappa, Robin Carroll, Britta Flach, Suprabhath Gajjala, Nazaire Jean-Baptiste, Richard A. Koup, Bob C. Lin, Adrian McDermott, Christopher Moore, Mursal Naisan, Muhammed Naqvi, Sandeep Narpala, Sarah O'Connell, Clare Whittaker, Weiwei Wu, Allen Mueller, Martin Apgar, Tommy Bruington, Joe Stashick, Leo Serebryannyy, Mike Castro, Jennifer Wang

2019nCoV-301 Study Group (Pubmed listed, in alphabetical order of institution affiliation)

Affiliation/Funding*	Study Group	Location
**México**		
Centro de Atención e Investigación Médica (CAIMED)	Jorge F. Méndez Galván, MD, Monica B. Carrascal, Adriana Sordo Duran, Laura Ruy Sanchez Guerrero, Martha Cecilia Gómora Madrid	Mexico City, Mexico
FAICIC Clinical Research	Alejandro Quintín Barrat Hernández, MD, Sharzhaad Molina Guizar, Denisse Alejandra González Estrada, Silvano Omar Martínez Pérez, MD, Zindy Yazmín Zárate Hinojosa, MD	Veracruz, Mexico
Instituto Nacional de Ciencias Médicas y Nutrición Salvador Zubirán	Guillermo Miguel Ruiz-Palacios, MD	Mexico City, Mexico
Instituto Nacional de Salud Pública	Aurelio Cruz-Valdez, PhD, Janeth, Pacheco-Flores, MD, Anyela Lara, MD, Secia Díaz-Miralrio	Cuernavaca, Mexico
PanAmerican Clinical Research México	María José Reyes Fentanes, MD, Jocelyn Zuleica Olmos Vega, MD, Daniela Pineda Méndez, MD, Karina Cano Martínez, MD, Winniberg Stephany Alvarez León	Querétaro, Mexico
PanAmerican Clinical Research México	Vida Veronica Ruiz Herrera, MD, Eduardo Gabriel Vázquez Saldaña, Laura Julia Camacho Choza, Karen Sofia Vega Orozco, Sandra Janeth Ortega Domínguez	Guadalajara, Mexico
Unidad de Atención Médica e Investigación en Salud (UNAMIS)	Jorge A. Chacón, MD, Juan J. Rivera, MD, Erika A. Cutz, MD, Maricruz E. Ortegón, MD, María I. Rivera, MD	Mérida, Mexico
**United States and Puerto Rico**		
Accellacare	David Browder, MD, Cortney Burch, Terri Moye, Paul Bondy, MD, Lesley Browder, MD	Rocky Mount, NC
Accellacare	Rickey D. Manning, MD, James Wilson Hurst, MD, Rodney E. Sturgeon, MD, Paul H. Wakefield, MD, John A. Kirby, MD	Knoxville, TN
Accel Research Sites	James Andersen, MD, Szheckera Fearon, MSN, FNP-C, Rosa Negron, MD, Amy Medina, ADN, BS	Lakeland, FL
Accel Research Sites	Bruce Rankin, DO, John M. Hill, MD, Steven Shinn, MD, Vivek Rajasekhar, DO, Marshall Nash, MD	DeLand, FL
Achieve Clinical Research	Hayes Williams, MD, PhD, LaShondra Cade, Rhodna Fouts, Connie Moya	Birmingham, AL
Alliance for Multispecialty Research	Corey G. Anderson, MD, Naomi Devine, NP-C, James Ramsey, NP-C, Ashley Perez, David Tatelbaum	Tempe, AZ
Alliance for Multispecialty Research	Michael Jacobs, MD, Kathleen Menasche, LPN, Vincent Mirkil, MD	Las Vegas, NV
Anaheim Clinical Trials	Peter J. Winkle, MD, Amina Z. Haggag, MD, Michelle Haynes, Marysol Villegas, Sabina Raja	Anaheim, CA
Atlanta Center for Medical Research	Robert Riesenberg, MD, Stanford Plavin, MD, Mark Lerman, MD, Leana Woodside, DNP, NP-C, Maria Johnson, MD	Atlanta, GA
Baylor College of Medicine / NIAID (UM1AI148575)	C. Mary Healy, MD, Jennifer A. Whitaker, MD, Hana El Sahly, MD, Christine Akamine, MD, Wendy A. Keitel, MD, Robert L. Atmar, MD	Houston, TX
Biomedical Advanced Research and Development Authority (BARDA)	Richard Gorman, MD, Gary Horwith, MD, Robin Mason, MS, MBA	Washington, DC
Benchmark Research	Laurence Chu, MD, Michelle Chouteau, MD, Lisa Johnson, FNP, Tambra Dora	Austin, TX
Benchmark Research	Greg Hachigian, MD, Deborah Murray, FNP, Michael Cancilla, PA, Logan Ledbetter, PA, Masaru Oshita, MD	Sacramento, CA
Benchmark Research	William Seger, MD, Beverly Ewing, APRN, DNP, FNP-BC	Fort Worth, TX
Beth Israel Deaconess Medical Center / NIAID (UM1AI068614)	Kathryn E. Stephenson, MD, MPH, Chen Sabrina Tan, MD, Rebecca Zash, MD, Jessica L. Ansel, MSN, Kate Jaegle, MSN, Caitlin J. Guiney, MSN	Boston, MA
Black Hills Center for American Indian Health / Missouri Breaks Industries Research Inc / NIAID (UM1AI068614)	Jeffrey A. Henderson, MD, MPH, Marcia O'Leary, RN, Kendra Enright, RN, Jill Kessler, MS, Pete Ducheneaux, LPN, Asha Inniss, MS, APRN	Eagle Butte, SD
California Research Foundation	Donald M. Brandon, MD, William B. Davis, MD, Daniel T. Lawler, MD	San Diego, CA
Carolina Institute for Clinical Research	Yaa D. Oppong, MD, Ryan P. Starr, DO, Scott N. Syndergaard, DO, Rozeli Shelly, MD, Mashrur Islam Majumder	Fayetteville, NC
Cedar Crosse Research Center	Danny Sugimoto, MD, Jeffrey Dugas Sr., MD, Dolores Rijos, Sandra Shelton, Stephan Hong, MD	Chicago, IL
Cenexel RCA	Howard Schwartz, MD, Nelia Sanchez-Crespo, MD, Jennifer Schwartz, APRN, Terry Piedra, BS, Barbara Corral, APRN	Hollywood, FL
Centex Studies	Joel Solis, MD, Carmen Medina, PA, Westley Keating, PA	McAllen, TX
Clinical Neuroscience Solutions	Michael E. Dever, MD, Mitul Shah, MD, Michael Delgado, MD, Tameika Scott, DrPH	Orlando, FL
Clinical Neuroscience Solutions	Lisa S. Usdan, MD, Lora J. McGill, MD, Valerie K. Arnold, MD, Carolyn Scatamacchia, MSN, NP-C, Codi M. Anthony, DNP, APRN, PMHNP-BC	Memphis, TN
CommonSpirit Health Research Institute	Rajan Merchant, MD, Anelgine Crans Yoon, MD, Janet Hill, PA-C, Lucy Ng-Price, MA, Teri Thompson-Seim	Woodland, CA
Comprehensive Clinical Research	Ronald Ackerman, MD, Jamie Ackerman, Florida Aristy, APRN	West Palm Beach, FL
Covid-19 Prevention Network (CoVPN)	Lawrence Corey, MD, Kathleen M Neuzil, MD, MPH, Huub G Gelderblom, MD, PhD, Nzeera Ketter, Carrie Sopher	Seattle, WA
CRA Headlands	Jon Finley, MD, Nathan Segall, MD, Mildred Stull, APRN, FNP-C	Stockbridge, GA
DM Clinical Research	Vicki E. Miller, MD, MPH, Monica Murray, Blanca Gomez, Zainab Rizvi, Sonia Guerrero	Tomball, TX
Empire Clinical Research	Yogesh K. Paliwal, MD, Amit Paliwal, MD, Sarah Gordon, MS, Bryan Gordon, Cynthia Montano-Pereira	Pomona, CA
Headlands Research	Christopher Galloway, MD, Candice Montros, Lily Aleman, Samira Shairi, RN, Wesley Van Ever	Orlando, FL
Health Research of Hampton Roads	George H. Freeman, MD, Esther Laverne Harmon, ANP, Marshall A. Cross, MD, Kacie Sales, BSN, RN, Catherine Q. Gular, PharmD	Newport News, VA
HHS-DoD Countermeasures Acceleration Group	Matthew Hepburn, MD	Washington, DC

HOPE Research Institute	Matthew Doust, MD, Nathan Alderson, PhD, Shana Harshell	Phoenix, AZ
Howard University Hospital / Howard University College of Medicine / NIAID (UM1AI068614)	Siham Mahgoub, MD, Celia Maxwell, MD, Thomas Mellman, MD, Karl M Thompson, PhD, Glenn Wortman, MD	Washington, DC
IACT Health	Jeff Kingsley, DO, April Pixler, LaKondria Curry, Sarah Afework, Austin Swanson	Columbus, GA
Jacksonville Center for Clinical Research	Jeffry Jacqmein, MD, Maggie Bowers, PA-C, Dawn Robison, APRN-C, Victoria Mosteller, MD, Janet Garvey, DNP	Jacksonville, FL
Johnson County Clin-Trials	Carlos Fierro, MD, Mary Easley, BSN, RN	Lenexa, KS
Joint Program Executive Office for Chemical, Biological, Radiological and Nuclear Defense's, US Department of Defense	Rebecca J. Kurnat	Washington, DC
Lynn Health Science Institute	Carl P. Griffin, MD, Raymond Cornelison, MD, Shanda Gower, APRN, CNP, William Schnitz, MD, Destiny S. Heinzig-Cartwright, BA	Oklahoma City, OK
Lynn Institute of the Ozarks	Derek Lewis, MD, Fred E. Newton, MD, Aeiress Duhart, Breana Watkins, Brandy Ball	Little Rock, AR
Lynn Institute of the Rockies	Ripley Hollister, MD, Jeremy Brown, DO, Melody Ronk, PA-C, Jill York, Shelby Pickle	Colorado Springs, CO
M3-Emerging Medical Research	David B. Musante, MD, William P. Silver, MD, Linda R. Belhorn, MD, Nicholas A. Viens, MD, David Dellaero, MD	Durham, NC
M3-Wake Research	Matthew Hong, MD, Wayne Harper, MD, Lisa Cohen, DO, Priti Patel, NP, Kendra Lisec, PA	Raleigh, NC
MD Clinical	Beth Safirstein, MD, Luz Zapata, MD, Lazaro Gonzalez, APRN, Evelyn Quevedo, APRN, Farah Irani, PhD	Hallandale Beach, FL
Medical Research International	Joseph Grillo, MD, Amy Potts, PA-C, MPH, Julie White, MBA	Oklahoma City, OK
Medical University of South Carolina	Patrick Flume, MD, Gary Headden, MD, Brandie Taylor, NP, Ashley Warden, Amy Chamberlain	Charleston, SC
MedPharmics	Robert Jeanfreau MD, Susan Jeanfreau MD	Metairie, LA
MedPharmics	Paul G. Matherne, MD, Amy Caldwell, RN, Jessica Stahl, Mandy Vowell, Lauren Newhouse	Gulfport, MS
Meharry Medical College / NIAID (UM1AI068614)	Vladimir Berthaud MD, MPH, Zudi-Mwak Takizala MD, MPH, MBA, Genevieve Beninati, FNP, Kimberly Snell, PharmD, Sherrie Baker, BS, James Walker, RN	Nashville, TN
Meridian Clinical Research	David Ensz, MD, Tavane Harrison, CNP, Meagan Miller, Janet Otto	Sioux City, IA
Meridian Clinical Research	Brandon Essink, MD, Roni Gray, APRN, Christine Wilson, Tiffany Nemecek, Hannah Harrington, MPH	Omaha, NE
Meridian Clinical Research	Charles Harper, MD, Keith Vrbicky, MD, Chelsie Nutsch, NP, Sally Eppenbach, NP, Wendell Lewis, NP	Norfolk, NE
Meridian Clinical Research	Jordan Whatley, MD, Christopher Dedon, APRN, FNP-C, Tana Bourgeois, RN, Lyndsea Folsom, Crystal Rowell, APRN, FNP-C	Baton Rouge, LA
Miami Veterans Affairs Medical Center / NIAID (UM1AI068614)	Gregory Holt, MD, Mehdi Mirsaeidi, MD, Rafael Calderon, MD, Paola Lichtenberger, MD, Jalima Quintero, RN, Becky Martinez, RN	Miami, FL
Morehouse School of Medicine / NIAID (UM1AI068614)	Lilly Immergluck, MD, Erica Johnson, PhD, Austin Chan, MD, Norberto Fas, MD, LaTeshia Thomas-Seaton, MS, APRN, Saadia Khizer, MD, MPH	Atlanta, GA
MultiCare Institute for Research and Innovation	Jonathan Staben, MD	Cheney, WA
National Institute of Allergy and Infectious Diseases (NIAID) / National Institutes of Health (NIH)	Tatiana Beresnev, MD, Maryam Jahromi, MD, Mary A. Marovich, MD, Julia Hutter, MD, Martha Nason, PhD, Julie Ledgerwood, DO, John Mascola, MD	Bethesda, MD
National Research Institute	Mark Leibowitz, MD, Fernanda Morales, Mike Delgado, Rosario Sanchez, Norma Vega	Los Angeles, CA
Novavax, Inc.	Lisa M. Dunkle, MD, Germán Áñez, MD, Gary Albert, Erin Coston, Chinar Desai, Haoua Dunbar, Mark Eickhoff, Jenina Garcia, Margaret Kautz, Angela Lee, Maggie Lewis, Alice McGarry, Irene McKnight, Joy Nelson, Patrick Newingham, Patty Price-Abbott, Patty Reed, Diana Vegas, Bethanie Wilkinson, PhD, Katherine Smith, MD, Wayne Woo, MS, Iksung Cho, MS, Gregory M. Glenn, MD, Filip Dubovsky, MD, MPH	Gaithersburg, MD
Omega Medical Research	David L. Fried, MD, Lynne A. Haughey, MSN, FNP, Ariana C. Stanton, PA-C, Lisa Stevens Rameaka, MD	Warwick, RI
Pharmacology Research Institute	David Rosenberg, MD, Lee Tomatsu, Viviana Gonzalez, Millie Manalo	Los Alamitos, CA
PMG Research of Bristol	Bernard Grunstra, MD, Donald Quinn, MD, Phillip Claybrook, MD, Shelby Olds, MD, Amy Dye	Bristol, TN
PMG Research of Wilmington	Kevin D. Cannon, MD, Mesha M. Chadwick, MD, Bailey Jordan, Morgan Hussey, Hannah Nevarez	Wilmington, NC
Ponce de Leon Center / NIAID (UM1AI068614)	Colleen F. Kelley, MD, MPH, Valeria D. Cantos MD, Michael Chung MD, Caitlin Moran, MD, MSc, Paulina Rebolledo, MD, Christina Bacher, PAC	Atlanta, GA
Ponce School of Medicine / NIAID (UM1AI148685)	Elizabeth Barranco-Santana, MD, Jessica Rodriguez, MD, Rafael Mendoza, MD, Karen Ruperto, MD, Odette Olivieri, MD, Enrique Ocaña, MD	Ponce, Puerto Rico
Preferred Research Partners	Paul E. Wylie, MD, Renea Henderson, DO, Natasa Jenson, MD, Fan Yang, MD, Amy Kelley, BSN, RN	Little Rock, AR
Providea Health Partners Elligo Health Research	Kenneth Finkelstein, DO, David Beckmann, MD, Tanya Hutchins, FNP, Sebastian Garcia Escallon, BA, Kristen Johnson	Evergreen Park, IL
Providence Clinical Research	Teresa S. Sligh, MD, Parul Desai, NP, Vincent Huynh, BSc, Carlos Lopez, MD, Erika Mendoza, BA	North Hollywood, CA
Research Your Health	Jeffrey Adelglass, MD, Jerome (Jerry) G. Naifeh, MD, Kristine Jane Kucera, PA-C, MPAS, DHS, Waseem Chughtai, BS, MBBS, Shireen Hasham Jaffer	Plano, TX
Rochester Clinical Research	Matthew G. Davis, MD, Jennifer Foley, Michelle Lyn Burgett, RN, Tammi Louise Shlotzhauer, MD, Sarah Michelle Ingalsbe-Geno, RPA-C	Rochester, NY
SIMEDHealth / SIMEDResearch	Daniel Duncanson, MD, Kelly Kush, Lori Nesbitt, Cora Sonnier, Jennifer McCarter	Gainesville, FL

Sterling Research Group	Michael B. Butcher, MD, James Fry, PA-C, Donna Percy, RN, BSN, Karen Freudemann	Cincinnati, OH
Sterling Research Group	Bruce C. Gebhardt, MD, Padma N. Mangu, MD, Debra Beck Schroeck, MS, PA-C, Rajesh Kumar Davit, MD, Gayle D. Hennekes, PA-C, MPAS	Cincinnati, OH
Stony Brook University - Stony Brook Medicine / NIAID (UM1AI068614)	Benjamin J. Luft, MD, Melissa Carr, BA, Sharon Nachman, MD, Alison Pellecchia, BA, Candace Smith, PharmD, Bruno Valenti, NP	Commack, NY
Suncoast Research Associates	Maria I. Bermudez, MD, Noris Peraita, ARNP, Ernesto Delgado, ARNP, Alicia Arrazcaeta, Natalie Ramirez	Miami, FL
Suncoast Research Group	Mark E. Kutner, MD, Jorge Caso, MD, Janet Mendez, ARNP, Marianela Carvajal, ARNP, Carmen Amador, ARNP	Miami, FL
Sundance Clinical Research	Larkin Tyler Wadsworth III, MD, Horacio Marafioti, MD, Lyly Dang, DNP-BC, Lauren Clement, NP-C, Jennifer Berry, FNP-BC	St. Louis, MO
Synexus Clinical Research	Mohammed Allaw, MD, Georgettea Geuss, Chelsea Miles, NP, Zachary Bittner, Melody Werne	Evansville, IN
Synexus Clinical Research	Cornell Calinescu, MD, Shannon Rodman, Joshua Rindt	Henderson, NV
Synexus Clinical Research	Erin Cooksey, MD, Kristina Harrison, Deanna Cooper, Manisha Horton Amanda Philyaw	Anderson, SC
Synexus Clinical Research	William Jennings, MD, Hilario Alvarado, MD, Michele Baka, MD, Malina Regalado, NP	San Antonio, TX
Synexus Clinical Research	Linda Murray, DO	Pinellas Park, FL
Synexus Clinical Research	Sherif Naguib, MD, Justin Singletary, Sha-Wanda Richmond, Sarah Omodele, Emily Oppenheim	Atlanta, GA
Synexus Clinical Research	Joseph Newberg, MD, Laura Pearlman, MD, Reuben Martinez, Victoria Andriulis	Chicago, IL
Synexus Clinical Research	Paul J. Nugent, DO, Leonard Singer, MD, Jeanne Blevins, Meagan Thomas, Christine Hull	Cincinnati, OH
Synexus Clinical Research	Isabel Pereira, MD, Gina Rivero, Tracy Okonya, Frances Downing, Paulina Miller	Vista, CA
Synexus Clinical Research	Margaret Rhee, MD, Katherine Stapleton, Jeffrey Klein, Rosamond Hong, MD	Akron, OH
Synexus Clinical Research	Suzanne Swan, MD, Tami Wahlin, MD, Elizabeth Bennett, PA, Amy Salzl Sharine Phan	Richfield, MN
Synexus Clinical Research	Jewel Johnny White, MD, Amanda Occhino, Ruth Paiano APRN, Morgan McLaughlin APRN, Elisa Swieboda APRN	The Villages, FL
Texas Center for Drug Development	Veronica Garcia-Fragoso, MD, Maria Gabriela Becerra, MD, Cecilia Mckeown, Lisa Holloway, Toni White	Houston, TX
The Charlotte-Mecklenburg Hospital Authority d/b/a Atrium Health / NIAID (UM1AI068614)	Christine B. Turley, MD, Andrew McWilliams, MD, Tiffany Esinhart, PA-C, Natasha Montoya, APRN, Shamika Huskey, FNP, Leena Paul, FNP	Charlotte, NC
The Miriam Hospital / NIAID (UM1AI068636)	Karen Tashima, MD, Jennie Johnson, MD, Marguerite Neill, MD, Martha Sanchez, MD, Natasha Rybak, MD, Maria Mileno, MD	Providence, RI
UC Davis Health / NIAID (UM1AI068614)	Stuart H. Cohen, MD, Monica Ruiz, Dean M. Boswell, BS, Elizabeth E. Robison, BS, Trina L. Reynolds, BS, Sonja Neumeister, MPH	Sacramento, CA
Universidad de Puerto Rico - Recinto de Ciencias Médicas - Maternal Infant Studies Center (CEMI) / NIAID (UM1AI068636)	Carmen D. Zorrilla, MD, Juana Rivera, MD, MPH, Jessica Ibarra, MD, Iris García, BSN, RN, Dianca Sierra, BA, Wanda Ramon, BSPh	San Juan, Puerto Rico
University of Colorado Hospital CRS / NIAID (UM1AI068636) / NCATS (UL1TR002535, UM1AI069432)	Thomas B. Campbell, MD, Suzanne Fiorillo, MSPH, Rebecca Pitotti, RNP, Victoria Riedel Anderson, MS, Jose Castillo Mancilla, MD, Nga Le, PharmD	Aurora, CO
University of Iowa Medical Center / NIAID (UM1AI068614) / NCATS (UL1TR002537)	Patricia L. Winokur, MD, Dilek Ince, MD, Theresa Hegmann, PA, Jeffrey Meier, MD, Jack Stapleton, MD, Laura Stulken, PA	Iowa City, IA
University of Maryland School of Medicine / NIAID (UM1AI148689)	Monica McArthur, MD, PhD, Karen L. Kotloff, MD, Kathleen Neuzil, MD, Andrea Berry, MD, Milagritos Tapia, MD, Elizabeth Hammershaimb, MD, MS, Toni Robinson, RN, Rosa MacBryde, RN	Baltimore, MD
University of Minnesota / NIAID (UM1AI068614)	Susan Kline, MD, MPH, Joanne L. Billings, MD, MPH, Winston Cavert, MD, Les B. Forgosh. MD, Timothy W. Schacker, MD, Tyler D. Bold, MD, PhD	Minneapolis, MN
University of Missouri Health Care / NIAID (UM1AI148685)	Dima Dandachi, MD, MPH, Taylor Nelson, DO, Andres Bran, MD, Grant Geiger, S. Hasan Naqvi, MD	Columbia, MO
University of Nebraska Medical Center / NIAID (UM1AI068614)	Diana F Florescu, MD, Richard Starlin, MD, David Kline, MD, Andrea Zimmer, MD, Anum Abbas, MD, Natasha Wilson, APRN	Omaha, NE
University of North Carolina / NIAID (UM1AI068619) / University of North Carolina at Chapel Hill Center for AIDS Research (P30AI050410) / NC TraCS Institute (UL1TR002489)	Cynthia L. Gay, MD, MPH, Joseph J Eron, MD, Michael Sciaudone, MD, MPH, A. Lina Rosengren, MD, MPH, MS, John S Kizer, MD, Sarah E Rutstein, MD, PhD	Chapel Hill, NC
University of South Florida, Morsani College of Medine / NIAID (UM1AI068614)	Carina A. Rodriguez, MD, Elizabeth Bruce, MD, Claudia Espinosa, MD, Lisa J Sanders, MD, Kami Kim, MD, Denise Casey, RN	Tampa, FL
University of Texas Health Science Center San Antonio / NIAID (UM1AI068614)	Barbara S. Taylor, MD, MS, Thomas Patterson, MD, Ruth Serrano Pinilla, MD, Delia Bullock, MD, Philip Ponce, MD, Jan Patterson, MD	San Antonio, TX
University of Washington / Lummi Tribal Health Center / NIAID (UM1AI148573)	R. Scott McClelland, MD, MPH, Dakotah C. Lane, MD, Anna Wald, MD, MPH, Frank James, MD, Elizabeth Duke, MD, Kirsten Hauge, MPH, Jessica Heimonen, MPH	Seattle, WA
University of Washington	Robert W. Coombs, MD, PhD, Alex Greninger, MD, PhD, MS, MPhil, Pavitra Roychoudhury, PhD, Erin A. Goecker, MS, Yunda Huang, PhD, Youyi Fong, PhD	Seattle, WA
VA Ann Arbor Healthcare System / NIAID (UM1AI068614)	Carol Kauffman, MD, Kathleen Linder, MD, Kimberly Nofz, BSN, Andrew McConnell, BS	Ann Arbor, MI

Velocity Clinical Research	Robert J. Buynak, MD, Angella Webb, APRN, Taryn Petty, FNP, Stephanie Andree, FNP	Valparaiso, IN
Velocity Clinical Research	Judith Kirstein, MD, Marcia Bernard, Erica Sanchez, Nolan Mackey, Clarisse Baudelaire	Banning, CA
Velocity Clinical Research	Gregg Lucksinger, MD, Jaleh Ostovar, NP	Medford, OR
Velocity Clinical Research	Mary Beth Manning, MD, Joan Rothenberg, MD, Toby Briskin, MD, Denise Roadman, PAC, Sarah Dzigiel	Cleveland, OH
Velocity Clinical Research	J. Scott Overcash, MD, Adrienna Marquez, Hanh Chu, Kia Lee, Kim Quillin	La Mesa, CA
Velocity Clinical Research	Barbara Rizzardi, MD, Michelle King, NP, Vanessa Abad, NP, Jennifer Knowles, BS	West Jordan, UT
Velocity Clinical Research	Michael Waters, MD, Karla Zepeda, NP, Scott Overcash, MD, Jordan Coslet, NP, Dalia Tovar, MA	Chula Vista, CA
Velocity Clinical Research	Marian E. Shaw, MD, Mark A. Turner, MD, Cory J. Huffine, FNP-C, Esther S. Huffine, FNP-C	Meridian, ID
Walter Reed Army Institute of Research	Julie A. Ake, MD, MSc	Silver Spring, MD
Wayne State University / NIAID (UM1AI068614)	Elizabeth Secord, MD, Eric McGrath, MD, Phillip Levy, MD, Brittany Stewart, RD, PharmD, Charnell Cromer, RN, MSN, Ayanna Walters, RN, BSN	Detroit, MI
Weill Cornell Chelsea CRS / NIAID (UM1AI068619)	Kristen Marks, MS, MD, Grant Ellsworth, MD, MS, Caroline Greene, ANP-BC, Sarah Galloway, BA, Shashi Kapadia, MD, MS, Elliot DeHaan, MD	New York, NY
Willis-Knighton Health System / WKB Family Medicine Associates	Clint Wilson, MD, Jason Milligan, MD, Danielle Raley, MD, Joseph Bocchini, MD	Bossier City, LA
Womack Army Medical Center	Bruce McClenathan, MD, Mary Hussain, BS, Evelyn Lomasney, MD, Evelyn Hall, MMS, PA-C, Sherry Lamberth, PharmD	Fort Bragg, NC
WR Clinsearch	Mark McKenzie, MD, Teresa Deese, Christy Schmeck, Vickie Leathers, Christy Sweet	Chattanooga, TN

* Funding of institutions by the National Institute of Allergy and Infectious Diseases (NIAID) and/or research support by the National Center for Advancing Translational Science (NCATS), as indicated. All other institutions were funded by Office of the Assistant Secretary for Preparedness and Response, Biomedical Advanced Research and Development Authority. The content of this publication is solely the responsibility of the authors and does not necessarily represent the official views of the funding sources.

2019nCoV-301 Principal Investigators and Study Team (in alphabetical order)

Principal Investigator	Study Team	Institution	Location
Ronald Ackerman, MD	Jamie Ackerman, Florida Aristy, Tomeko Heard, Diana Mann, Maureen Stewart, Cheryl Demczyk, Rohan Barron, Ashley Torres, Jennifer Gomez, Tiffany Potter	Comprehensive Clinical Research	West Palm Beach, FL
Jeffrey Adelglass, MD	Jerome (Jerry) G. Naifeh, Kristine Jane Kucera, Waseem Chughtai, Shireen Hasham Jaffer, Anuja Sathe, Cameron Galownia, Cheryl Hill, Ramiro Lopez, Erica Parker-Martinez, Helene Harrison, Chiedza Mutindori, Sabrina Flowers, Tamara Betters, Carolyn Ackley, Pamela Fox, Noelia Tejada James, Dorothy Saylor, Hallen Dao, Jon Etta Randolph, Jason Tentativa, Malaika Chughtai, Shanzae Chughtai, Maheen Shah, Hayyan Chughtai, Tyler Love, Ti'arah Love	Research Your Health	Plano, TX
Mohammed Allaw, MD	Georgettea Geuss, Chelsea Miles, Zachary Bittner, Melody Werne, Lyndsey Morrison, Stephanie Albin, Linda Frazier, Jacque Nalley, Christie Borin, Jacque Nalley	Synexus Clinical Research	Evansville, IN
James Andersen, MD	Szheckera Fearon, Rosa Negron, Amy Medina, Diana Holmes, Colleen Figueroa, Cristal Ruiz, Nancy Masseus Tare Floyd, Kenta Oliver, Candice Gerber, Mae Ann Francisco, Gilbert de la Cruz, Ginny McClanahan, Veronica Walker, David Irwin, Gloria Adejobi	Accel Research Sites	Lakeland, FL
Corey G. Anderson, MD	Naomi Devine, James Ramsey, Tyanna Montijo, Ashley Perez, David Tatelbaum, Lisa M. Dean, Angela D. Ledezma, Anthony Padilla, Cecilia M. Tanori, Georgina Lopez-Wood, Tasha C. Marriott, Ronald Hawkins, Hannah Spinks	Alliance for Multispecialty Research	Tempe, AZ
Elizabeth Barranco-Santana, MD	Michele Irizarry, Alice Grace Rodriguez, Irmaris Arroyo, Sara Cancel, Alejandra Román, Juan D. Lugo, Armando X. Torres, Marianne Hernandez, Brenda Garcia, Nancy Jiménez, Orlando Torres	Ponce School of Medicine	Ponce, Puerto Rico
Alejandro Quintín Barrat Hernández, MD	Sharzhaad Molina Guizar, Denisse Alejandra González Estrada, Silvano Omar Martínez Pérez, Zindy Yazmín Zárate Hinojosa, Norberto Daniel Vázquez Tinajero, Yessica Olivo Domínguez, Daniel Hernández León, Gloria Norma Ambrosio Lara, José Carlos Mateos Castro, Irving Nerí Leyva Ferrer, María Fernanda Hernández García, Heidy Jazmín Maldonado Pavón, Evelyn Monserrat Bravo Serralta, Edgar Iván Muñoz López, Karina Esmeralda García Mateo, Lorena Cruz Cruz, José Javier Zárate Hinojosa, Javier Torres Cole, Yareth Jiménez Barcenas, Andrea Anaid Rangel Huerta, Erika Guillén González, María de la Luz Rufina Martínez Lugo, Angélica Liliana Muñoz Solano, David Sena Gómez, Berenice Valera Montalvo, Moisés Miguel Ruíz Nogueira, Yoshira Montero Díaz, Francisco Javier Martínez Osorio, Alejandra Morales Arias, Sandra Itzel Solis Rivera, Alejandro Esteban Cortina, Aldo Miguel López Domínguez, María Fernanda Cortés Ruíz, Marilyn Yulissa Ramírez Domínguez, Lucero Moctezuma Juan, Francisco Barrales Arcos	FAICIC Clinical Research	Veracruz, Mexico
Maria I. Bermudez, MD	Noris Peraita, Ernesto Delgado, Alicia Arrazcaeta, Natalie Ramirez, Giovanna Salcedo, Aliana Amador, Elizabeth Martinez, Arleen Aspuru, Gabriella Gonzalez, Gabriella Alabaci, Livan Sanchez, Raul Tejeda, Adriana Bello, Barbara Vega-Aguera, Kassandra Martinez, Grettel Obregon, Oscar Alejandro Gutierrez Luna, Magela C. Dominguez, Lauren Pena	Suncoast Research Associates	Miami, FL
Vladimir Berthaud, MD, MPH	Toni Hall, Livette Johnson, Sylvia Eluhu, Ana Tomescu, Katharina Whitbeck, Rajbir Singh	Meharry Medical College	Nashville, TN
Donald M. Brandon, MD	William B. Davis, Daniel T. Lawler, Maria Aceves, Kathleen B. Anderson, Hana Berry, Janice E. Brandon, Jeffrey C. Brandon, Patricia A. Brandon, Lorraine Boggs, Charlene Cruz, Mairead Hawkins, Clarice Hranicky, Andrew J. McCrea, Karen G. McCrea, Kimberly Najera, Tierney J. O'Connor, Michelle L. Rios, Cindy F. Stevens, Hannah J. Zapata	California Research Foundation	San Diego, CA
David Browder, MD	Cortney Burch, Terri Moye, Michael Wright, Paul Bondy, Lesley Browder	Accellacare	Rocky Mount, NC
Michael B. Butcher, MD	James Fry, Julia Froschauer, Allison Deuel, Jeanne Piccola, Donna Percy, Karen Freudemann, Lois Rawe, Megan Bryant, Kurt Percy, Jon Marvin, Luann Corcoran	Sterling Research Group	Cincinnati, OH
Robert J. Buynak, MD	Mark Yarosz, Rachel McNeal, Megan Smith, Patricia Volom, Nicholas Hanna, Erica Lewis, Miranda Lee, Goldie Luna, Marilyn Idowu, Destiny Williams, Jessica Johnson, Consuelita Perez, Priscilla Dodson	Velocity Clinical Research	Valparaiso, IN
Cornell Calinescu, MD	Shannon Rodman, Joshua Rindt, Krystal Tyner, Lovelyn Vincente, Alejandro Osuna-Meda, Charmaine Brown, Matthew Derrick, Melodee Morrison, Marissa Washington	Synexus	Henderson, NV
Thomas B. Campbell, MD	Donna McGregor, Laurel Ware, Myron Levin, Steven Johnson, Sophia Quesada, Martin Krsak, Kristine Erlandson, Nicholas Sarchet, Vanessa Sutton, Lawrence Moran, Tracey Stevenson, Alaina Dougherty, Julianne Randlemon	University of Colorado Hospital CRS	Aurora, CO
Kevin D. Cannon, MD	Mesha M. Chadwick, Bailey Jordan, Taylor Fedorcha, Kathryn Zweier, Brettany Holt, Emily Johnson, Karen Ruggiero, Olivia Houghton, Courtney Christie, Allison Dunn, Courtney Boyce, Sasha Saint-Lot, Ashley Andrades, Ashley Miller, LaShaya Dunston, Russell Larkins, Brittany Savoca, Hannah Nevarez, Hannah Nevarez, Larkin Collins, Morgan Cyrus, Morgan Hussey, Christina MacNaughton, Heidi Kaufman, Sheila Gard, Alyssa Gaylor, Bethany Donelan-Wilson, Taylor Bayless, Anna McManus, Tracie Marlowe Bryant, Ben Manuel, Laura McMillan, Nicole Stigers, Prerana Zanke	PMG Research of Wilmington	Wilmington, NC
Jorge A. Chacon, MD	Juan J. Rivera, Erika A. Cutz, Maricruz E. Ortegon, María I. Rivera, Ricardo Cervera, Felipe Rivera, Daniela Pat, Daniela Cruz, Alberto Chacon, Kattia Borges, Aldo Borraz, Rebeca Ortegon, Karla Ic, Carmen Ojeda, Irvin Ortega, Mayra Jimenez, Cindy Novelo, Pharmacist, Mónica Pérez, Adriana Hernandez, Laura Martinez	Unidad de Atención Medica e Investigación en Salud (UNAMIS)	Merida, Mexico
Laurence Chu, MD	Michelle Chouteau, Lisa Johnson, Tambra Dora, Lamar Box, Michelle Listz, Katherine Davis, Jennifer Montes, Jessica Ruff, Jennifer Leyva, Pamela Fidler, Ruth Fitch, Sean Turnbow, Francesca Vigil, Maria Barrientes, Isaiah Knight, Cindy Duran, Lauren Christal, Breana Wade Liaison, Brooke Harris, Dean Skiles, Marisol Ramos, Brandon Newsom, Candace Gaitan, David Pereira	Benchmark Research	Austin, TX

Stuart H. Cohen, MD	Curtis Blankenship, Katelyn Trigg, Courtney Lymuel, Gursimran Mann, Zayan Musa, Hana Minsky, Eliseo Vasquez, Nicole Garza, Kaitlyn Low, Mehrab Hussain, William Li, Rahul Araza, Monique Conover, George Thompson, Hien Nguyen, Scott Crabtree, Bennett Penn, Minh-Vu Nguyen, Archana Reddy, Derek Bays, Kaitlyn Hardin, Matthew Boutros, Alan Koff, Natascha Tuznik, Angel Desai, Naomi Hauser, Sarah Waldman, Gauri Barlingay, Dean Blumberg	UC Davis Health	Sacramento, CA
Erin Cooksey, MD	Kristina Harrison, Deanna Cooper, Manisha Horton, Amanda Philyaw	Synexus Clinical Research	Anderson, SC
Aurelio Cruz-Valdez, PhD	Janeth, Pacheco-Flores, Anyela Lara, Secia Diaz-Miralrio	Instituto Nacional de Salud Pública	Cuernavaca, México
Dima Dandachi, MD, MPH	Tami Day, Britlyn Brown, Taylor Mathews	University of Missouri Health Care	Columbia, MO
Matthew G. Davis, MD	Therese Dayton, Joseph I. Mann, Patricia S. Larrabee, Jean C. Kelly, Tia. L Albro, Zerina Zornic, Susan J. Willer, Donna M. Willome, Kathleen K. Ebeling, Jaclyn P. Zona, Julie A. Mooney, Katherine A. Pagenkemper, Victoria F. Fink, Christine N. Hall, Chelsea Bork, Abigail Miller, Mackay Kanaley, Chelsey LoMonaco, Marie Musolino, Jessica Fisher, Katilyn Bergen, Rachel Bordonaro, Cassidy Glod, Liam Sullivan, Brandi Douglass, Ann Casey, Philip LaSpino, Maurice Holmes	Rochester Clinical Research	Rochester, NY
Michael E. Dever, MD	Michael Delgado, Tameika Scott, Laverne Denise Davila, Nelisa Frias, Anissa Hilton, Patricia Brown, Shana Caldwell, Martha Hendrix, Edmund Delgado, Mitul Shah, Gracemarie Rosario, Kaneitra Williamson, Taylor Lucier, Jaime Hawat, Matthew Stephens, Monica Cooper, Dante Canidate, Denise Pagan, Sierra Robinson, Pascal Nelson-Quiles, Anthony Perez, Chanel Adams, Keisha Foster, Scott Salmon, Andrew Lockwood, Priya Moorhouse, Paul Yi	Clinical Neuroscience Solutions	Orlando, FL
Matthew Doust, MD	Stephanie Catanzaro, Shana Harshell, Madison Mikulak, Bettie, D'Nise Corcoran, Susan DeCraene, Jasmin Redden, Brian DeCraene, Karen Wakefield, Adrian Aljeo, Denise Sample, Clarissa Lara, Stephanie Junker, Nathan Alderson, Kimberly Joshlin, Mia Munoz, Michele Aguirre, Dina Reyes Cordova, Neil Pearson	HOPE Research Institute	Phoenix, AZ
Daniel Duncanson, MD	Kelly Kush, Lori Nesbitt, Cora Sonnier, Jennifer McCarter, Thomas Buschbacher, Evie Zavala, Brittany Cooper, Abbey Mannings, Melissa Berrio, Erin Juhl, William Douglas, Timothy Elder, Linda Grover, Colleen Crabbe, Rachel Francis, Jesse Lipnick, Seldon Longley, Michael Rozboril, Madison Duncanson, Jakob Vaes, Michael Costa, Dhruv Panchal, Michelle Hendricks, Sergio Montalvo, Angel Dubois	SIMEDHealth / SIMEDResearch	Gainesville, FL
David Ensz, MD	Bruce Rankin, Tavane Harrison, Meagan Miller, Kayla Sturgeon, Jessica Knight, Janet Otto, Monica Salazar, Megan Howard, Carly Deges, Joseph Harris, Rylea Gulick, Melissa Wiseman, Sue Doty	Meridian Clinical Research	Sioux City, IA
Brandon Essink, MD	Roni Gray, Christine Wilson, Fritz Raiser, Akossiwa "Essi" Yovogan, Jessica Satorie Tiffany Nemecek, Hannah Harrington, Amy Lett-Brown,Chelsea Steinmetz, Tabitha Campbell, Carrie Essink, Jamie Meyer, Riley Brockman, Melissa Monarrez, Troy Humphries, Wynter Huffman, Brooke Dworak, Raquel Davis, Samantha Nocita, Heidi Smith, Carissa Schejbal, Kayla Flege, Joe Genoways, Jessa Swanson, Avery Dunn, Kevin Grimes, Phillip Astorino, Ashtynn Jarosz, Hailey Harper, Amy Nichols, Azra Bauman, Jessica Fellows, Courtney Heisey, Ginny McNew	Meridian Clinical Research	Omaha, NE
Carlos Fierro, MD	Natalia Leistner, Amy Thompson, Celia Gonzalez, Nathan Arthur, Mazen Zari, Mary Easley, Heather Barker, Manyvohn Rinehart, Monica Atwood, Natalya Amrine, Kelly Moen, Kaley Miller, Angela Eichler, Ann Geier, Christa Estrada, Amber Wolf, Denise Essix, Latoria Rios, Kasie Hickert, Kenny Nguyen, Karol Moore, Stefanie Uwah, Kaelyn Howell, Miranda Dean	Johnson County Clin-Trials	Lenexa, KS
Kenneth Finkelstein, DO	David Beckmann, Tanya Hutchins, Sebastian Garcia Escallon, Kristen Johnson, Athena Rivera, David Otuada, Jessica Bartlett, Lauren Wade, Tyler Will, Gina Nielsen-Grewe, Anita Suri	Providea Health Partners Elligo Health Research	Evergreen Park, IL
Jon Finley, MD	Nathan Segall, Mildred Stull, Michelle Sowell, Michelle Binns, Kiara Tyner, Karen Yangapatty, Elizabeth West, Cynthia Steele, Kwannda Whatley, Hannah Smith, Pamela Talbott, Kimberly Cobb, Donna Toepfer, Jennifer LeBrun, Susan Jones, Patrizia Greene, Cynthia Pinckney, Kim Banaski, Karen Hickson	CRA Headlands	Stockbridge, GA
Diana F Florescu, MD	Mark Rupp, Daniel Brailita, Adia Sikyta, Erica Stohs, Sara Hurtado Bares, Nada Fadul, Matthew Lunning, Elizabeth Schnaubelt, Molly Ferris, Andrew Buettner, Matthew Palmer, Bailee Lichter, Alison Lewis, Chase Kimberling, Jonathan Beck, Erin Iselin, Kimmai McClain, Andrew Schnaubelt	University of Nebraska Medical Center	Omaha, NE
Patrick Flume, MD	Gary Headden, Brandie Taylor, Ashley Warden, Amy Chamberlain, Kim Spencer, April Rasberry, Angela Millare, Angel Darrow, Abbey Grady, Max Lento, Allison Patterson, Caitlan LeMatty, Jhonatan Diaz, Andrew Stephens, Emalee Wood, Destri Eichman, Annie Cribb, Annelise Kauffman<br>Charnele Handy, Elizabeth Poindexter, Moira Chance, Anna Miller, Elizabeth Dickinson, Andrea Boan, Erin Klintworth	Medical University of South Carolina	Charleston, SC
Veronica Garcia-Fragoso, MD	Maria Gabriela Becerra, Cecilia Mckeown, Lisa Holloway, Toni White, Bonnie Colville, Frederic Santiago, Teresa Becker, Shakira Barr, Chen Ho Yang, Tracy Kowalski, Danitra Glasper, Diana Chehab Nazanin Zarinkamar, Joanna Quezon, Maryam Rabbani, Sadaf Batla, Ayla Perez, Berenice Ferrero, Dean Jang, Biman Goswami, Dustin McFadden, Elton Oliveira, Enya Rentas-Sherman, Julian Edmonson, Laura Plaza-Grisanty, Olga Konshina, Rachely Araujo-Gutierrez, Scott Ward, Teodoro Seminario, Patricia Matute, Sauleha Husain, Akram Assaf, Elisa Moralez, Frances Saubon, Jenny Torres, William Fernandez, Ashraf Jafri, Amy Anderson, Saji Mathew Perinjelil, Waheeda Sureshbabu, Kara Sikes, Joel Cano, Kendra Rogers, Quiana Wilson, Karina Sainz, Abdeali Dalal, Leena Mir, Misbah Baloch, Shammarrian Hampton, Crystal Reese, Lucia Almaguer,<br>Felicia Ardoin, Deep Patel, Bernardo Martinez Leal, Faryal Mahmood, Ana Rueda, Norma Gonzalez, Stacey Montero, Chandra Tobin, Abyssinia Moges, Ari Amirkhosravi, Herman	Texas Center for Drug Development	Houston, TX

	Ortiz, Matthew Joseph, Parul Mehta, Zain Rizvi, Diego Carrington, Blessing Feliz-Okoroji, Moez Talpur, Robert Krbashyan, Simeen Khan, Mary Rogers		
George H. Freeman, MD	Esther Laverne Harmon, Marshall A. Cross, Kacie Sales, Catherine Q. Gular, Amanda Fronzaglio, Timothy O'Malley, Zaahin Huq, Jenna Johnson, Jessica Fuggett, Danielle Merian, Rita Quinn	Health Research of Hampton Roads	Newport News, VA
David L. Fried, MD	Lynne A. Haughey, Ariana C. Stanton, Lisa Stevens Rameaka	Omega Medical Research	Warwick, RI
Christopher Galloway, MD	Candice Montros, Lily Aleman, Samira Shairi, Robert Duran, Wesley Van Ever, Wasilah Suid, Sandra Torres, Taylor Rice, Wanda Estrada, Julie Castillo,Stephanie Cassidy, Ashleigh Ford, Thai Marie, Colon Maldonado, Amedaris Cordero, Zahra Somji, Rachel Morris	Headlands Research	Orlando, FL
Cynthia L. Gay, MD, MPH	David Wohl, Michelle Floris-Moore, Michael Herce, Danielle Clement, Arianna Morrison, Jan Busby-Whitehead, Michelle Hernandez, Zachary Willis, Allison, Burbank, Peyton Thompson, Chris Evans, Susan Pedersen, Becky Straub, Samantha Earnhardt, Erin Hoffman, Jonathan Oakes, Tevnan Keller, Victoria Rucinski, Camille O'Reilly, Kelsey Vollmer, Jennifer Rees, April Welch, Patti Vasquez, Joy Wannamaker, Tanailly Giralt Smith, India Pitts, Amanda Beaten, Ebony Harrington, Alex Bradley, Chidinma Okafor, Miriam Chicurel-Bayard, Kristina Shoffner, Polly Tsai, Chelsea Taylor, Susanne Hendersen, Emily Padgett, Debbie Pence, Jane Salm, Matt Campbell, Kirsten Haigler, Ekatherina Diadiuk, Mariam Ramzan, Pamela Miller, Julie Nelson, Nicole Maponga, Carmen Garcia, Charlie McGehee, Gloria Oyediran, Paul Alabanza, William Wolf, Hannah Munro, Rachael Turner, Dana Lapple, Grace Tillotson, Andrew Powell, Mandy Tipton, Catherine Kronk, Oesa Vinesette, Arti Malik, Kirby Caraballo, Maria Stetson, Charles West, Erin Cardot, Andy Thorne, Maria Bullis, William Zhao, Jennifer Thompson, Kristen Gray, Sarah Law, Holly Milner, Frederick Asamoah, Daniel Galeana, Marcia Gibson, Caressa Goss, Pamela Jones, Joshua Lee, Cheryl Hendrickson, Rachel Cook, Erin Daniel, Centhla Washington, Carolina Pastrana-Medina, Dayo Nylander-Thompson, William Johnson, Eliza Debose, Chloe Twomey, Rachel White, Grace Bailey, Hayley Meier, Jennifer Te Vazquez, Ascary Arias, Allison Castillo, Dynesha Perry, Gwen McKnight, Lucie Mangala, Jessica Gingles, Maggie Harman, Marie Oriol, Sean McMurray, Christy Litel, Noshima Darden-Tabb, Yerson Padilla, Danna Frederick	University of North Carolina	Chapel Hill, NC
Bruce C. Gebhardt, MD	Padma N. Mangu, Debra Beck Schroeck, Rajesh Kumar Davit, Gayle D. Hennekes, Donna Percy	Sterling Research Group	Cincinnati, OH
Carl P. Griffin, MD	Raymond Cornelison, Shanda Gower,William Schnitz, Angela Genovese, Ryan Morgan, Destiny S. Heinzig-Cartwright, April Green, Kim Hamilton, Chalimar Rojo, Lacey Dietz, Sharee Wright, Aja George, Karen Hames, Sharla Lister, Brandy Ball, Andrea Romero, Krystal Hightower, Dalia Tovar, Kim Calloway, Samelia Farni, Chris Hyatt, Linda Lopez, Kathi Shaw, Natacha Tull, Katelyn Hughes, Selwyn Oruh, Lauren Schwab, Samantha Ting	Lynn Health Science Institute	Oklahoma City, OK
Joseph Grillo, MD	Amy Potts, Julie White, Carla Bender, Debra Daugomah, Caitlin Harris, Brian White, Alannah Hill, Chelsea Lairson, Karen Blevins	Medical Research International	Oklahoma City, OK
Bernard Grunstra, MD	Donald Quinn, Shelby Olds, Phillip Claybrook, Amy Dye, Shai Perry, Joshua Bullen, Jennie Eller, Sandy Daggs, Nicole Everhart, Dennis Lee, Farrah Fuston	PMG Research of Bristol	Bristol, TN
Greg Hachigian, MD	Deborah Murray, Michael Cancilla, Logan Ledbetter, Masaru Oshita	Benchmark Research	Sacramento, CA
Charles Harper MD	Keith Vrbicky, Chelsie Nutsch, Sally Eppenbach, Wendell Lewis, Alisha Kiepke, Misty Appeldorn, Cyla Rohde, Catherine King, Kayla Andal, Ashley Frisch, Courtney Green, Kelsey Kelley, Katlyn Mace, Jordan Suckstorf, Torie Johnson, Linden DeBoer, Christy Lee, Eric Graber, Jeni Hoppe, Jill Smith, Heather Ebel, Taysha Hingst, Samantha Wieseler, Diahn Pekny, Elijah Schantz	Meridian Clinical Research	Norfolk, NE
C. Mary Healy, MD	Chianti Wade Bowers, Chanei Henry, Sheri Ordonez, Janet Brown, Cathy Faw, Shetel Anassi, Trent Davis, Kim Taylor	Baylor College of Medicine	Houston, TX
Jeffrey A. Henderson, MD, MPH	Jeffrey A. Henderson, MD, MPH, Marcia O'Leary, RN, Kendra Enright, RN, Jill Kessler, MS, Pete Ducheneaux, LPN, Asha Inniss, MS, APRN	Black Hills Center for American Indian Health / Missouri Breaks Industries Research Inc	Rapid City, SD
Ripley Hollister, MD	Jeremy Brown, Melody Ronk, Jill York, Shelby Pickle, Jami Wagner, Lisa Jackson, Felipa Ramdeholl, Angelica Romero	Lynn Institute of the Rockies	Colorado Springs, CO
Matthew Hong, MD	Wayne Harper, Lisa Cohen, Priti Patel, Kendra Lisec, Makayla Dutton, Lynn Eckert, Aubrey Farray, Jenee Jiggetts, Emily Reilly, Jill Holmes, Aaron Deaver, Christine Grissom, Judith Shand, Brianca Farmer, Eric Henderson, Kristen Shireman, Brad Muskelley, Franziska Gassaway, Darian Lawrance, Sabine Ucik, Toni Bland, Katedra Dixon, Reginald Santiago, Caroline Zhu, Kathleen Sander, Brian Joseph, Marsha Peery, Lori Bridges, Sadia Khan, Adnan Nasir, Sofia Sequiera, Raquell Messick, Kyra Brown, James Hull	M3-Wake Research	Raleigh, NC
Gregory Holt, MD	Jennifer Denizard, Juanita Johnson, Sehrish Sikandar, Gisel Urdaneta, Silvana Cobain, Melyssa Sueiro, Precious Leaks, Evelyn Guadalupe, Rochelle Thompson, Dexter Peart, Leidi Paez, Krystal Hosang, Runxia Tian, Ali Vaeli Zadeh	Miami Veterans Affairs Medical Center	Miami, FL
Lilly Immergluck, MD	LaKesha Tables, Harold Gene Stringer, Jacquelyn Ali, Cristina Wilson, Noor Mohamed, Kay Woodson, Tiffany White	Morehouse School of Medicine	Atlanta, GA
Michael Jacobs, MD	Kathleen Menasche, Vincent Mirkil,Yazil Ramirez, Michael Yee, Laura Elio, Candice Garcia, Azucena Valdovino, Cristina Garcia, Sharla Peahi-Ching, Kristina Arcos	Alliance for Multispecialty Research	Las Vegas, NV
Jeffry Jacqmein, MD	Maggie Bowers, Dawn Robison,Victoria Mosteller, Janet Garvey, Alpa Patel, Darlene Bartilucci, Kenneth Aung-Din, Margaret Gannaway, Carolyn Tran, Michael Koren, Mitchell Rothstein, Sonia Gerardo, Cassie Lawler, Yvonne Douglas, Chris Ganzhorn, Emery Noles,	Jacksonville Center for Clinical Research	Jacksonville, FL

	Angela Morris, Lisa Carl, Andrea West, Laura Little, Ramil Castillo, Abbey Ras, Nalini Jones, Annan Nurrenbern, Deirdre Arrington, Jacob Wolfer, Brenda Anderson, Amanda Elwood, Amber DeVries, Cara Seifart, Jimmy Knowles, Vy Dang, Mary Strickland, Pam Garmon, Caron Whitelaw, Sharon Smith, Ivy Guillermo, Nate Grant, Khatija Hussein, Caron Whitelaw RN, Bernadette Moineau, Robert Nix		
Robert Jeanfreau, MD	Susan Jeanfreau, Katelyn Jackson, Kynisha "Nicki" Johnson, RaeShanta McKendall, Shonna James, Calisha Sadiq, Susan Tortorich, Lori Goins, Steven Darden, Melissa Spedale, Kristen Robinson, Joseph Favret, Yordanka Koleva, April Spears, David Conroy	MedPharmics	Metairie, LA
William Jennings, MD	Hilario Alvarado, Michele Baka, Malina Regalado	Synexus Clinical Research	San Antonio, TX
Carol Kauffman, MD	Andrea Starnes, Andrea Woods, Karen Brudzinski	VA Ann Arbor Healthcare System	Ann Arbor, MI
Colleen F. Kelley, MD, MPH	Carlos del Rio, Sheetal Kandiah, Catherine Abrams, Erin Andrew, Felicia Atkinson, Erica Baker, Juliet Brown, Tucker Colvin, Natasha Renee Cook, Meena Dhir, Christopher Foster, Ronald Gaston, Gabriela Gerogial, John Gharbin, Betsy Hall, Valarie Hunter, Aastha KC, Kelly Likos, Bezuayehu Mandefro, Myles Mason, Humberto Orozco, Isaac Perez, Philip Powers, Christin Root, Brittany Spiegel, Pamela Weizel, Sarah Wiatrek, Felicia Wright	Ponce de Leon Center	Atlanta, GA
Jeff Kingsley, DO	April Pixler, LaKondria Curry, Sarah Afework, Austin Swanson, Alyssa Middlebrook, Christine Senn, Keyrhea Ritter, Katlin Salewski, Sierra Holmes, Jean Niles, Taylor Hernandez, Lacey Shaw, Kaila Maddox, Klarissa Bohnstedt, Emily Gilder, Cassandra Motley, Alyssa Middlebrook, Hephzibah Udo, Mattison Sherer, Wayman Petty, Joseph Surber	IACT Health	Columbus, GA
Judith Kirstein, MD	Marcia Bernard, Erica Sanchez, Nolan Mackey, Clarisse Baudelaire, Hanna He, Brenda Delgado, Brandon Steppe, Bonnie Goodale, Nicole Abels, Carol Remigio, Dipal Patel, Emily Zacarias, Nuvia Espinoza, Esmeralda Machado, Katia Talamante, Lizeth Romero	Velocity Clinical Research	Banning, CA
Susan Kline, MD, MPH	Sara Eischen, Rebecca Cote, Diondra Howard, Editha Jordan, Joyce Bolea, Annie McFarland, Asfaw Mesfin, Andrew Snyder, Darlette Luke, Derek LaBar, Theresa Christiansen, Beth Jorgenson, Christina Glasgow, Melissa Schedler	University of Minnesota	Minneapolis, MN
Mark E. Kutner, MD	Mark E. Kutner, Jorge Caso, Janet Mendez, Maria Hernandez, Carmen Amador, Amanda G. Colina, Alain Chang, Alondra Diaz, Arael Ayala, Carmen Ballester, Claudia Rodriguez, Dalila Del Valle, Eduardo Rodriguez, Gloria Moreno, Jennifer Ortega, Jhobana Vargas, Jonathan Fernandez, Juan Carlos Delgado, Laura Gonzalez, Leidy Montoya, Marianela Carvajal, Mariete Rendon, Maury Santos, Michelle Browne Mirnaya, Mujica, Neiner Enriquez, Noelio Hernandez, Paola Garcia<br>Raydel Valdes, Saray Carvajal, Susel M. Figueredo, Vanessa Hechevarria, Yanelis Dominguez, Yusleidy Diaz	Suncoast Research Group	Miami, FL
Mark Leibowitz, MD	Fernanda Morales, Rosario Sanchez, Mike Delgado, Norma Vega, Nelly Ayala, Iliana Gallaga, Cassandra Celis, Jennifer Muniz, Mariela Quiroz, Juan Frias, Rea Abaniel, John Nelson, Maricor Grio, Alejandro Moreno, Coralia Soto, Jose Espino, Daniel Vargas, Stephanie Lopez	National Research Institute	Los Angeles, CA
Derek Lewis, MD	Fred Newton, Aeiress Duhart, Breana Watkins, April Green, Chala Simpson, Briana Dean, Brandy Ball, Shakita Stevenson, Lashonda Stephenson	Lynn Institute of the Ozarks	Little Rock, AR
Gregg Lucksinger, MD	Jaleh Ostovar, Audrey Kuehl, Viviana Juncal, Avery Kerwin	Velocity Clinical Research	Medford, OR
Benjamin J. Luft, MD	Jorge Alves, Melissa Carr, Ryan Chacon, Barsha Chakraborhy, Aymon Faizi, Laurel Gumpert, Andrew Handel, Kayla Henkel, Erin Infanzon, Andrew Kanner, Lily Limsuvanrot, Michelle Miroddi, Jeanine Morelli, Sharon Nachman, Rena Nanan, Alexander Newman, Alison Pellecchia, Trisha Rush, Jennifer Russell, Stephanie Santiago-Michels, Jonathan Sicoli, Candace Smith, Michael Truhlar, Bruno Valenti, Jennifer Valentine, Kathy Vivas, Yasmine Brown-Williams	Stony Brook University - Stony Brook Medicine	Commack, NY
Siham Mahgoub, MD	Alice Ukaegbu, Immaculate Okonkwo, Shannon Gopaul, Tara Gibbons, Yuanxiu Chen, Debra Ordor, Linda Fletcher, Megan Ware, Florencia Gonzalez, Michael Perini, Carla Williams, Mulu Mengistab, Robert Postell, Yejide Obisesan, Adetokunbo Adedokun, Reyneir Magee, Jeremy Smith, Edward Bauer, Lora Collins, Urelda Allman, Deborah Clements, Sarah Shami, Nathaniel Blaboe, Pedro Lima, Michael Crawford	Howard University Hospital / Howard University College of Medicine	Washington, DC
Mary Beth Manning, MD	Toby Briskin, Denise Roadman, Sarah Dzigiel, Jennifer Gaston, Brooke Glivar, Brianna Arman, Briana Jackson, Brian Sharpe, Naqib Ahmad, Nicole Baitt	Velocity Clinical Cleveland	Cleveland, OH
Rickey D. Manning, MD	James Wilson Hurst, Rodney E. Sturgeon, Paul H. Wakefield, John A. Kirby	Accellacare	Knoxville, TN
Kristen Marks, MS, MD	Marshall Glesby, Roy Gulick, Timothy Wilkin, Ole Vielemeyer, Mary Vogler, Carrie Johnston, Rebecca Fry, Daniel Finn, Caitlin Rhoades, Noah Goss, Shaun Barcavage, Valery Hughes, Jonathan Berardi, Ashley Machado, Caique Mello, Mia Crowley, Monique Williams, Minkyung Lee, Mary Ann Zwiebel, Patrice Weller, Antonio Rivera-Lopez, Harrison Chan, Ruby Lee, Victoria Lesina, Vasilika Koci, Paul Kim, Steven Wang, Malissa Robinson, Edward Kenny, Danny Garcia, Venus Fernandez, Parul Shah, Celine Arar, Byron Bullough, Jonattan Rodriguez, Jessenia Fuentes, Jiamin Li, Arthur Goldbach, Genessi Rodriguez, Catherine Jerry, Nadi Islam, Madeline Gomez, Rajshri Hirpara, Ioanna Pahountis, Wayne Burns, Tahera Begum, Gianna Resso, Sophia Alvarez, Elizabeth Connolly, Roxanne Rosario, Sierra Derti, Britta Witting, Anna Gwak	Weill Cornell Chelsea CRS	New York, NY
Paul G. Matherne, MD	Cassie Beeks, Sarah Bowen, Deven Fejka, Nicole Guttierrez, Lakeyla Bates, Pam Taylor, Gigi Benoit, Micki Le	Medpharmics	Gulfport, MS
Monica McArthur, MD, PhD	Cheryl Young, Helen Powell, Levis Contreras, Panagiota Komninou, Christine Wade, Jumoke Oladapo, Kaitlin Mason, Robin Barnes, Leslie Howe, Cheilon Bolanos, Shannon Bittner, Elva Valle-Maldonado, Wanda Somrajit, Biraj Shrestha, Justin Ortiz, Nancy Greenberg, Kathleen Strauss, Lisa Chrisley, Melissa Billington, Sudhaunshu Joshi, Lavida Porter, Megan McGilvray, Daryl Grays, Shirley George, Jennifer Marron, Kelly Brooks, Natelaine Fripp, Mardi Reymann, Brenda Dorsey, Patricia Farley, Melissa Myers, Natasha	University of Maryland School of Medicine	Baltimore, MD

	Harris, Alyson Kwon, Marcela Pasetti, Daniel Cohen, Myounghee Lee, Laura Liberman, Sherry McCammon, James Campbell, Ana Herbert, Julia Silva, DeAnna Friedman-Klabanoff, Alythia Vo, Jennifer Winkler, Lisa Turek, Colleen Boyce, Anne Thurston, Daniele Nitkowski, Ginny Cummings, Sandra Molina, Susan Holian, Matthew Laurens, Rekha Rapaka, Megan Deming, Mark Travassos, Kirsten Lyke, Henry Seifert, George Escobar, Norma Martinez, Abigail Arias, Ana Maria Davila, Dolores Fontalvo, Elsa Aracely Vargas, Elva Jaldin, Gladis Lopez, Irma Justiniano, Judith Tenezaca, Julio Fernandez, Luz de Maria Osorio, Maria de los Angeles Pichardo, Morena Lemus, Maria Elena Rocha, Rosa Angelica Vigil, Sandra Herrera		
R. Scott McClelland, MD, MPH	Devinder Garcha, Christopher P. Hawk, Bonnie Duran, Donna Lodge-Moore, Leigh Tao, Cristina J. Toledo-Cornell, Mona Jalili, Susan Lottimer, Sheila Samra, Seslee Alsup, Karlee Cooper, Theresa George-Greene, Spencer Hanson, Joni Hensley, Emily Barnett Highleyman, Jewell Jefferson, Jessica Lane, Jessica Long, Alex Martinez, Kerri Sloan, Kelly Smith, Kristee Lewis, Tara Babu, Dwyn Dithmer, Matthew Dustrude, McKenna Eastment, Emily Ford, Abir Hussein, Christine Johnston, Pamela Kohler, Debra Metter, Thepthara N. Pholsena, Meredith Potochnic, Tara Reid, Miko Robertson, Michelle Sabo, Helen Stankiewicz Karita, Jina Taub, Dana Varon, , Brian Wood, Alyssa Braun, David Crawford, Mark Drummond, Jess Heimonen, Lawrence Hemingway, Madelaine Humphreys, Bianca Kalia, Mary Kirk, Taylor Krause, Ray Larsen, Gisella Logioia, Cristina Luevano Santos, Anya Mathur, Lindsey McClellan, Jessica Moreno, Nicole Roed, Matthew Seymour, Katie Wicklander	University of Washington & Lummi Tribal Health Center	Seattle, WA
Bruce McClenathan, MD	Mary Hussain, Aaron Poch, Amy Santangelo, Anne Poch, David De Blasio, Evelyn Lomasney, Jacob Turnquist, Kathryn Lago, Laurie Housel, Sherry Lamberth, Sheryl Bedno, Lauren Blevins, Laura Brown, Alice Clay, Gervon Collins, Kaitlyn Covington, Amy Davis, Patricia Davis, Nicole Friedberg, Lacey Gazlay, Helen Gooden, Evelyn Hall, Kim Locklear, Kayla Majors, Shamona McRae, Bryce Meerhaeghe, Kendalyn Stephens, Jade Tran, Lisette Watkins, Katie Williams, Kema Matthews, Karrie Greive, Brandi Carroll, Amanda Williams, Brittany Garner, DeLisa Crosby, Jennifer Ritschl, Jamie Frahm, Karen Stewart, Priti Patel, Dilay Uras, Allison Northrop, Anika Uson, Olyvia Ray, CynDavia McKoy, April Beals, Deidre Turner, Christina Spooner	Womack Army Medical Center	Fort Bragg, NC
Mark McKenzie, MD	Teresa Deese, Christy Schmeck, Vickie Leathers, Christy Sweet, Misti Earwood, Erica Osmundse, Gisela Heintz, Lilian Nunkuna, Michelle Forgey, Shelly Brooks, Justian Jarrett, Elizabeth Michael, Lisa Guider, Zack Harmon, Diane Sproles, Randy Cooper, Jessica Benvenuto, Stefanie Mullins, Quinetrice Bennett, Corey Flack	WR Clinsearch	Chattanooga, TN
Jorge F. Méndez Galván, MD	Adriana Sordo Durán, Martha Yarelli Valencia Mejía, Froylan David Martínez Sánchez, Ana María Piña Rodríguez, Diana Alim Mena Martínez, Melany Susel Fernández Valdez, Laura Ruy Sánchez Guerrero, Ana Fabiola Ruiz Villagrana, Mónica B. Carrascal, Martha Cecilia Gomora Madrid, Anahí García Álvarez, Ismael Delgado Ginebra, Omar Alfonso Heredia Nieto, Yanni Maldonado Ventura, Jonathan E. Ramírez Salazar, Mariela Salgado Zagal, María Fernanda Espinosa García, Yolanda Albor Hernández, Ricardo Antonio González, Germán Alonso Lara, Marbella Rojas Ortega, Bernardo Kleinfinger Chayet, Victor Emmanuel Alva López, Diego Carlos Angel Perez, Alejandro Cortes Meda,Ivonne Hernandez Giron	Centro de Atención e Investigación Médica (CAIMED)	Mexico City, Mexico
Rajan Merchant, MD	Anelgine Crans Yoon, Janet Hill, Lucy Ng-Price, Teri Thompson-Seim, Alejandra Cazares Hernandez, Danielle Hornbuckle, Adriane Rubit, Ann Campbell, Dawn Diorio, Adeline Stabler, Jasdeep Shergill, Claudia Gross, Anne Nguyen	CommonSpirit Health Research Institute	Woodland, CA
Vicki E. Miller, MD, MPH	Amy Starr, Shiela Varghese, Sonia Guerrero, Monica Murray, Vanessa Gonzales, Blanca Gomez, Zainab Rizvi, Victoria Aguilar, Anna Pena, Madiha Baig, Dustin Watson, Pauline Ngban, Afifah Ayub, Laura Drampou, Shelby Danforth, Diana Avalos, Jacquelyn Gonzales, Ragen Powell, Sajjad Naqyi, Ambily Dileep, Alefiyah Motiwala, Heather Leary, Humera Siddiqui, Miatta George, Kastyn Kelly, Nicole Segura, Maryam Jamil, Husain Motiwala, Sandra Smith, Sally Hussein, Yousra Yousif, Carlyn Robinson, Cannon Lenfield, Luis Leal, Muhammad Irfan, Nayab Croher, Pattie Tate, Sandra Natalia Perez, Fredric Santiago, Syeda Riaz, Arsani Iskandar, Alefya Hussain	DM Clinical Research	Tomball, TX
Linda Murray, DO	Christy Delcamp, Monica Hoewt, Kristin Shade, Tara McTigue	Synexus Clinical Research	Pinellas Park, FL
David Musante, MD	William P. Silver, Linda R. Belhorn, Nicholas A. Viens, David Dellaero, Shandelle Parker, Andrew Zimmerman, Roger Ordronneau, Bryan Stanislaus, Kevrin Johnson, Megan Dice, Megan Heron, Sarah Wilkerson	M3-Emerging Medical Research	Durham, NC
Sherif Naguib, MD	Justin Singletary, Sha-Wanda Richmond, Sarah Omodele, Emily Oppenheim, Jalisha Hemphill, Marqueta Jones, Millat Gedefa, Janean Smith, Bonnie Raufman, Lesley Whitehead, Elia O'Dell, Sarah Omodele, David Taylor, ShaWanda Richmond, Alexis Melson, Justin Singletary	Synexus Clinical Research	Atlanta, GA
Joseph Newberg, MD	Laura Pearlman, Reuben Martinez, Victoria Andriulis, Jacquilyn McCormick, Anna Maddox, Rosalinda Vazquez, Nicole Leahy, Marian Padilla, Mary Reyes	Synexus Clinical Research	Chicago, IL
Paul J. Nugent, DO	Leonard Singer, Jeanne Blevins, Meagan Thomas, Christine Hull	Synexus Clinical Research	Cincinnati, OH
Yaa D. Oppong, MD	Ryan P. Starr, Scott N. Syndergaard, Nafisa Saleem, Cheryl Norris, Nicole Austin, Rozeli Shelly, Md Mashrur Islam Majumder, Annette Bunnells, Michelle Wallace, Avia McClain-Stocker, Rachel Ryan, Katie Wood, Arien Stebbins, Crystal Schmitt, Jeffrey Pemberton, Mitchel Arlidsen, Daniel Tomita, Geraldine McRae, Amy Sheets, Jeanette Mangual-Coughlin,<br>Margo Miller-Smith, Melinda Thomas	Carolina Institute for Clinical Research	Fayetteville, NC
J. Scott Overcash, MD	Adrienna Marquez, Hanh Chu, Kia Lee, Kim Quillin, Jordan Coslet, Yashveer Dubbula, Adam Prince, John Rodriguez, Lee Tomatsu, Erin Vawter, Michael Voskanian, Michael Waters, Gina Weaver, Karla Zepeda, Angela Anorve, Gordon Bovee, Jennifer Baker, Laura Castillo, Allie Davis, Jacob Esparza, Andrea Garcia, Jessica Gonzales, Lizette Gonzalez,	Velocity Clinical Research	La Mesa, CA

	Ashleigh Lindsay, Erica Marinelli, Cathy Meza, Shandel Odom, Makenna Orel, Grecia Perez, Helen Pu, Cesar Ramirez, Melania Riordan, Deidre Romines, Raquel Taitingfong, Katrina Tyler, Bernadette Wilson		
Yogesh K. Paliwal, MD	Amit Paliwal, Renu Bhupathy, Krystle Edwards, Sarah Gordon, Cynthia Montano-Pereira, Blanca Gomez, Yazmin Nunez, Cassandra Martinez, Connie Navarrete, Mayra Casas, Ysabel Lopez, Anthony Macias, Alexandria Vasquez, Maria Gomez	Empire Clinical Research	Pomona, CA
Isabel Pereira, MD	Gina Rivero, Tracy Okonya, Frances Downing, Paulina Miller, Yasmin Camberos	Synexus Clinical Research	Vista, CA
Bruce Rankin, DO	John M. Hill, Steven Shinn, Vivek Rajasekhar, Marshall Nash, Michelle Tutt, Kimberlee Del Campo, Douglas F. Winter, Leandro Fernandez, Melissa Hodges, Michelle Jones, Sean Lemoine, Veronica Walker, Roy D. Richardson, Angeline Petracca. Katina Marchione, Michelle Morgan, Ashley McCaffrey, Amber Vasquez, Amy Houck-Dominy, Angela Hammerle, Antonio Rivera, Claxton Copeland, Crystal Paccione, Diana Toney, Fadhel Alyunis, Jennifer Dittman, Kriston Applewhite, Lora Parahovnik, Over Seijas, Ryan Hobbick, Samantha Watts, Shatonia Fields, Stacie Evans, Teresa Logsdon, Thais Truffa, Tifany Huertas, Vienna Bauer,William Serrano, Daisy Sawyer, Giovanni Urquilla, Tonya Toby, Albert Garcia, Alicia J. Cevera,Jeffery Hood, Hannah Hodges, Melissa Willard	Accel Research Sites	DeLand, FL
María José Reyes Fentanes, MD	Pablo Fermín González Limón, Luis Ricardo Acosta Beuló, Paulina Cleer García Valdovinos, Olivia de la Puente Flores, Eduardo Rugama Martel, Ana Gabriela Mier Flores, Ulises Abel Rodríguez Vargas, Diego Guillermo Muñoz Bolaños, Martha Alejandra Alonso Trejo, Elvia Ramírez Gutiérrez, Alberto Aaron del Rosal Medina, Jaime Chavez Baron, Ana Gabriela Guizar Zamora, Felipe Arredondo Saldaña, Juan De Dios Martín Luján Palacios, Juan José Pardo Moreno, Jorge Torres Ferrera, Itzetl Guzman Mendieta	PanAmerican Clinical Research México	Querétaro, Mexico
Margaret Rhee, MD	Jeffrey Klein, Katherine Stapleton, Stacy Collins, Dawn Greer, Kelli Meissner, Brenda Moore, Tylene Falkner, Celeste Blazy, Nicole Johnson, Christina Carter, Annette Pangle, Rosamond Hong	Synexus Clinical Research	Akron, OH
Robert Riesenberg, MD	Robert Riesenberg, Stanford Plavin, Mark Lerman, Leana Woodside, Maria Johnson	Atlanta Center for Medical Research	Atlanta, GA
Barbara Rizzardi, MD	Michelle King, Vanessa Abad, Jennifer Knowles, Benjamin Richeson, Denise Pessetto, Heather Holtman, Lori Luth, Wyatt Walsh, Andrea Johnson, Dreama Fackrell, Patrick O'Keefe, Sara Isolampi, Michelle Walkingshaw, Josh Carrillo, Renu Landage, Stephanie Wallace	Velocity Clinical Research	West Jordan, UT
Carina A. Rodriguez, MD	Patricia Emmanuel, Lucy Guerra, Asa Oxner, Alicia Marion, Reed Ryan, Tiffany Vasey, Susannah Hall, Amanda Morton, Emma Gonzalez, Elisabeth Ballans, Rachel Karlnoski, Luz Santamaria, Rosalinda Cruz, Joshua Finley, Michael Hayes, Oliver Emberger, Mark Pennington, Meghana Vankatesh, Kimberly Johnson, Marina Wassif, Janelle Perkins, Veroniya Winkfield, Amavyvis Garcia, John Jones, Lori Brock, Kyle Cesareo, Dilcina Dragon, Dominic Moore, Catherine Marten, Thi Nguyen, April Roberts, Kristi Bojaxhi, Chrestenie Mouse	University of South Florida, Morsani College of Medicine	Tampa, FL
David Rosenberg, MD	Lee Tomatsu, Viviana Gonzalez, Millie Manalo, Nicole Rudin	Pharmacology Research Institute	Los Alamitos, CA
Vida Veronica Ruiz Herrera, MD	Vida Veronica Ruiz Herrera, Eduardo Gabriel Vazquez Saldaña, Laura Julia Camacho Choza, Karen Sofia Vega Orozco, Sandra Janeth Ortega DominguezMaria, Carolina Molina Roman, Julian Camacho Choza, Rodolfo Fabian Lomeli Guerrero, Giuliana Magaña Garcia, Carlos Andres Perez Navarro, Cesar Alberto Lopez Martin, Luis Arturo Rico Godinez, Felipe de Jesus Lopez Cordova, Daniel Arroniz Bernal, Luisana Aldaco Cota, Edgar Cordova Pulido, David Aguila Rivera	PanAmerican Clinical Research México	Guadalajara, Mexico
Beth Safirstein, MD	Luz Zapata, Lazaro Gonzalez, Evelyn Quevedo, Farah Irani, Julio Vigil, Steven Rapp, Mark Firestone, Humberto Mucientes, Ali Yasells Garcia, Florence Baum, Robert Hacman, Martha Ravelo, Carlos Alzate, Keyanna Francois, Alberto Napoles, Jamie Lorenzo, Deandra Clarke, Disneydi Gutierrez, Yean Alfonso, Nestor Lopez, Ana Bustos, Ilya Faybishenko, Lynnette Perez, Evelyn Qulies, Maria del Valle, Natalie Joseph, Judith Powell, Jessica Hernandez, Rafael Sanchez, William Torres, Damaris Alonso, Dragos Juravle, Roberto Valledor, Veronica Valledor, Maria Pazos, Teresa Rios, Maria Lascano	MD Clinical	Hallandale Beach, FL
Howard Schwartz, MD	Nelia Sanchez-Crespo, Terry Piedra, Barbara Corral, Jennifer Schwartz	Cenexel RCA	Hollywood, FL
Elizabeth Secord, MD	Roy Collins, Marita Poff, Jamal Chehab, Sajith Matthews, Thomas Mazzocco, Chantel Karmo, Sarah Meram, Janie Faris, Valerie Mika, Shobi Mathew, Brian O'Neil, James Paxton, Amy Stolinski, Stacie Smith, Benjamin Wasinski, Lisa Palmer, Katherine Cross, Samuel Ceckowski, Theodore Falcon, Jeffrey Harrison, Abe Lovelace, Selmir Mahmutovic	Wayne State University	Detroit, MI
Marian E. Shaw, MD	Mark A. Turner, Cory J. Huffine, Esther S. Huffine, Jacqueline Hanson, Nicholas Tuttle, Shannon Veach, Antonio Navarrete, Jammie Smith	Velocity Clinical Research	Meridian, ID
Teresa S. Sligh, MD	Scott Sligh, Parul Desai, Vincent Huynh, Carlos Lopez, Erika Mendoza, Dennis Perez, Samuel Ceballos, Jennifer Gomez, Janneth Becerra, Tiffany Martinez, Erika Navarro Fausto	Providence Clinical Research	North Hollywood, CA
Joel Solis, MD	Carmen Medina, Westley Keating	Centex Studies	McAllen, TX
Jonathan Staben, MD	Jessica Horton, Hannah Neill-Gubitz, Hilary Koenigs, Autumn Dlugas, Stacie Rebar, Anne Reedy, Roslyn Pierce, Kali Karst, Jaimee Gribben, Sarah Troutt, Mimi Meipel, Ann Carson, Paige Ramos, Natosha Hardy, Zack Brownell, Dot Heid, Annie Estes, Andrea Fry, Veronica Navarro	MultiCare Institute for Research and Innovation	Cheney, WA
Kathryn E. Stephenson, MD, MPH	Karen A. Lorenc, Audrey B. Nathanson, Michelle Beck, Shaelah M. Huntington, Wendy Hori, Uyen Rasphoumy, Ashley Beckles, Jody Dushay, Vijai Bhola, Wilanda Gabriel, Annika Gompers, Halle Hall, Nicholas Manickas-Hill, Toluwanimi Ajayi, Nicole Magner, Conor Cronin, James Arrico, Heena Patel, Janet Mullington, Michael Seaman, Katherine Yanosick, Ariana Leonelli, Eric Dai	Beth Israel Deaconess Medical Center	Boston, MA
Danny Sugimoto, MD	Jeffrey Dugas Sr., Dolores Rijos, Sandra Shelton, Stephan Hong	Cedar Crosse Research Center	Chicago, IL
Suzanne Swan, MD	Sharine Phan, Tami Wahlin, Elizabeth Bennett, Amy Salzl, Jeannette Blaisdell, Stacie Mahowald, Dominick Thibodeau, Sophia Houser, Tammy Hanson	Synexus Clinical Research	Richfield, MN

Karen Tashima, MD	Helen Patterson, Stacey Chapman, Giselle Pinto, Jennifer Brashears, Evelyn Hipolito, Laura Elmasian, Timothy Flanigan, Joseph Garland, Britt Harrington, Anthony Harrison, Jenny Thai, Mazen Taman, Krista Kiser, Kay Rutherford, Shivani Patel, Jimin Shin, Kim Rapoza, Sujata Sahu, Kristine Hauser, Kendra Vieira, Elliott Bosco, Christopher Federico, Kanika Malani, Christian Schroeder, Janet O'Connell, Meghan McCarthy, Anna Hippchen	The Miriam Hospital	Providence, RI
Barbara S. Taylor, MD, MS	Bhoja Katipally, Jessica Blower, Kimberly Kone Ellis, Heta Javeri, Danielle Dixon, Anna Taranova, Diana Cavazos, Robin Tragus, Irma Scholler, Lisa Longoria, Laura Najvar, Meredith Hosek, Bridgette Soileau, Morgan Brown	University of Texas Health Science Center San Antonio	San Antonio, TX
Christine B. Turley, MD	Lewis McCurdy, Tonisha Brown, Martha Pawlicki, Jennifer Reeves, Jona Bauer, Cedrick Griner, Cameron Russell, Veena Sampathkumar, Zeynep Alimchandani, Robin Muller, Tracey Coakley, Mary Sours, Saifelnasr Mohamed, Sone Alanoh, Amy Yeh, Sahra Khan, Eleojo Abutu, Genena Buck, Sarah Hicks, Andreana Alexander, Tammy Patterson, Maria Martilnsalaco, Amy Clontz, Marina Leonidas, Zainab Shahid, Jay I. Patel, Ryan Bender	The Charlotte-Mecklenburg Hospital Authority d/b/a Atrium Health	Charlotte, NC
Lisa S. Usdan, MD	Lora J. McGill, Valerie K. Arnold, Carolyn Scatamacchia, Codi M. Anthony, Carol R. Marsh, Cathy T. Houpt, Charles L. Grandberry, Debra A. O'Brien, Kelsey N. Evans, Leslie M. Lazar, Mary J. Williams, Megann F. Fickle, Robyn M. Presley, Shelby R. McWhorter, Julia Sinatra, Irene W. Powell, Tavia S. Flagg, Melissa N. Flowers, Penny J. McCracken, Reagan A. Boone, Dominique L. Ross, Amber J. Jones, LaKeshia N. Pipkin, Victoria J. Neal, Monica Toor, Brandi Gruber, Erin L. Wells, Kelly Iskiwitz, Carolyn J. Scatamacchia, Codi M. Anthony, Lisa S. Usdan, Lora J. McGill, Valerie K. Arnold	Clinical Neuroscience Solutions	Memphis, TN
Larkin Tyler Wadsworth III, MD	Horacio Marafioti, Lyly Dang, Lauren Clement, Kristen Johnson, Anya Penly, Elizabeth Garner, Angie Kean, Sophia Bolakas, Andrea Deffenbaugh, Cerece Miles, Lindsay Nooter, Christy Shultz, George Cherniawski, Stephanie Tesson, Ash Dale, Laura Hartupee, Breanna Galibert, Karen Knapp	Sundance Clinical Research	St. Louis, MO
Michael Waters, MD	Dalia Tover, Scott Overcash, Jordan Coslet, Michael Voskanian, Giuliano Zolin, Matthew Petro, Gina Weaver, Kia Lee, Hanh Chu, Karla Zepeda, Crystle Rajania, John Rodriguez, Tracey Fabrega, Kaitlyn Sandler, Alex Tapia, Cecilia Barbabosa, Renee Pasion, Jacob Pineda, Rosalynn Landazuri, Angelica Franco, Estee Garcia, Marilynn Rodriguez, Joanna Ocampo	Velocity Clinical Research	Chula Vista, CA
Jordan Whatley, MD	Jordan Whatley, Christopher Dedon, Emily Best, Amie Breaux Shannon, Mary Margaret Dobson, Nicole Harrell, Lindsey Kobetz Hall, Kristen LeBleu Losavio, Patricia Whatley, Tana Bourgeois, Alexandra Caillouet, Samantha Brooke McMillon, Amy Thomassie, Donna Michelle Hurst, Michelle Symms, Lyndsea Folsom, Crystal Rowell, Loney Girod, Lauren Sternfels, Makaylea Truitt, Lori Martin, April Mims	Meridian Clinical Research	Baton Rouge, LA
Jewel Johnny White, MD	Amanda Occhino, Ruth Paiano, Morgan McLaughlin, Elisa Swieboda	Synexus Clinical Research	The Villages, FL
Hayes Williams, MD, PhD	LaShondra Cade, Mitzi Roberts, Aileen Cunningham, Rhodna Fouts, Connie Moya, Gary Boyd, Justina Owens, Abby Wellinghurst	Achieve Clinical Research	Birmingham, AL
Clint Wilson, MD	Jason Milligan, Danielle Raley, Joseph Bocchini, Carrie Kay, Shannon Saksa, Courtney Harmon, Ashley Primos, CJ McKenna, Star Roberts	Willis-Knighton Health System / WKB Family Medicine Associates	Bossier City, LA
Peter J. Winkle, MD	Amina Z. Haggag, Elizabeth Lee, Michelle Haynes, Marysol Villegas, Sabina Raja, Mary Grace Lejarde, Caroline Villanueva, Natalie Ureno, Jessica Cramer, Steven Garcia, Yesenia Barraza, Ashley Barajas, Lauren Ferreira, Lucy Rems, Zaki Abawi, Damon Pineda, Patricio Ordonez, Gaby Huizar, Lesbia Alarcon, Anna Luz Belarmino, Nenita Llarena, Alberto Heshike, Isabel Rangel, Karen Cruz, Rynel Villanueva, Matthew Rohrig, Han Tran, Axl Dyer, Maria Webb, Akihisa Kodama, Cynthia Juarez, Sandra Gaona, Moriah Wilson, Mark Gonzalez	Anaheim Clinical Trials	Anaheim, CA
Patricia L. Winokur, MD	N/A	University of Iowa Medical Center	Iowa City, IA
Paul E. Wylie, MD	Renea Henderson, Natasa Jenson, Fan Yang, Amy Kelley, Kelly Knight, Jessica Watson, Stacy Tierney, Emily Knight, Jessica Woosley, Faith Fields, Glen Scott Thrower	Preferred Research Partners	Little Rock, AR
Carmen D. Zorrilla, MD	Carmen Irizarry, Gloria Martino, Natalia Muler, Lázaro Valdés	Universidad de Puerto Rico - Recinto de Ciencias Médicas - Maternal Infant Studies Center (CEMI)	San Juan, Puerto Rico

United States Government (USG)/Coronavirus Prevention Network (CoVPN) Biostatistics Team

Affiliation	Team Members
Biomedical Advanced Research and Development Authority (BARDA), Washington, DC	Di Lu, James Zhou
Department of Biostatistics and Bioinformatics, Rollins School of Public Health, Emory University	David Benkeser, Sohail Nizam
Vaccine and Infectious Disease Division, Fred Hutchinson Cancer Center, Seattle, WA	Jessica Andriesen, Bhavesh Borate, Lindsay N. Carpp, Andrew Fiore-Gartland, Youyi Fong*, Peter B. Gilbert*, Ying Huang*, Yunda Huang*, Ellis Hughes, Ollivier Hyrien, Holly E. Janes*, Michal Juraska, Yiwen Lu, April K. Randhawa, Brian Simpkins, Brian D. Williamson*, Lars W.P. van der Laan, Chenchen Yu
Biostatistics Research Branch, NIAID, NIH, Bethesda, MD	Michael P. Fay, Dean Follmann, Martha Nason
Department of Biostatistics, University of Washington, Seattle, WA	Marco Carone, Avi Kenny, Kendrick Li, Wenbo Zhang
Department of Statistics, University of Washington, Seattle, WA	Alex Luedtke
Division of Biostatistics, School of Public Health, Department of Population Health Sciences, Weill Cornell Medicine, New York, New York	Nima S. Hejazi
Department of Population Health Sciences, Weill Cornell Medical College, New York, New York	Iván Díaz

*YF, PBG, YiH, and HEJ are also affiliated with the Department of Biostatistics, University of Washington, Seattle, WA. PBG and YuH are also affiliated with the Public Health Sciences Division, Fred Hutchinson Cancer Research Center, Seattle, WA. YuH is also affiliated with the Department of Global Health, University of Washington, Seattle, WA. Brian D. Williamson is also affiliated with Kaiser Permanente Washington Health Research Institute, Seattle, Washington, USA.

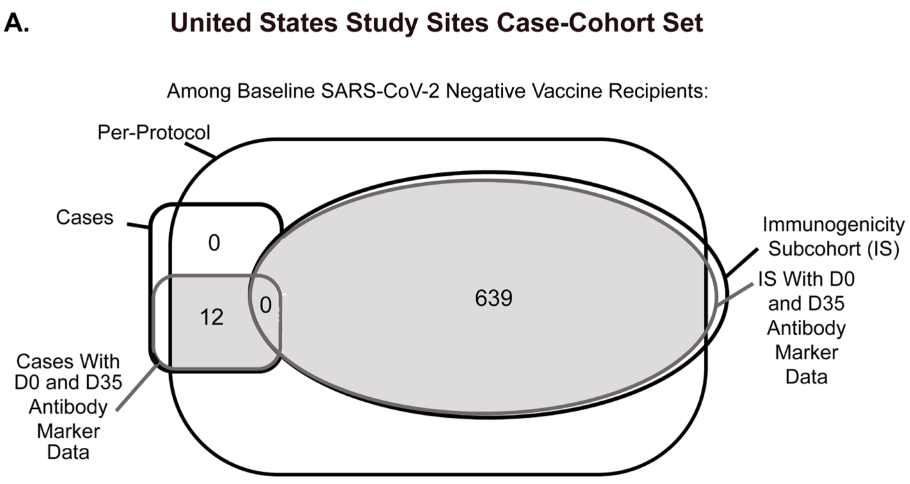

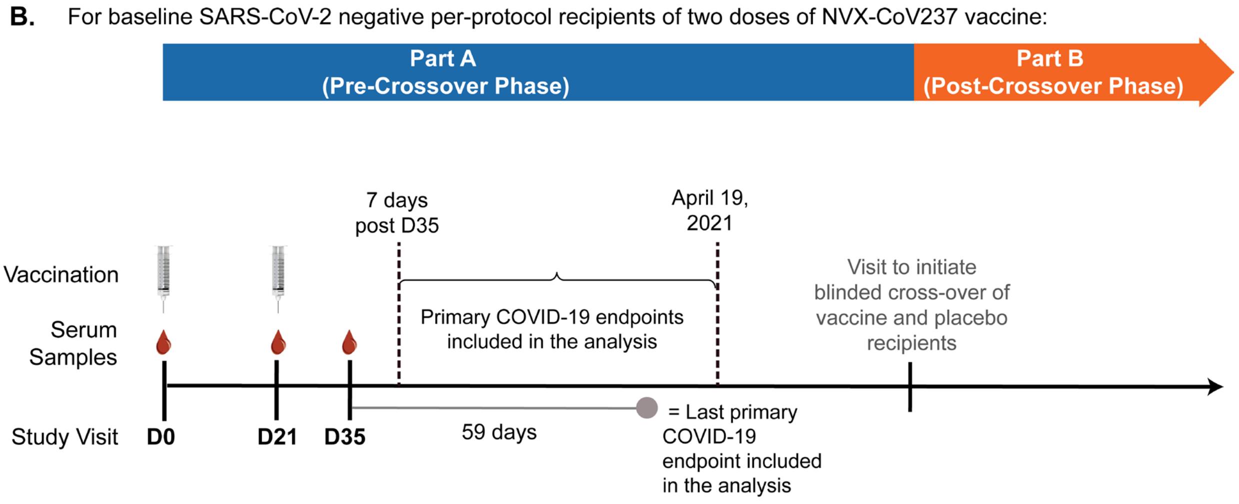

Supplementary Figure 1. (A) Case-cohort set (U.S. study sites). (B) Phases of the PREVENT-19 trial, timing of NVX-CoV2373 doses and serum sampling, and the time period for COVID-19 primary endpoints included in the Day 35 marker correlates analysis (correlates analyses restrict to Part A Pre-Crossover Phase and U.S. study sites). In (A), cases are baseline SARS-CoV-2 negative per-protocol vaccine recipients with the primary COVID-19 endpoint starting 7 days post D35 visit through to the efficacy data cut (April 19, 2021). "Baseline SARS-CoV-2 negative" is defined as in ref.[1], i.e. seronegative for anti-SARS-CoV-2 nucleoprotein and SARS-CoV-2 RNA RT-PCR-negative nasal swab at baseline. "Per-protocol" is also defined as in ref.[1], i.e. received both planned vaccinations, had no specified protocol deviations, and were SARS-CoV-2 negative on the D21 visit. Primary COVID-19 endpoints were as in ref.[1]: RT-PCR–confirmed symptomatic COVID-19 occurring at least 7 days after dose two. As all breakthrough cases occurred in the United States in Part A as of the efficacy data cut date, the case-cohort set (Panel A) was restricted to the U.S. cohort and therefore all primary COVID-19 endpoints included in the analysis (Panel B) were in the U.S.

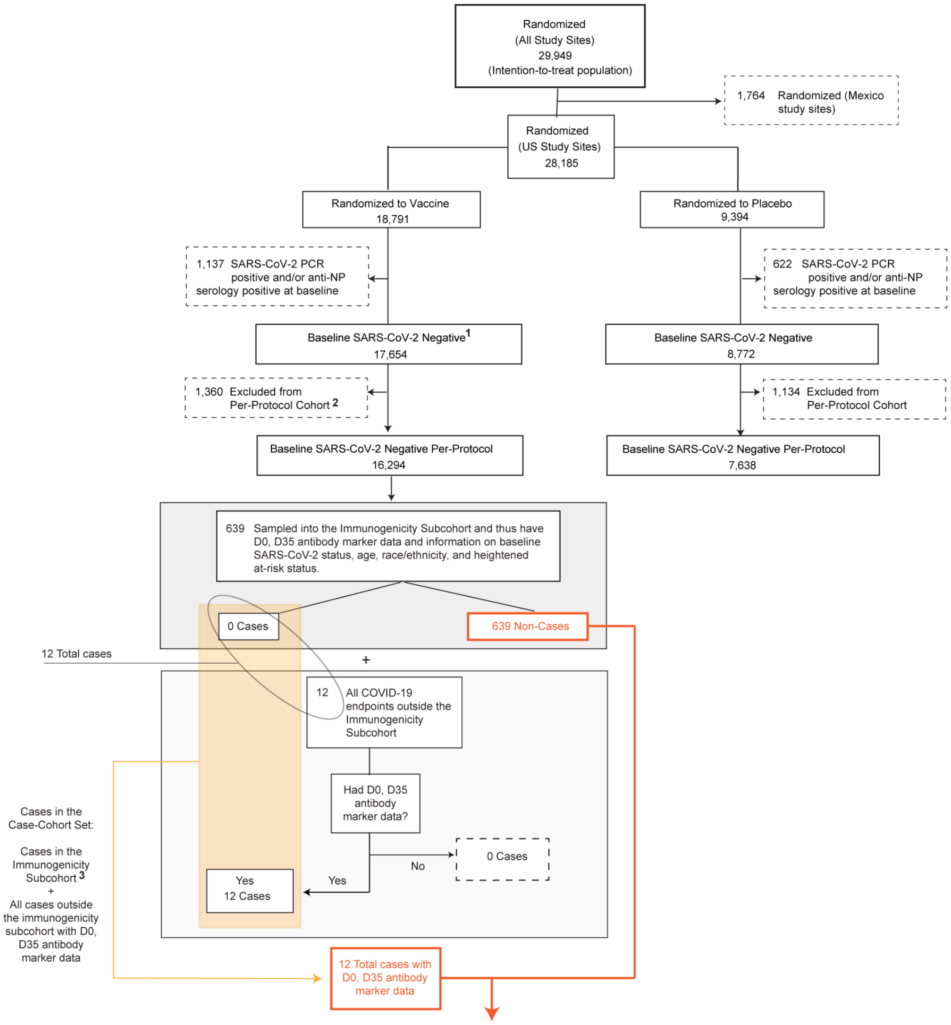

United States Study Sites
Case-Cohort Set

Baseline demographic covariate strata

	1	2	3	4	5	6	7	8	Total
Minority 4	N	N	N	N	Y	Y	Y	Y	
Age	18-64	18-64	≥65	≥65	18-64	18-64	≥65	≥65	
Co-existing conditions 5	N	Y	N	Y	N	Y	N	Y	
Cases	2	1	1	1	3	4	0	0	**12**
Non-Cases	102	97	89	80	74	73	55	69	**639**

1 Participants with missing baseline SARS-CoV-2 RT-PCR or anti-NP (nucleocapsid protein) serology data were considered baseline negative for the corresponding assay. After this, "baseline SARS-CoV-2 negative" was defined as baseline SARS-CoV-2 RT-PCR negative **and** baseline anti-NP serology negative.
2 Reasons for exclusion from per-protocol included: Did not receive two doses, doses out of allowed window, major protocol deviation.
3 There were no cases in the immunogenicity subcohort.
4 Minority includes Blacks or African Americans, Hispanics or Latinos, American Indians or Alaska Natives, Native Hawaiians, and other Pacific Islanders. Non-Minority includes all other races with observed race (Asian, Multiracial, White, Other) and observed ethnicity Not Hispanic or Latino. Therefore Unknown and Not reported have missing values for this.
5 Co-existing conditions are the same as those listed in Table 1 of Dunkle et al. NEJM 2022: obesity (defined as a body-mass index [the weight in kilograms divided by the square of the height in meters] of ≥30.0), chronic lung disease, diabetes mellitus type 2, cardiovascular disease, or chronic kidney disease.

Supplementary Figure 2. Flowchart of study participants from randomization through membership in the baseline SARS-CoV-2 negative per-protocol case-cohort set (U.S. study sites). Membership in the case-cohort set required availability of D0 and D35 antibody data and no evidence of SARS-CoV-2 infection through 6 days post D35. Antibody data from the placebo arm are not used in correlates analyses, given no variability in values; they were only used to verify low false positive rates of the immunoassays.

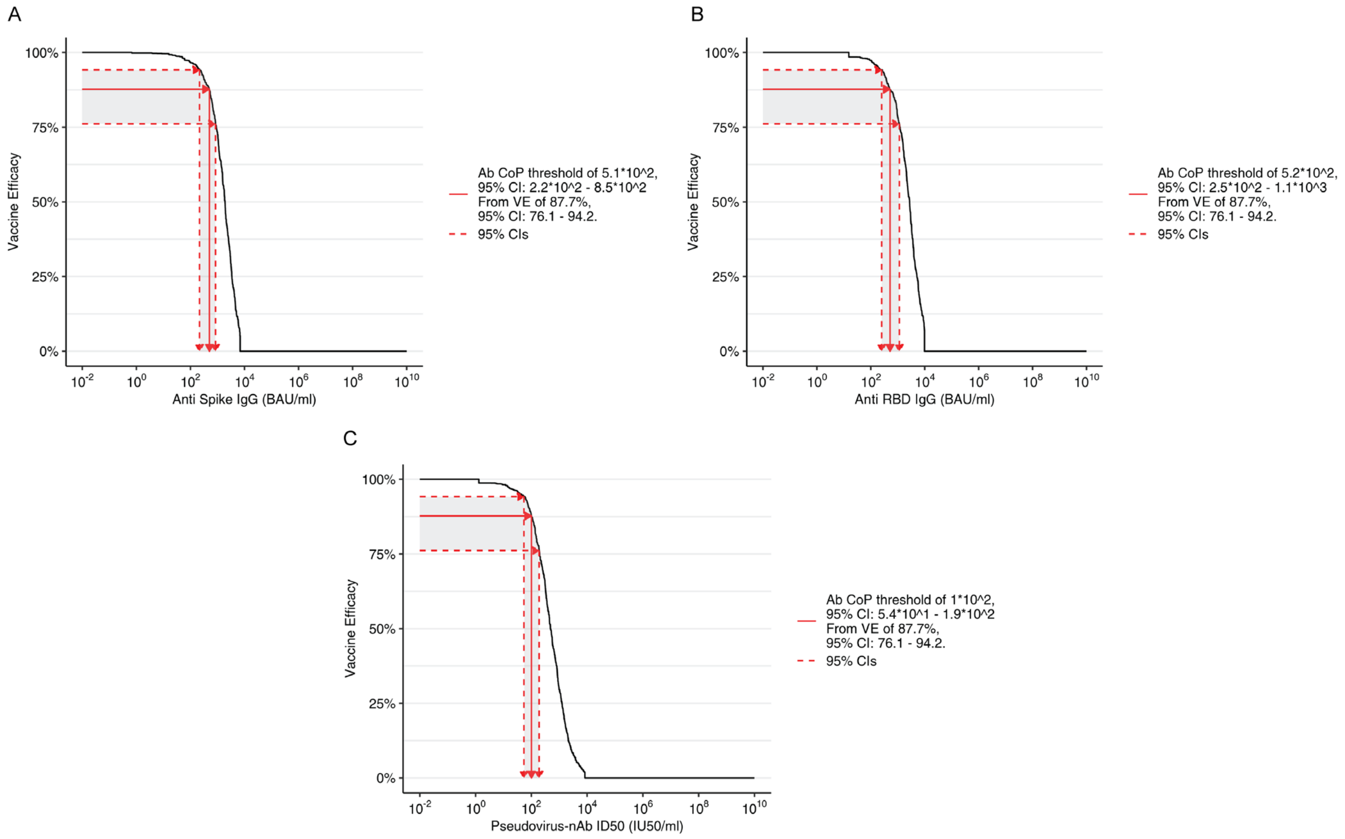

Supplementary Figure 3. Inverse probability sampling (IPS)-weighted empirical reverse cumulative distribution function curves for D35 (A) anti-spike IgG concentration, (B) anti-RBD IgG concentration, or (C) pseudovirus-nAb ID50 titer and application of the Siber (2007) method[2] for estimating a threshold of perfect vs. no protection.

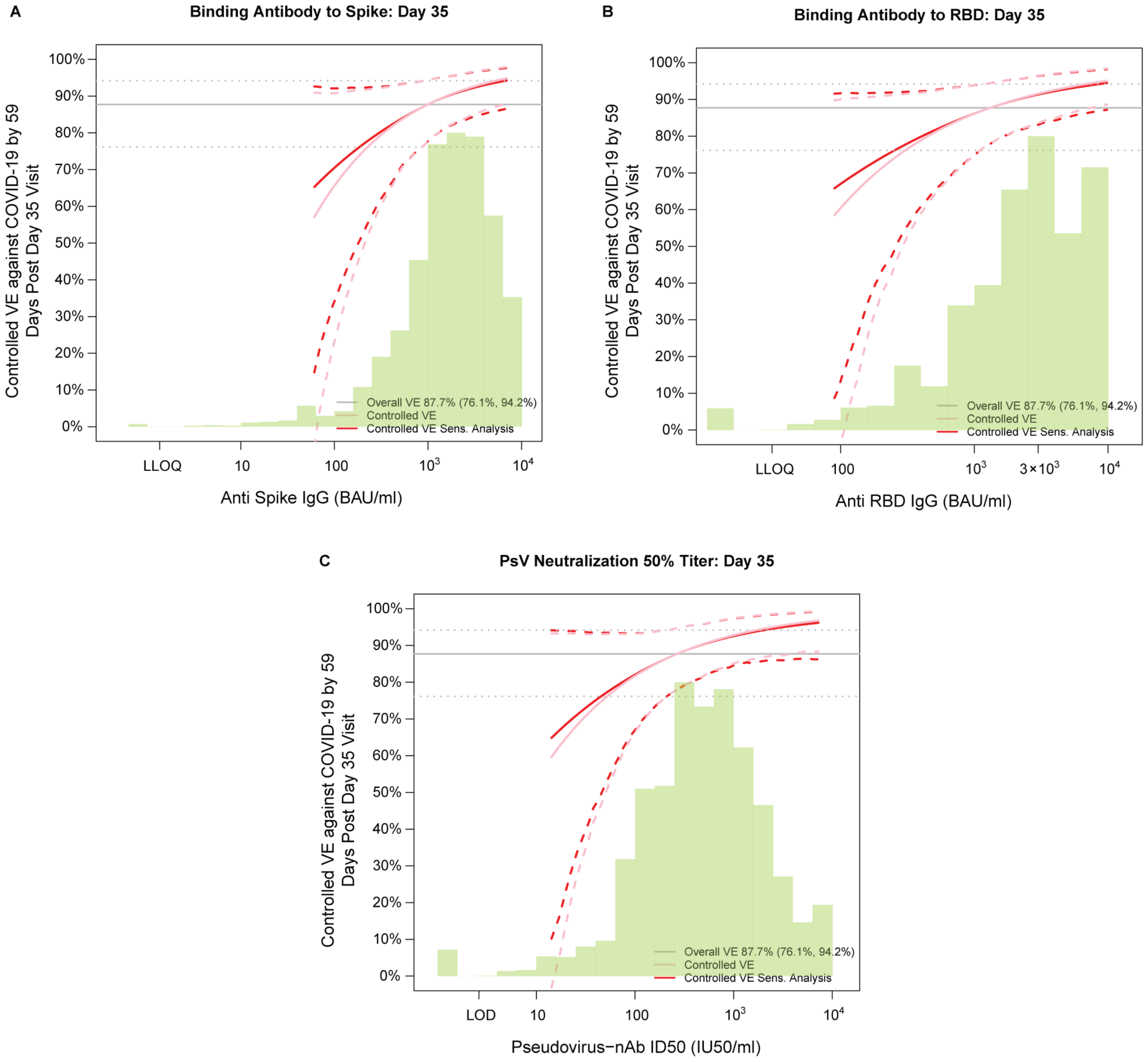

Supplementary Figure 4. Vaccine efficacy in the US study population with causal sensitivity analysis by Day 35 (A) anti-spike IgG concentration, (B) anti-RBD IgG concentration, or (C) pseudovirus (PsV)-nAb ID50 titer. Vaccine efficacy estimates were obtained using the method of Gilbert et al.[3] The green histogram is an estimate of the density of D35 antibody marker level and the horizontal gray line is the overall vaccine efficacy from 7 to 59 days post D35, with the dotted gray lines indicating the 95% confidence intervals (this number 87.7% differs from the 90.4% reported in ref.[1], which was based on counting COVID-19 endpoints starting 7 days post D35 in both the US and Mexico study sites). The pink solid line is point estimates assuming no unmeasured confounding; the dashed lines are bootstrap point-wise 95% CIs. The red solid line is point estimates assuming unmeasured confounding in a sensitivity analysis (dashed lines are bootstrap point-wise 95% CIs); see the Statistical Analysis Plan for details. LLOQ, lower limit of quantitation; LOD, (lower) limit of detection. Analyses adjusted for baseline risk score.

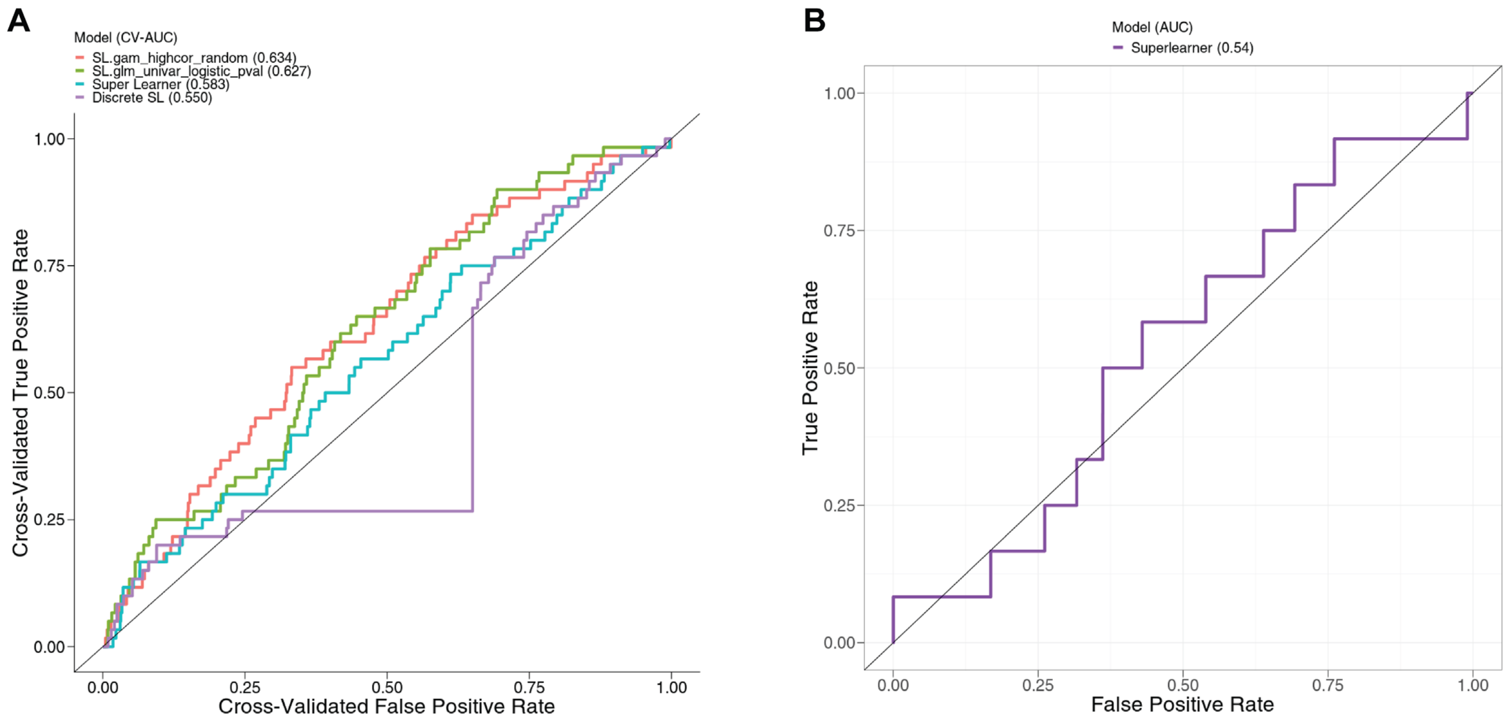

Supplementary Figure 5. Performance of the baseline risk score built from ensemble machine learning. (A) Receiver operating characteristic (ROC) curves based on cross-validated (CV)-estimated predicted probabilities for the top two learners, Superlearner and Discrete Superlearner. CV-estimated predicted probabilities were computed using only data from the placebo arm in the US with cases as COVID-19 endpoints starting post-enrollment. (B) ROC curve based on Superlearner predicted probabilities in vaccine recipients used in the correlates analyses with cases considered as COVID-19 endpoints starting 7 days post second vaccination visit and non-cases as participants with follow-up beyond 7 days post second vaccination visit who never registered a COVID-19 endpoint.

Supplementary Table 1. Sample sizes of baseline SARS-CoV-2 negative per-protocol vaccine recipients included in the case-cohort set included in immune correlates analyses (U.S. study sites), by baseline sampling strata and case/non-case strata.

Case-cohort set = Baseline SARS-CoV-2 negative per-protocol vaccine recipients included in D35 marker correlates analysis [in the immunogenicity subcohort (IS) and/or a breakthrough COVID-19 case)]*

	Baseline Sampling Strata of Baseline SARS-CoV-2 Negative Per-Protocol Vaccine Participants Included in Correlates Analyses								
	1	2	3	4	5	6	7	8	Total
Breakthrough COVID-19 cases (both within and outside the IS*) with D0, D35 Ab marker data	2	1	1	1	3	4	0	0	12
Non-cases in the IS with D0, D35 Ab marker data	102	97	89	80	74	73	55	69	639

Ab, antibody; IS, immunogenicity subcohort

Demographic covariate strata:

1. U.S. White Non-Hispanic**, age 18-64, No coexisting conditions***
2. U.S. White Non-Hispanic, age 18-64, Coexisting conditions
3. U.S. White Non-Hispanic, age ≥ 65, No coexisting conditions
4. U.S. White Non-Hispanic, age ≥ 65, Coexisting conditions
5. U.S. Minority, age 18-64, No coexisting conditions
6. U.S. Minority, age 18-64, Coexisting conditions
7. U.S. Minority, age ≥ 65, No coexisting conditions
8. U.S. Minority, age ≥ 65, Coexisting conditions

Cases are baseline SARS-CoV-2 negative per-protocol vaccine recipients with the primary COVID-19 endpoint starting 7 days post D35 visit through to the data cut (April 19, 2021).
Non-cases/Controls are baseline negative per-protocol vaccine recipients sampled into the immunogenicity subcohort with no evidence of SARS-CoV-2 infection up to the end of the correlates study period (the data cut-off date April 19, 2021). IS membership required availability of D0 and D35 antibody data and no evidence of SARS-CoV-2 infection through 6 days post D35.

*All breakthrough COVID-19 cases with D0, D35 Ab marker data were outside the IS

** White Non-Hispanic is defined as Race=White and Ethnicity=Not Hispanic or Latino. All other Race subgroups are defined as Black, Asian, American Indian or Alaska Native, Native Hawaiian or Other Pacific Islander, Multiracial, Other, Not reported, or Unknown.
Minority is defined as the complement of being known to be White Non-Hispanic.

***Coexisting conditions are the same as those listed in Table 1 of Dunkle et al.[1]: obesity (defined as a body-mass index [the weight in kilograms divided by the square of the height in meters] of ≥30.0), chronic lung disease, diabetes mellitus type 2, cardiovascular disease, or chronic kidney disease.

Supplementary Table 2. PREVENT-19 U.S. cohort demographic and clinical characteristics at enrollment in the baseline SARS-CoV-2 negative per-protocol immunogenicity subcohort*

Characteristics	Vaccine (N = 669*)	Placebo (N = 76)	Total (N = 745)
**Age**			
Age 18-64	355 (53.1%)	42 (55.3%)	397 (53.3%)
Age ≥ 65	314 (46.9%)	34 (44.7%)	348 (46.7%)
Mean (Range)	55.0 (18.0, 86.0)	54.6 (22.0, 80.0)	55.0 (18.0, 86.0)
**Coexisting Conditions****			
Yes	329 (49.2%)	41 (53.9%)	370 (49.7%)
No	340 (50.8%)	35 (46.1%)	375 (50.3%)
**Age, Coexisting Conditions**			
Age 18-64 Coexisting conditions	174 (26.0%)	22 (28.9%)	196 (26.3%)
Age 18-64 No coexisting conditions	181 (27.1%)	20 (26.3%)	201 (27.0%)
Age ≥ 65	314 (46.9%)	34 (44.7%)	348 (46.7%)
**Sex**			
Female	309 (46.2%)	39 (51.3%)	348 (46.7%)
Male	360 (53.8%)	37 (48.7%)	397 (53.3%)
**Hispanic or Latino Ethnicity**			
Hispanic or Latino	143 (21.4%)	12 (15.8%)	155 (20.8%)
Not Hispanic or Latino	521 (77.9%)	64 (84.2%)	585 (78.5%)
Not reported and unknown	5 (0.7%)	0 (0.0%)	5 (0.7%)
**Race**			
White	453 (67.7%)	47 (61.8%)	500 (67.1%)
Black or African American	127 (19.0%)	19 (25.0%)	146 (19.6%)
Asian	46 (6.9%)	6 (7.9%)	52 (7.0%)
American Indian or Alaska Native	18 (2.7%)	1 (1.3%)	19 (2.6%)
Native Hawaiian or Other Pacific Islander	1 (0.1%)	0 (0.0%)	1 (0.1%)
Multiracial	12 (1.8%)	1 (1.3%)	13 (1.7%)
Not reported and unknown	12 (1.8%)	2 (2.6%)	14 (1.9%)

*Of the 669 sampled vaccine recipients, 639 had antibody marker data measured at D0 and D35 and had no evidence of SARS-CoV-2 infection through 6 days post D35 and hence are in the immunogenicity subcohort.

** Coexisting conditions are the same as those listed in Table 1 of Dunkle et al.[1]: obesity (defined as a body-mass index [the weight in kilograms divided by the square of the height in meters] of ≥30.0), chronic lung disease, diabetes mellitus type 2, cardiovascular disease, or chronic kidney disease.

Supplementary Table 3. Distribution of variants among primary COVID-19 endpoints in PREVENT-19 starting 7 days post D35 through to the data cut-off (April 19, 2021) (no primary endpoints were with the ancestral/Wuhan-Hu-1 strain) with available sequence data.

Variant (PANGO lineage)*	CDC and Prevention Classification (June 2021)	Placebo (n=37)	Vaccine (n=7)
B.1	Wuhan Ancestral lineage	2	1
B.1.1	Wuhan Ancestral lineage	1	0
B.1.1.519**	Wuhan Ancestral lineage	1	0
B.1.1.7 (Alpha)	VoC	21	3
B.1.2	Wuhan Ancestral lineage	2	0
B.1.311	Wuhan Ancestral lineage	1	0
B.1.351 (Beta)	VoC	0	1
B.1.526 (Iota)	VoI	2	2
B.1.596	Wuhan Ancestral lineage	1	0
**B.1.617.1 (Kappa)**	VoI	1	0
**B.1.623**	Wuhan Ancestral lineage	1	0
**B.1.637**	Wuhan Ancestral lineage	1	0
**P.1 (Gamma)**	VoC	2	0
**P.2 (Zeta)**	VoI	1	0

*Variants without a Greek letter are of the Wuhan Ancestral lineage and have never been classified by the Center for Disease Control and Prevention as a variant of interest (VoI) or as a variant of concern (VoC).
**Formerly monitored variant never classified as a VoI or VoC.

Supplementary Table 4. D35 antibody marker response rates and geometric means in the U.S. cohort by COVID-19 outcome status. Analysis based on baseline SARS-CoV-2 negative per-protocol placebo recipients in the case-cohort set. Median (interquartile range) days from vaccination to D35 was 38 (5).

		Placebo COVID-19 Cases[1]			Placebo Non-Cases in Immunogenicity Subcohort[2]		Comparison	
D35 Marker	N	Proportion with Antibody Response[3] (95% CI)	Geometric Mean (GM) (95% CI)	N	Proportion with Antibody Response[3] (95% CI)	Geometric Mean (GM) (95 % CI)	Response Rate Difference (Non-Cases – Cases)	Ratio of GM (Non-Cases/ Cases)
Anti Spike IgG (BAU/ml)	41	7.3% (2.3%, 21.0%)	1.19 (0.89, 1.59)	72	0.9% (0.2%, 3.5%)	0.82 (0.70, 0.95)	0.2% (0%, 1.7%)	0.68 (0.50, 0.95)
Anti RBD IgG (BAU/ml)	41	0.0% (0.0%, 0.0%)	15.32 (15.32, 15.32)	72	0.2% (0.0%, 1.7%)	15.51 (15.15, 15.87)	0.2% (0%, 1.7%)	1.01 (0.99, 1.03)
Pseudovirus-nAb ID50 (IU50/ml)	41	0.0% (0.0%, 0.0%)	1.31 (1.31, 1.31)	72	0.2% (0.0%, 1.7%)	1.32 (1.29, 1.35)	-6.4% (-20.1%, -0.8%)	1.01 (0.99, 1.03)

[1]Cases are baseline SARS-CoV-2 negative per-protocol placebo recipients with the primary COVID-19 endpoint (symptomatic RT-PCR-confirmed COVID-19) starting 7 days post D35 visit through to the efficacy data cut-off date (April 19, 2021).

[2]Non-cases are are baseline negative per-protocol placebo recipients sampled into the immunogenicity subcohort with no evidence of SARS-CoV-2 infection up to the end of the correlates study period (the data cut-off date April 19, 2021).

[3]Antibody response defined by Spike IgG or RBD IgG concentration above the antigen-specific positivity cut-off (10.8424 BAU/ml and 30.6 BAU/ml, respectively) or by detectable ID50 > limit of detection (LOD) = 2.612 IU50/ml.

Supplementary Table 5. Assay limits of the two antibody markers evaluated as immune correlates. BAU = binding antibody units; IU = International Units; LLOQ, lower limit of quantitation; ULOQ, upper limit of quantitation.

MSD Binding Assay (Nexelis) (Spike and RBD IgG markers)		
Reported units	BAU/ml	
	Spike	RBD
Positivity Cutoff	10.8424	30.6
LLOQ	1.35	30.6
ULOQ	6934	9801
All values < LLOQ were set to LLOQ/2		
All values > ULOQ were set to ULOQ (for immune correlates analyses)		

Pseudovirus neutralization titer (Monogram) (nAb ID50 marker)	
Reported units	IU50/ml
LOD*	2.612
LLOQ	3.3303
ULOQ	8319.938
Values < LOD are denoted as undetectable responses and were set to LOD/2	
All values > ULOQ were set to ULOQ (for immune correlates analyses)	

*The limit of detection (LOD) was not formally defined; we denote the value corresponding to the starting dilution level of the assay as the LOD.

Supplementary Table 6. Individual baseline variables in baseline SARS-CoV-2 negative per-protocol placebo recipients that were used in the ensemble machine learning for developing risk score and predicting occurrence of COVID-19.

Variable Name	Definition	Total missing values*
Age	Age at enrollment in years	0/7332 (0.0%)
Sex	Sex assigned at birth (1=female, 0=male)	0/7332 (0.0%)
Black	Indicator race = Black (0 = White)	0/7332 (0.0%)
Asian	Indicator race = Asian (0 = White)	0/7332 (0.0%)
Ethnicity Hispanic	Indicator ethnicity = Hispanic or Latino (0 = Non-Hispanic/Non-Latino)	0/7332 (0.0%)
Height	Height at baseline (cm)	23/7332 (0.3%)
Weight	Weight at baseline (kg)	24/7332 (0.3%)
BMI	BMI at enrollment (kg/m^2)	24/7332 (0.3%)
HighRiskInd	Protocol-defined high-risk pre-existing condition (1=yes, 0=no)	0/7332 (0.0%)

*Missing values in variables Height, Weight, and BMI were imputed using the mice package in R.

Supplementary Table 7. Ensemble (Superlearner) model with individual learners sorted by weight. Predictors within each learner are sorted by variable importance which is the absolute value in Coefficient (in case of learners SL.glm, SL.glmnet, and SL.gam), or Importance (in case of SL.ranger).

Learner	Screen	Weight	Predictors	Coefficient	Odds Ratio	Importance
SL.glm	Univariate logistic p-value	0.431	(Intercept)	-4.93	0.007	N/A
			Age	-0.438	0.645	N/A
			Sex	0.293	1.34	N/A
SL.glmnet	No screen	0.304	(Intercept)	-4.885	0.008	N/A
			Age	-0.365	0.694	N/A
			Sex	0.212	1.236	N/A
			Asian	0.056	1.058	N/A
			HighRiskInd	0.113	1.119	N/A
SL.gam	High-correlation	0.221	(Intercept)	-4.959	0.007	N/A
			s(Age, 2)	-0.461	0.631	N/A
			s(Height, 2)	0.598	1.818	N/A
			s(Weight, 2)	-0.842	0.431	N/A
			s(BMI, 2)	0.804	2.235	N/A
			Sex	0.454	1.574	N/A
			Black	-0.016	0.984	N/A
			Asian	0.13	1.139	N/A
			EthnicityHispanic	-0.057	0.945	N/A
			HighRiskInd	0.179	1.196	N/A
SL.ranger	No screen	0.044	BMI	NA	NA	33.275
			Weight	NA	NA	26.939
			Height	NA	NA	24.056
			Age	NA	NA	15.654
			Sex	NA	NA	2.386
			EthnicityHispanic	NA	NA	1.954
			Black	NA	NA	1.642
			HighRiskInd	NA	NA	1.533
			Asian	NA	NA	1.29

Supplementary Table 8. Algorithms included in Ensemble machine learning for baseline risk score development.

Algorithms	Screens*/Tuning Parameters
SL.mean	No screen
SL.glm	No screen, Glmnet, Univariate logistic p-value, High-correlation
SL.glm.interaction	Glmnet, Univariate logistic p-value, High-correlation
SL.glmnet	No screen
SL.gam	Glmnet, Univariate logistic p-value, High-correlation
SL.xgboost	No screen
SL.ranger	No screen

***Screen:** Pre-selection of variables to be fed to the individual learners based on statistical criteria.
Glmnet: only includes variables with non-zero coefficients in a lasso model with default tuning parameter selection.
Univariate logistic p-value: only includes variables with logistic regression univariate Wald test 2-sided p-value < 0.1.
High-correlation: selects variables at random from amongst a pair with high correlation (Spearman rank correlation > 0.9).

Supplementary References

1. Dunkle LM, Kotloff KL, Gay CL, et al. Efficacy and Safety of NVX-CoV2373 in Adults in the United States and Mexico. *N Engl J Med* 2022; **386**(6): 531-43.

2. Siber GR, Chang I, Baker S, et al. Estimating the protective concentration of anti-pneumococcal capsular polysaccharide antibodies. *Vaccine* 2007; **25**(19): 3816-26.

3. Gilbert PB, Fong Y, Kenny A, Carone M. A Controlled Effects Approach to Assessing Immune Correlates of Protection. kxac024, https://doi.org/10.1093/biostatistics/kxac24. *Biostatistics* 2022.

Statistical Analysis Plan for Assessing Immune Correlates in the Coronavirus Efficacy (PREVENT-19) Phase 3 Trial of the NVX-CoV2373 COVID-19 Vaccine

USG COVID-19 Response Team / Coronavirus Prevention Network (CoVPN) Biostatistics Team

Peter B. Gilbert[1,2*], Youyi Fong[1,2], David Benkeser[3], Jessica Andriesen[1], Bhavesh Borate[1], Marco Carone[2], Lindsay N. Carpp[1], Iván Díaz[4], Michael P. Fay[5], Andrew Fiore-Gartland[1], Nima S. Hejazi[6], Ying Huang[1,2], Yunda Huang[1], Ollivier Hyrien[1], Holly E. Janes[1,2], Michal Juraska[1], Alex Luedtke[7], Martha Nason[5], April K. Randhawa[1], Lars van der Laan[6], Brian D. Williamson[1], Dean Follmann[5]

[1]Vaccine and Infectious Disease and Public Health Sciences Divisions, Fred Hutchinson Cancer Research Center, Seattle, Washington

[2]Department of Biostatistics, University of Washington, Seattle, Washington

[3]Department of Biostatistics and Bioinformatics, Rollins School of Public Health, Emory University, Atlanta, Georgia

[4]Department of Population Health Sciences, Weill Cornell Medical College, New York, New York

[5]National Institute of Allergy and Infectious Diseases, Bethesda, Maryland

[6]Division of Biostatistics, School of Public Health, University of California, Berkeley, California

[7]Department of Statistics, University of Washington, Seattle, Washington

Correspondence: *pgilbert@fredhutch.org

June 22, 2022

Contents

List of Tables 4

List of Figures 5

1 Introduction 6

2 Antibody Assays and and Day 35 Markers 7

3 Study Cohorts and Endpoints 10
3.1 Study Cohort for Correlates Analyses 10
3.2 Study Endpoints 11

4 Objectives of Immune Correlates Analyses of a Phase 3 Trial Data Set 11
4.1 Correlates of Risk and Correlates of Protection 11

5 Case-cohort Sampling Design for Measuring Antibody Markers 13
5.1 Immunogenicity subcohort 14

6 Unsupervised Feature Engineering of Antibody Markers (Stage 1: Day 1, 35) 15
6.1 Descriptive Tables and Graphics 15
6.1.1 Antibody marker data 15
6.1.2 Graphical description of antibody marker data 20
6.2 Methods for Positive Response Calls for bAb and nAb Assays 22
6.3 SARS-CoV-2 Antigen Targets Used for bAb and nAb Markers 22

7 Baseline Risk Score (Proxy for SARS-CoV-2 Exposure) 22

8 Correlates Analysis Descriptive Tables by Case/Non-Case Status 24

9 Correlates of Risk Analysis Plan 25

9.1 CoR Objectives 25
9.2 Outline of the Set of CoR Analyses 25
9.3 Day 35 Markers Assessed as CoRs and CoPs 25
9.3.1 Inverse probability sampling weights used in CoR analyses 26
9.3.2 Choice of regression methods 27
9.3.3 Univariate CoR: Nonparametric threshold regression modeling 29
9.3.4 P-values and Multiple hypothesis testing adjustment for CoR analysis 31
9.4 Implementation of superlearner for baseline risk score development 32

10 Correlates of Protection: Generalities 35

11 Correlates of Protection: Interventional Effects 36
11.1 CoP: Controlled Vaccine Efficacy 36
11.1.1 Point and 95% confidence interval estimation of CVE(s) and of $RR_C(s_1, s_2) = (1 - \text{CVE}(s_2))/(1 - \text{CVE}(s_1))$ assuming the causal assumptions hold 37
11.1.2 Sensitivity analysis (to unmeasured confounding) for the Cox model controlled vaccine efficacy analysis . . . 39

12 Estimating a Threshold of Protection Based on an Established or Putative CoP (Population-Based CoP) 45

13 Considerations for Baseline SARS-CoV-2 Positive Study Participants 46

14 Avoiding Bias with Pseudovirus Neutralization Analysis due to Use of Anti-HIV Antiretroviral Drugs 47

15 Novavax Binary Principal Stratification Results 55

List of Tables

1 Correlates of Risk (CoRs) and Correlates of Protection (CoPs) Objectives for Day 35 Markers 13
2 Baseline Subgroups that are Analyzed[1]. 19
3 Learning Algorithms in the Superlearner Library of Estimators of the Conditional Probability of Outcome, for Building the Baseline Risk Score Based on the Placebo Arm[1]. 34
4 Learning Algorithms in the Superlearner Library of Estimators of the Conditional Probability of Outcome: Simplified Library in the Event of Fewer than 50 Placebo Arm Cases for an Analysis, for Building a Baseline Behavioral Risk Score in Novavax PREVENT-19[1]. 34
5 Novavax PREVENT-19: Correlates of Vaccine Efficacy Results by Gilbert et al. (2020) Method for High vs. Low Marker Subgroups Under No Early Harm Assumption with Sensitivity Analysis Scenarios* 57
6 Novavax PREVENT-19: Correlates of Vaccine Efficacy Results by Gilbert et al. (2020) Method for High vs. Low Marker Subgroups Under No Early Harm Assumption with Sensitivity Analysis Scenarios* 58
7 Novavax PREVENT-19: Cut-points Defining High and Low Marker Subgroups 59
8 Novavax PREVENT-19: Cut-points Defining High and Low Marker Subgroups 59
9 Novavax PREVENT-19: Cut-points Defining High and Low Marker Subgroups 59

List of Figures

1 Planned Immunogenicity Subcohort Sample Sizes by Baseline Strata for Antibody Marker Measurement 15
2 A) Structural relationships among study endpoints in a COVID-19 vaccine efficacy trial (Mehrotra et al., 2020). B) Study endpoint definitions. 48
3 Example at-COVID diagnosis and post-COVID diagnosis disease severity and virologic sampling schedule, in a setting where frequent follow-up of confirmed cases can be assured. Participants diagnosed with virologically-confirmed symptomatic SARS-CoV-2 infection (COVID) enter a post-diagnosis sampling schedule to monitor viral load and COVID-related symptoms (types, severity levels, and durations). 49
4 Case-cohort sampling design (Prentice, 1986) that measures Day 1, Day 35 antibody markers in all participants selected into the subcohort and in all COVID and COV-INF cases occurring outside of the subcohort. 50
5 Two-stage correlates analysis. Stage 1 consists of analyses of Day 35 markers as correlates of risk and of protection of the primary endpoint and potentially also of some secondary endpoints, and includes antibody marker data from all COVID and SARS-CoV-2 infection cases (COV-INF) through to the time of the data lock for the first correlates analyses. Stage 2 consists of analyses of Day 35 markers as correlates of risk and of protection of longer term endpoints and analyses of longitudinal markers as outcome-proximal correlates of risk and of protection, and includes antibody marker data from all subsequent COVID and COV-INF cases. Stage 1 measures Day 1, Day 35 antibody markers and COV-INF and COVID diagnosis time point markers; Stage 2 measures antibody markers from all sampling time points and COV-INF plus COVID diagnosis sampling time points not yet assayed. The same immunogenicity subcohort is used for both stages. 51

1 Introduction

This SAP describes the statistical analysis of antibody markers measured at Day 35 as immune correlates of risk and as immune correlates of protection against the COVID primary endpoint in the Coronavirus Efficacy (PREVENT-19) phase 3 trial of the NVX-CoV2373 COVID-19 vaccine. In this trial, estimated efficacy of the NVX-CoV2373 vaccine against symptomatic COVID illness was 90.4% (95% confidence interval, 82.9 to 94.6%) [?]. Some key facts about this trial:

- There are sister trials in UK and South Africa; this SAP restricts to data from the US and Mexico phase 3 trial
- Two doses regimen at Day 0 and 21. Primary efficacy analysis starts at 7 days post dose 2 (second dose at Day 21). Markers measured at Day 35.
- Enrollment occurred between December 27, 2020, and February 18, 2021. Final analysis cutoff date is April 19, 2021.
- Per protocol means receiving two doses, baseline negative (6.5% baseline positive, where positive means either serology or nucleic acid positive), and being at risk at Day 28. Full analysis set means receiving one dose. 2:1 randomization ratio
- In PP population, a total of 77 cases (mild, moderate or severe) are observed, 14 in the vaccine arm and 63 in the placebo arm (Dunkle et al.).
- 31 of 77 cases are Alpha.
- COVID is classified into mild, moderate and severe. Mild requires at least two from a list of symptoms. Mild and moderate in PREVENT-19 roughly correspond to non-severe in COVE, which requires two symptoms, and moderate but not mild in ENSEMBLE, which require two and one symptom, respectively.
- Study include 6 sites in Mexico and 113 sites in US. In the primary analysis, all vaccine cases are in US and one placebo case is in Mexico.

For correlates all cases are in the US such that only US sites are included.

2 Antibody Assays and and Day 35 Markers

The antibody markers of interest are measured using two different humoral immunogenicity assays [more detail on assay type (2) can be found in Sholukh et al. (2020)]:

(1) **bAbs**: **Binding antibodies** to the vaccine insert SARS-CoV-2 proteins;

and (2) **Pseudovirus-nAbs**: **Neutralizing antibodies** against viruses **pseudotyped** with the vaccine insert SARS-CoV-2 proteins.

The Supplementary text in the article provides details of the assays. We include the necessary statistical details below.

(1) **bAb assay**: The MSD-ECL Multiplex Assay (MSD-ECL = meso scale discovery-electrochemiluminescence assay).

The MSD assay measures binding antibody to antigens corresponding to: Spike (an engineered version of the Spike protein harboring a double proline substitution (S-2P) that stabilizes it in the closed, prefusion conformation [McCallum et al. (2020)]); the Receptor Binding Domain (RBD) of the Spike protein; and Nucleocapsid protein (N), which is not contained in any of the COVID-19 vaccines.

The bAb assay readouts are in units AU/ml, where AU stands for arbitrary units from a standard curve. The process of validating the assay defined a lower limit of detection (LOD), an upper limit of detection (ULOD), a lower limit of quantitation (LLOQ), an upper limit of quantitation (ULOQ), and a positivity cut-off for each antigen that defines positive vs. negative response. These values are as follows:

- bAb Spike:
  - Pos. Cutoff = 1204.711 AU/ml
  - LLOQ = 150.4 AU/ml
  - ULOQ = 770,464.6 AU/ml

– LOD = N/A

– ULOD = N/A

The Vaccine Research Center established factors for bridging the MSD assay readouts from AU/ml to Binding Antibody Units/ml (BAU/ml), based on bridging to the WHO International Standard for anti-SARS-CoV-2 immunoglobulin. For the three binding antibody variables CoV-2 Spike IgG, CoV-2 RBD IgG, and CoV-2 N IgG, these conversion factors are 0.0090, 0.0272, and 0.0024, respectively. These conversion factors are applied, such that all binding Ab readouts are reported in BAU/ml, for all analyses. These conversion factors are also applied to yield the LOD, ULOD, LLOQ, and ULOQ on the WHO IU/ml scale. The following shows the assay limits on the BAU/ml scale:

- bAb Spike:
  - Pos. Cutoff = 10.8424 BAU/ml
  - LOD = 0.3076 BAU/ml
  - ULOD = 172,226.2 BAU/ml
  - LLOQ = 1.35 BAU/ml
  - ULOQ = 6934 BAU/ml

All values below the LLOQ are assigned the value LLOQ/2. A positive response is defined by value above the Pos. Cutoff. For immunogenicity reporting, values greater than the ULOQ are not given a ceiling value of the ULOQ, the actual readouts are used. For the immune correlates analyses, values greater than the ULOQ are assigned the value of the ULOQ.

(2) **Pseudovirus-nAb assay**: A firefly luciferase (ffLuc) reporter neutralization assay for measuring neutralizing antibodies against SARS-CoV-2 Spike-pseudotyped viruses.

Based on the assay in the Monogram lab, serum inhibitory dilution 50% titer (ID50) values are estimated based on a starting serum dilution of 1:40, with a total of ten 3-fold dilutions. (Each sample is diluted initially at 1:20, then

diluted serially 3-fold for a total of 10 concentrations. The starting dilution of 1:20 is reported as 1:40 after addition of the virus.) So, the dilution series is 1:40 to 1:787,320 (= 40 * 39). Thus 1:40 is the LOD on the scale of the assay. The process of validating the assay defined the LOD, LLOQ, and ULOQ for ID50 as follows:

- ID50:
  - LOD = 40
  - LLOQ = 51
  - ULOQ = 127411

ID50 values below the LOD are assigned the value LOD/2 = 40/2 = 20. For immunogenicity reporting, values greater than the ULOQ are not given a ceiling value of the ULOQ, the actual readouts are used. For the immune correlates analyses, values greater than the ULOQ are assigned the value of the ULOQ.

ID50 values are reported in international units with the following calibration factor, defined using the D614G strain in the assay:

- Calibration factor ID50: 0.0653

The original readouts are calibrated to the IU scale by multiplying each original ID50 value by 0.0653 (See Feng et al. Table 2 and Gilbert et al. Supplementary Material), and units are reported in international units as IU50/ml for ID50. Consequently, the LOD, LLOQ and ULOQ for IU50/ml are as follows in International Units:

- IU50/ml:
  - LOD = 2.612
  - LLOQ = 3.3303
  - ULOQ = 8319.938

Therefore the lowest possible value of ID50 readouts on the log10 scale is log10(2.612/2) = 0.116. Positivity cutoff is assigned LOD.

Based on each immunoassay applied to serum samples collected from participants on Day 1 (baseline, first dose of vaccination visit)and Day 35 (post-vaccination visit), the following set of antibody markers was defined for immunogenicity and immune correlates analyses.

- For bAb: $\log_{10}$ IgG concentration (BAU/ml) at each time point, and the difference in $\log_{10}$ concentration (Day 35 minus Day 1) representing $\log_{10}$ fold-rise in IgG concentration from baseline to 14 days post dose two. These markers are defined for Spike.
- For PsV nAb: $\log_{10}$ serum inhibitory dilution 50% titer (ID50 in IU50/ml) at each time point, as well as the $\log_{10}$ fold-rise of these markers over Day 1 to Day 35.

3 Study Cohorts and Endpoints

3.1 Study Cohort for Correlates Analyses

The analysis cohort for the correlates analysis is baseline SARS-CoV-2 negative participants in the per-protocol cohort, with the per-protocol cohort defined as those who received both planned vaccinations without any specified protocol deviations, and who were SARS-CoV-2 negative at the terminal vaccination visit. We refer to this cohort representing the primary population for correlates analysis as the Per-Protocol Baseline Negative Cohort.

As the primary analysis of vaccine efficacy is conducted in baseline negative individuals, correlates of risk (CoR) and correlates of protection (CoP) analyses are only done in baseline negative individuals, and the analysis of data from baseline positive individuals is for purposes of immunogenicity characterization, given too-few anticipated vaccine breakthrough study endpoints for CoR/CoP assessment (although if there are many baseline positive vaccine breakthrough endpoint cases that baseline positive subgroup analyses may be considered). In baseline negative individuals, antibody marker data in placebo recipients is relevant for verifying the expectation that almost all Day 35 marker responses will be negative, given the lack of SARS-CoV-2 antigen exposure.

3.2 Study Endpoints

Endpoints for correlates analyses of Day 35 markers are included if they occur at least 7 days after the Day 35 visit, to help ensure that the endpoint did not occur prior to Day 35 antibody measurement.

Figure 2 defines five study endpoints assessed in COVID-19 vaccine efficacy trials, where COVID (symptomatic infection) is used as the primary endpoint in the PREVENT-19 trial. Only the COVID endpoint is assessed in the current manuscript. For the correlates analysis, all available follow-up for participants is included through to the time of the data base lock for the correlates analysis, for every CoR and CoP analysis that is conducted. This means that the time of right censoring for a given failure time endpoint is the first event of loss to follow-up or the date of administrative censoring defined as the last date of available follow-up. For CoP analyses, which use both vaccine and placebo recipient data and leverage the randomization, follow-up is censored at the time of unblinding. In general for the current manuscript all blinded follow-up is included and no post-unblinding follow-up is included.

4 Objectives of Immune Correlates Analyses of a Phase 3 Trial Data Set

4.1 Correlates of Risk and Correlates of Protection

We broadly classify the proposed analyses into two related categories: correlates of risk (CoR) and correlates of protection (CoP) analyses. CoR analyses seek to characterize correlations/associations of markers with future risk of the outcome amongst vaccinated individuals in the study cohort. CoP analyses seek to formally characterize causal relationships among vaccination, antibody markers and the study endpoint, and use data from both vaccine and placebo recipients. Table 1 summarizes these objectives and statistical frameworks that are commonly used to these ends.

The advantage of CoR analyses it that it is possible to obtain definitive answers from the phase 3 data sets, that is one can credibly characterize associations between markers and outcome. The advantage of CoP analyses is

that the effects being estimated have interpretation directly in terms of how an antibody marker can be used to reliably predict vaccine efficacy (the criterion for use of a non-validated surrogate endpoint for accelerated approval, Fleming and Powers, 2012). The disadvantage of CoR analyses are that a CoR may fail to be a CoP, for example due to unmeasured confounding, lack of transitivity where a vaccine effect on an antibody marker occurs in different individuals than clinical vaccine efficacy, or off-target effects (VanderWeele, 2013). The disadvantage of CoP analyses is that statistical inferences rely on causal assumptions that cannot be completely verified from the phase 3 data, such that compelling evidence may require multiple phase 3 trials and external evidence on mechanism of protection (e.g., from adoptive transfer or vaccine challenge trials). Our approach presents results for both CoR and CoP analyses, seeking clear exposition of how to interpret results, the assumptions undergirding the validity of the results, and diagnostics of these assumptions and assessment of robustness of findings to violation of assumptions.

Table 1: Correlates of Risk (CoRs) and Correlates of Protection (CoPs) Objectives for Day 35 Markers

**Objective Type**	**Objective**
**CoRs (Risk Prediction Modeling)**	**To assess Day 35 markers as CoRs in vaccine recipients**<br>a. Relative risks of outcome across marker levels<br>b. Absolute risk of outcome across marker levels<br>c. Machine learning risk prediction for multivariable markers
**CoP: Correlates of VE**	**To assess Day 35 markers as correlates of VE in vaccine recipients**<br>a. Principal stratification effect modification analysis<br>b. Assesses VE across subgroups of vaccine recipients defined by Day 35 marker level in vaccine recipients
**CoP: Controlled Effects on Risk and VE**	**To assess Day 35 markers for how assignment to vaccine and a fixed marker value would alter risk compared to assignment to placebo**
**CoP: Stochastic Interventional Effects on Risk and VE**	**To assess Day 35 markers for how stochastic shifts in their distribution would alter mean risk and VE** (Hejazi et al., 2020)
**CoP: Mediators of VE**	**To assess Day 35 markers as mediators of VE**<br>a. Mechanisms of protection via natural direct and indirect effects<br>a. Estimate the proportion of VE mediated by a marker or markers

Because there are only 12 vaccine breakthrough COVID-19 endpoints with D35 antibody data available for correlates analyses, only a subset of the statistical methods were applied. In particular only the CoR analyses a. and b., the CoR nonparametric threshold analyses, and the CoP Controlled Effects on Risk and VE through marginalized Cox modeling are applied.

5 Case-cohort Sampling Design for Measuring Antibody Markers

Figure 4 illustrates the case-cohort (Prentice, 1986) sampling design that is used for measuring Day 1, 35 antibody markers in a random sample of trial participants. The random sample is stratified by the key baseline covariates: assigned randomization arm, baseline SARS-CoV-2 status (negative means negative in both the anti-NP binding antibody assay and the nasal swab RT-

PCR assay; positive means positive in either assay, Dunkle et al., 2021), and demographics strata (8 US strata formed by Age 18-64 or $\geq$ 65, Underrepresented Minority: Yes vs. No /Unknown, and Coexisting conditions: Yes vs. No, and 2 Mexico strata, Age 18-64 or $\geq$ 65). Because the design uses a stratified random sample instead of the simple random sample proposed by Prentice (1986), the design may also be referred to as a "two-phase sampling design" (Breslow et al., 2009b,a), where "phase one" refers to variables measured in all participants and "phase two" refers to variables only measured in a subset (thus the "case-cohort sample" constitutes the phase-two data).

The case-cohort design enables obtaining marker data (Day 1, 35) for the immunogenicity subcohort during early trial follow-up in real-time batches, thereby accelerating the time until final data set creation and hence data analysis and results on Day 35 marker correlates. The design allows using the same immunogenicity subcohort to assess correlates for multiple endpoints, relevant for the COVID-19 VE trials with multiple endpoints (Figure 2). This makes the design operationally simpler than a case-control sampling design.

5.1 Immunogenicity subcohort

The immunogenicity subcohort was sampled from the subset of participants in the Full Analysis Set (FAS) cohort used in the primary analysis of vaccine efficacy against the primary endpoint (with the FAS defined as all randomized participants who received at least one dose of investigational product) for whom all of the following information was available: baseline SARS-CoV-2 status; age, race/ethnicity (needed to define Minority status as described below), and presence of coexisting conditions associated with high risk of severe COVID-19; and Day 1 and Day 35 samples collected.

Figure 1 summarizes the planned size of the immunogenicity subcohort, by the baseline factors used to stratify the random sampling. The subcohort sampling is implemented to create representative sampling across the entire period of enrollment.

For the sampling, Minority includes Blacks or African Americans, Hispanics or Latinos, American Indians or Alaska Natives, Native Hawaiians, and other

	Numbers of Participants Sampled Into **40** Strata (Total N=**1620**)			
Baseline SARS-CoV-2 Status[1]	Negative		Positive	
Covariate Strata[2]	1-8 (US)	9-10 (Mex)	11-18 (US)	19-20 (Mex)
Vaccine	100 each	50 each	35 each	13 each
Placebo	12 each	6 each	35 each	13 each

[1] Baseline SARS-CoV-2 status as defined in the SAP, i.e., negative means negative in both the anti-NP binding antibody assay and the nasal swab RT-PCR assay; positive means positive in either assay. Missing values from either assay are assigned negative, to be consistent with the primary efficacy analysis.

[2] Strata 1-20 subdivide the vaccine arm and strata 21-40 subdivide the placebo arm. Take the vaccine arm as example. Strata 1-10 subdivide the baseline negative population. They include 8 US strata formed by dividing the baseline negative according to (Age 18-64 or >= 65), (Underrepresented Minority: Yes vs. No /Unknown), and (Comorbidity: Yes vs. No), and 2 Mexico strata, (Age 18-64 or >= 65). Strata 11-20 correspondingly subdivide the baseline positive population.

Figure 1: Planned Immunogenicity Subcohort Sample Sizes by Baseline Strata for Antibody Marker Measurement

Pacific Islanders. Non-Minority includes all other races with observed race (Asian, Multiracial, White, Other) and observed ethnicity Not Hispanic or Latino. Therefore Unknown and Not reported have missing values for this sampling stratum variable.

Coexisting conditions refers to participants having coexisting conditions associated with high risk of severe COVID-19 illness, with co-existing conditions defined in Dunkle et al. (2021). (obesity, chronic lung disease, diabetes mellitus type 2, cardiovascular disease, and/or chronic kidney disease)

6 Unsupervised Feature Engineering of Antibody Markers (Stage 1: Day 1, 35)

6.1 Descriptive Tables and Graphics

6.1.1 Antibody marker data

Binding antibody titers to full length SARS-CoV-2 Spike protein is measured in all participants in the immunogenicity subcohort (augmented with COVID-19 endpoint cases). Binding antibody IgG Spike, as well as fold-

rise in this marker from baseline, are measured at each pre-defined time point. Indicators of 2-fold rise and 4-fold rise in IgG concentration (fold rise [post/pre] $\geq$ 2 and $\geq$ 4, 2FR and 4FR) are measured at each pre-defined post-vaccination timepoint. Binding antibody responders to a given antigen at each pre-defined timepoint are defined as participants with value above the antigen-specific positivity cut-off. Binding antibody IgG 2FR (4FR) at each pre-defined timepoint to a given antigen are defined as participants who had baseline values below the LLOQ with IgG concentration at least 2 times (4 times) above the assay LLOQ, or as participants with baseline values above the LLOQ with at least a 2-fold (4-fold) increase in IgG concentration.

Pseudovirus neutralizing antibody ID50 titers, as well as fold-rise in ID50 titers from baseline, are measured at each pre-defined time point. Indicators of 2-fold rise and 4-fold rise in ID50 titer (fold rise [post/pre] $\geq$ 2 and $\geq$ 4, 2FR and 4FR) are measured at each pre-defined post-vaccination timepoint. Neutralization responders at each pre-defined timepoint are defined as participants who had baseline values below the LOD with detectable ID50 neutralization titer above the assay LOD, or as participants with baseline values above the LOD with a 4-fold increase in neutralizing antibody titer. Neutralization 2FR (4FR) at each pre-defined timepoint are defined as participants who had baseline values below the LLOQ with ID50 at least 2 times (4 times) above the assay LLOQ, or as participants with baseline values above the LLOQ with at least a 2-fold (4-fold) increase in neutralizing antibody titer.

Note that for defining positive response, 2FR, and 4FR, a reason why values below the LOD are set to half the LOD before calculating the indicator of response, is to ensure that a vaccine recipient that has an unusually low antibody readout at baseline and a post-vaccination value below or near the LOD is not erroneously counted as a responder.

The following list describes the antibody variables that are measured from immunogenicity subcohort and infection case participants. (The pre-defined time points are Day 1, 35.)

1. Individual anti-Spike antibody concentration at each pre-defined time

point

2. Individual anti-Spike antibody fold-rise concentration post-vaccination relative to baseline at each pre-defined post-vaccination time point
3. 2-fold-rise and 4-fold rise (fold rise in anti-Spike antibody concentration [post/pre] ≥ 2 and ≥ 4, 2FR and 4FR) at each pre-defined post-vaccination time point
4. Pseudovirus-nAb responders, at each pre-defined timepoint defined as participants who had baseline values below the LLOQ with detectable pseudovirus-nAb ID50 titers above the assay LLOQ or as participants with baseline values above the LLOQ with a 4-fold increase in pseudovirus-nAb ID50 titers

Summaries of the immunogenicity data will be reported in tables. In particular, the tables will include, for each pre-defined post-baseline time point:

1. For each binding antibody marker, the estimated percentage of participants defined as responders, and with concentrations $\geq$ 2x LLOQ or $\geq$ 4 x LLOQ, will be provided with the corresponding 95% CIs using the Clopper-Pearson method.

   In addition, the estimated percentage of participants defined as responders, participants with 2-fold rise (2FR), and participants with 4-fold rise (4FR) will be provided with the corresponding 95% CIs using the Clopper-Pearson method.
2. For the ID50 pseudo-virus neutralization antibody marker, the estimated percentage of participants defined as responders, participants with 2-fold rise (2FR), and participants with 4-fold rise (4FR) will be provided with the corresponding 95% CIs using the Clopper-Pearson method
3. Geometric mean titers (GMTs) and geometric mean concentrations (GMCs) will be summarized along with their 95% CIs using the t-distribution approximation of log-transformed concentrations/titers (for each of the four Spike-targeted marker types including pseudovirus-nAb ID50 and ID80, as well as for binding Ab to N).

4. Geometric mean titer ratios (GMTRs) or geometric mean concentration ratios (GMCRs) are defined as geometric mean of individual titers/concentration ratios (post-vaccination/pre-vaccination for each injection)
5. GMTRs/GMCRs will be summarized with 95% CI (t-distribution approximation) for any post-baseline values compared to baseline, and post-Day 35 values compared to Day 35
6. The ratios of GMTs/GMCs will be estimated between groups with the two-sided 95% CIs calculated using t-distribution approximation of log-transformed titers/concentrations [the groups compared are vaccine recipient non-cases vs. vaccine recipient breakthrough cases used for Day 35 marker correlates analyses (Post Day 35 cases)].
7. The differences in the responder rates, 2FRs, 4FRs between groups will be computed along with the two-sided 95% CIs by the Wilson-Score method without continuity correction (Newcombe, 1998) (the groups for comparison are as described in the previous bullet).

All of the above point and confidence interval estimates will use inverse probability of antibody marker sampling weighting in order that estimates and inferences are for the population from which the whole study cohort was drawn. In two-phase sampling data analysis nomenclature, the "phase 1 ptids" are the per-protocol individuals excluding individuals with a COVID failure event or any other evidence of SARS-CoV-2 infection < 7 days post Day 35 visit (the RT-PCR assay is used to define any evidence of SARS-CoV-2 infection). The "phase 2 ptids" are then the subset of these phase 1 ptids in the immunogenicity subcohort with Day 1 and Day 35 Ab marker data available. Thus, marker data for the COVID endpoint cases outside the subcohort will not be used in immunogenicity analyses; these cases are excluded from immunogenicity analyses.

The estimated weight $\hat{w}_{subcohort.35x}$ is the inverse sampling probability weight, calculated as the empirical fraction (No. Day 35 phase 1 ptids / No. Day 35 phase 2 ptids) within each of the baseline strata [(vaccine, placebo) $\times$ (baseline negative, baseline positive) $\times$ (demographic strata)]. For individuals outside the phase 1 ptids, $\hat{w}_{subcohort.35x}$ is assigned the missing value code NA.

All other individuals have a positive value for $\hat{w}_{subcohort.35x}$, including cases not in the subcohort. This weight is only used for case outcome-status blinded immunogenicity inferential analyses. Note that $\hat{w}_{subcohort.35x}$ is used for all immunogenicity analyses, which are based solely on the immunogenicity subcohort, for Day 1 and Day 35 markers. (Not used for correlates analyses.)

Tables will be provided separately for (1) baseline negative individuals, (2) baseline positive individuals, (3) baseline negative individuals by subgroup defined as in Table 2, and (4) baseline positive individuals by the same subgroups as in (3). Each table will show data for all available time points and for each of the vaccine and placebo arms.

Table 2: Baseline Subgroups that are Analyzed[1].

**Age:** 18-64, $\geq$ 65
**Coexisting conditions:** Yes, No
18-64 Coexisting conditions, 18-64 No coexisting conditions, $\geq$ 65 Coexisting conditions, $\geq$ 65 No coexisting co[illegible]
**Sex:** Male, Female
**Age x Sex:**
18-64 Male, 18-64 Female, $\geq$ 65 Female, $\geq$ 65 Male
**Hispanic or Latino Ethnicity:** Hispanic or Latino, Not Hispanic or Latino
**Race or Ethnic Group:**
White Non-Hispanic[2], Black, Asian, American Indian or Alaska Native (NatAmer)
Native Hawaiian or Other Pacific Islander (PacIsl), Multiracial,
Other, Not reported, Unknown
**Underrepresented Minority Status in the U.S.:**
Communities of color (Comm. of color), White[2]
**Age x Underrepresented Minority Status in the U.S.:**
Age $\geq$ 65 Comm. of color, Age $<$ 65 Comm. of color, Age $\geq$ 65 White, Age $\geq$ 65 White

[1]All analyses are done within strata defined by randomization arm and baseline positive/negative status, such that these variables are not listed here as subgroups for analysis.

[2]White Non-Hispanic is defined as Race=White and Ethnicity=Not Hispanic or Latino. All of the other Race subgroups are defined solely by the Race variable, with levels Black, Asian, American Indian or Alaska Native, Native Hawaiian or Other Pacific Islander, Multiracial, Other, Not reported, Unknown. Communities of color is defined by the complement of being known White Non-Hispanic.

For comparing antibody levels between groups, the following groups are compared:

- Baseline negative vaccine vs. baseline negative placebo
- Baseline positive vaccine vs. baseline positive placebo
- Baseline negative vaccine vs. baseline positive vaccine
- Within baseline negative vaccine recipients, compare each of the following pairs of subgroups listed in Table 2: Age ≥ 65 vs. age < 65; risk for severe COVID: at risk vs. not at risk; age ≥ 65 at risk vs. age ≥ 65 not at risk; age < 65 at risk vs. age < 65 not at risk; male vs. female; Hispanic or Latino ethnicity: Hispanic or Latino vs. Not Hispanic or Latino; Underrepresented minority status: Communities of color vs. White Non-Hispanic (within the U.S.).

The entire immunogenicity analysis is done in the per-protocol cohort with Day 1 and Day 35 marker data available (the two-phase sample).

6.1.2 Graphical description of antibody marker data

The Day 1, 35 antibody marker data collected from the immunogenicity subcohort participants will be described graphically. These data are representative of the entire study cohort. Importantly, only antibody data from the immunogenicity subcohort are included (i.e., no data from cases outside the subcohort are included). This makes the analyses unsupervised (independent of case-control status), enabling interrogation and optimization of the antibody biomarkers prior to the inferential correlates analyses.

Plots are developed for the following purposes. All of the analyses are done separately within each of the four subgroups defined by randomization arm cross-classified with baseline negative/positive status. In addition, many of the descriptive analyses will also be done separately for each demographic subgroup of interest listed above. For descriptive plots of individual marker data points that pool over one or more of the baseline strata subgroups, plots show all observed data points.

For each antibody marker readout, both Day 35 and baseline-subtracted Day 35 readouts are of interest. We will refer to the latter as 'delta.' All readouts, including delta, will be plotted on the $\log_{10}$ scale, with plotting labels on the

natural scale. As such, delta is $\log_{10}$ fold-rise in the marker readout from baseline.

The following descriptive graphical analyses are done.

1. The distribution of each antibody marker readout at Day 1 and Day 35 will be described with plots of empirical reverse cumulative distribution functions (rcdfs) and boxplots (including individual data points) within each of the four groups defined by randomization arm (vaccine, placebo) and baseline positivity stratum (seronegative, seropositive). Inverse probability of sampling into the subcohort weights ($\hat{w}_{subcohort.35x}$) are used in the estimation of the rcdf curves; henceforth we refer to these weights as "inverse probability of sampling" (IPS) weights. Analyses of Day 1 markers always pool across vaccine and placebo recipients given that the two subgroups are the same at baseline.

2. Plots are arranged to compare each Day 35 marker readout between randomization arms within each of the baseline seropositive and baseline seronegative subgroups.

3. Plots are also arranged to compare each Day 35 marker readout between baseline serostatus groups within each randomization arm.

4. The correlation of each antibody marker readout among Day 1 and Day 35, and between Day 1 and fold-rise to Day 35 (delta), is examined within each randomization arm and baseline positivity stratum. Pairs plots/scatterplots will be used, annotated with baseline strata-adjusted Spearman rank correlations, implemented in the PResiduals R package available on CRAN. For calculating the correlation within each randomization arm and baseline positivity stratum, because PResiduals does not currently handle sampling weights, the correlation estimates are computed as follows: For each re-sampled data set in the second approach to graphical plotting, the covariate-adjusted Spearman correlation is calculated. The average of the estimated correlations across re-sampled data sets is reported.

5. The correlation of each pair of Day 1 antibody marker readouts are com-

pared within each baseline positivity stratum, pooling over the two randomization arms. Pairs plots/scatterplots and baseline-strata adjusted Spearman rank correlations are used, with covariate-adjusted Spearman rank correlations computed as described above. The same analyses are done for each pair of Day 35 antibody marker readouts.

6. Point estimates of Day 35 marker positive response rates for each randomization arm within each baseline positivity stratum are provided. The point and 95% CI estimates include all of the data and use IPS weights.

6.2 Methods for Positive Response Calls for bAb and nAb Assays

As noted above, binding antibody responders at each pre-defined timepoint are defined as participants with concentration above the specified positivity cut-off, with a separate cut-off for each antigen Spike, RBD, N (10.8424, 14.0858, and 23.4711, respectively, in BAU/ml). This approach is used for each of the Spike and RBD and N protein antigen targets.

Pseudovirus neutralization responders at each pre-defined timepoint are defined as participants who had baseline ID50 values below the LOD with detectable ID50 neutralization titer above the assay LOD, or as participants with baseline values above the LOD with a 4-fold increase in neutralizing antibody titer. Otherwise a value is negative for pseudovirus neutralization.

6.3 SARS-CoV-2 Antigen Targets Used for bAb and nAb Markers

The homologous vaccine strain antigens are used for the immune correlates analyses for the bAb markers, whereas the homologous vaccine strain with D614G mutation is used for the pseudovirus nAb markers.

7 Baseline Risk Score (Proxy for SARS-CoV-2 Exposure)

We will develop baseline risk score for US only (COUNTRY==“USA”) because there are no cases in the vaccine arm and there is a single case in

the placebo arm from outside of the US in the primary analysis cohort (PP-EFFFL==”Y”) for the primary endpoint (PARAMCD ==”PCRMMS”).

Imputation of marker variables, if needed, will be done for US and Mexico together. Since we may need separate immunogenicity reports for US and Mexico, we will create two analysis-ready datasets, one for US and one for Mexico. This requires some tailoring of the correlates_processing code.

Restrictions on Treatment assignment (TRT01P==”Placebo” or ”SARS-CoV-2rS”), PP status (PARAMCD ==”PCRMMS1”), country and case accrual period were applied as described. The list of baseline covariates potentially relevant for SARS-CoV-2 exposure and risk of COVID was specified below (xxTable S4 in the Supplementary Material) (See the .tex file for corresponding variable names in the dataset): Age, Sex at birth (Male/Female), Race (7 categories), Ethnicity (3 categories), Height, Weight, BMI, and Protocol-defined high-risk (yes/no).

Based on these covariates, a baseline risk score is developed and controlled for in correlates analyses to adjust for potential confounding. The risk score is defined as the logit of the predicted outcome probability from a regression model estimated using the ensemble algorithm superlearner (i.e. stacking), where this logit predicted outcome is scaled to have empirical mean zero and empirical standard deviation one. The settings of superlearner (i.e., loss function, cross-validation technique, library of learners) that are used for implementation of superlearner for building a baseline risk score are described in Section 9.4.

The development of risk score will involve training the superlearner using placebo arm data and predictions made on vaccine arm data (CV-predictions will be made on placebo arm data). In both arms, risk score development will be restricted to baseline negative per-protocol subjects with cases as COVID endpoints starting post-enrollment. The CV-prediction performance of superlearner (CV-AUC calculation and CV-ROC curves) will be derived with cases as COVID endpoints starting post-enrollment as well. The prediction performance of superlearner (AUC calculation and ROC curve) in the vaccine arm, however, will be restricted to the same set of vaccine recipients as used

in the correlates analyses with cases considered as COVID endpoints starting 7 days post second vaccination visit and non-cases as participants with follow-up beyond 7 days post second vaccination visit and never registered a COVID endpoint.

Independent of the superlearner risk score, important individual risk factors are also specified for inclusion as adjustment factors in correlates analyses. In particular, in addition to the risk score the at-risk indicator and the communities of color indicator are adjusted for in all correlates analyses. This choice is justified by the epidemiological data showing that these two indicators are strong infection and COVID-19 risk factors, and making use of the flexibility of super learner to develop a model for how age relates to risk.

Henceforth we refer to the baseline variables that are adjusted for in correlates analyses as "baseline factors" which, depending on the risk score results and performance, will consist of only the individual key risk factors, or key individual risk factors plus the baseline risk score.

8 Correlates Analysis Descriptive Tables by Case/Non-Case Status

The key table summarizing the distribution of each of the two antibody markers at the Day 1 and 35 times points is listed below. For each time point Day 1 and Day 35 separately, the positive response rate with 95% CI, and the GMT or GMC with 95% CI, is reported for each of of the case and non-case groups. In addition, the point and 95% CI estimate of the difference in positive response rate (non-cases vs. cases) and the GMT or GMC ratio (non-cases/cases), is reported.

- Immunogenicity table: Antibody levels in the baseline SARS-CoV-2 negative per-protocol cohort (vaccine recipients). Post Day 35 cases are baseline negative per-protocol vaccine recipients with the symptomatic infection COVID-19 primary endpoint diagnosed starting 7 days after the Day 35 study visit. Non-cases/Controls are baseline negative per-protocol vaccine recipients sampled into the immunogenicity subcohort with no COVID primary endpoint up to the time of data cut and no

evidence of SARS-CoV-2 infection up to six days post Day 35 visit.

The point and confidence interval estimates are computed using inverse probability sampling weights $\hat{w}_{subcohort.35x}$ for Post Day 35 cases and for Non-cases, as defined in Section 9.3.1.

9 Correlates of Risk Analysis Plan

This analysis plan for CoRs and CoPs focuses on the COVID primary endpoint, with its continuous failure times (failure time defined by the day of the event) and no competing risks.

9.1 CoR Objectives

The following CoR objectives are assessed in baseline seronegative per-protocol vaccine recipients:

1. **Univariable CoR** To assess each individual Day 35 antibody marker as a CoR of outcome in vaccine recipients, adjusting for baseline factors (See Section 7)

9.2 Outline of the Set of CoR Analyses

The univariable CoR objective is addressed by Cox proportional hazards regression and nonparametric threshold regression. All of these analyses are implemented in automated and reproducible press-button fashion.

9.3 Day 35 Markers Assessed as CoRs and CoPs

The following two markers at Day 35 are assessed as CoRs and CoPs, usually as quantitative variables and in some analyses as ordered trinary variables or binary variables, all of which do not subtract Day 1 (baseline) values:

1. binding Ab to Spike (IgG BAU/ml)
2. pseudovirus neutralization ID50 (IU50/ml)

For all univariable CoR analyses (first objective), the non-baseline subtracted versions of the Day 35 antibody markers are studied; the baseline-subtracted

versions are not studied given that the analyses are done in the baseline negative cohort for which Day 1 readouts will generally be negative.

9.3.1 Inverse probability sampling weights used in CoR analyses

In section 6.1, estimated inverse probability sampling (IPS) weights $\hat{w}_{subcohort.35x}$ were defined for per-protocol immunogenicity subcohort members, for the purpose of immunogenicity analyses. This section describes the IPS weight used for Day 35 marker correlates analyses ($\hat{w}_{35.x}$).

For baseline sampling stratum x [(vaccine, placebo) $\times$ (demographic strata)], the IPS weight $w_{35.x}$ assigned to a non-case participant in stratum x is defined by $\hat{w}_{35.x} = 1/\hat{\pi}_{35}(x) = N_x/n_x$, where N_x is the number of stratum x vaccine recipient non-cases in the Per-Protocol Baseline Negative (PPBN) cohort and n_x is the number of these participants that also have Day 1, 21, and 35 marker data available, where participants with any evidence of SARS-CoV-2 infection before 7 days post Day 35 visit are excluded from the counts N_x and n_x. For non-case participant i in the immunogenicity subcohort, $\hat{w}_{35.i} = 1/\hat{\pi}_{35}(X_i)$ denotes the weight $\hat{w}_{35.x}$ for this individual's sampling stratum. All Post Day 35 cases are assigned sampling weight N_1/n_1 where N_1 is the total number of vaccine recipient cases in the PPBN cohort restricting to cases with event time starting 7 days post Day 35, and n_1 is the number of these participants that also had the Day 1 and 35 markers measured, and again participants with any evidence of SARS-CoV-2 infection $<$ 7 days post Day 35 visit are excluded from the counts N_x and n_x.

In terms of two-phase sampling data analysis nomenclature, for the Day 35 marker analyses "phase 1 ptids" are defined as the entire PPBN cohort except excluding participants with any evidence of SARS-CoV-2 infection $<$ 7 days post Day 35 visit. The "phase 2 ptids" are then the subset of these phase 1 ptids with Day 1, 21, and 35 Ab marker data available. Thus the weight $\hat{w}_{35.x}$ is the inverse sampling probability weight, calculated as the empirical fraction (No. phase 1 ptids / No. phase 2 ptids) within each of the baseline negative strata (defined by PPBN vaccine group cases, PPBN placebo group cases, PPBN vaccine group non-cases divided into the demographic strata, and PPBN placebo group non-cases divided into the demographic strata).

For baseline negative individuals outside the phase 1 ptids, $\hat{w}_{35.x}$ is assigned the missing value code NA. All other individuals have a positive value for $\hat{w}_{35.x}$.

9.3.2 Choice of regression methods

Time-to-event methods of Day 35 marker correlates analyses use the Day 35 visit date as the time origin.

The IPWCC Cox regression model designed for case-cohort sampling designs will be used for estimation and inference on hazard ratios of outcomes by Day 35 marker levels, and for estimation and inference on marginalized marker-conditional cumulative incidence over time. The models will be fit using the *survey* R package available on CRAN, and will adjust for the baseline factors. We use a method from the survey package that assumes without replacement two-phase sampling and not Bernoulli sampling, which matches the sampling design and approach to weight estimation (**?**).

The final time point t_F of follow-up for correlates analyses is taken to be the latest COVID outcome event time. Let T be the failure time, S a Day 35 marker of interest, and X the vector of baseline factors that are adjusted for. With $S_1(t|s,x) = P(T > t|S = s, X = x, A = 1)$, the Cox model fit yields an estimate of $S_1(t|s, X_i)$ for each individual i in the phase-two sample. The marginalized conditional risk $risk_1(t|s) = E_X[P(T \leq t|s, X, A = 1)]$ through time t (for all times t through t_F simultaneously) is estimated based on the equation

$$risk_1(t|s) \; = \; \int (1 - S_1(t|s,x))dH(x) \qquad (1)$$

where $H(\cdot)$ is the distribution of X in $A = 1$ individuals.

The function $risk_1(t|s)$ can be estimated by

$$\widehat{risk}_1(t|s) = \frac{\sum_{i=1}^{n} \frac{1}{\hat{\pi}(X_i)}(1 - \hat{S}_1(t|s, X_i))}{\sum_{i=1}^{n} \frac{1}{\hat{\pi}(X_i)}}, \qquad (2)$$

where n is the number of participants with phase-two data.

The bootstrap is used to obtain 95% pointwise confidence intervals for $risk_1(t_F|s)$.

The bootstrap process will be performed by resampling with replacement the subjects within the subcohort and the subjects outside the subcohort separately within each stratum and by resampling with replacement subjects with undetermined stratification variables. Across all bootstrap samples, the number of participants in each stratum in the immunogenicity subcohort remains fixed, but the number of cases does not stay the same.

The results of the above Cox modeling will be output in a variety of ways:

1. Plot $\widehat{risk}_1(t_F|s)$ vs. s with 95% CIs for continuous $S = s$ varying over its whole range. Include on the plot the estimate of $\widehat{risk}_0(t_F)$ with a 95% CI for the placebo arm (horizontal bands), computed by a Cox model marginalizing over the same baseline factors as for the analysis of the vaccine arm.

2. Based on a fit of the Cox model to a nominal categorical antibody marker defined as the tertiles of S, plot $\widehat{risk}_1(t|s)$ for each category of S values with 95% CIs, for all time points t from Day 35 through t_F. If more than 20% of vaccine recipients have S below the LOD of the assay, then the categories instead will be (1) values $\leq$ LOD; (2) values below the median of values $>$ LOD; (3) values above the median of values $>$ LOD. Include on the plot the estimated curve $\widehat{risk}_0(t)$ with 95% CIs for the placebo arm, computed by a Cox model marginalizing over the same baseline factors as for the analysis of the vaccine arm.

3. Tabular reporting of the hazard ratio per 10-fold change in the quantitative Day 35 antibody marker with 95% confidence interval and 2-sided p-value.

4. Tabular reporting of the hazard ratio for the Middle and Upper categories of the categorical Day 35 antibody marker vs. the Lower category, with 95% confidence interval and 2-sided p-value, as well as a global generalized Wald two-sided p-value for whether the hazard rate of the endpoint varies across the three categories. The table includes the attack rate (with no. of cases / no. at risk) through t_F for each of the three vaccine

marker subgroups and for the placebo arm.

5. Report point and 95% CI estimates for the hazard ratio per 10-fold change in the Day 35 antibody marker, for the entire per-protocol baseline negative vaccine cohort and for each of the baseline demographic strata subgroups defined in Table 2 (reported via forest plotting).

6. Westfall-Young (1997) q-values and FWER-adjusted p-values for the generalized Wald tests are included in the table.

The bootstrap is used to calculate 95% pointwise CIs for $risk_1(t_F|s)$ in s. The 2-sided Wald p-value for testing the regression coefficient of the marker in the Cox model provides a valid test of the null hypothesis $H_0 : risk_1(t_F|s) = risk_1(t_F)$ for all s, and is reported.

In addition, the same Cox model analysis will be used to estimate the alternative marginalized conditional risk parameter defined by $risk_1(t|S \geq s)$ where $risk_1(t|S \geq s) = E_X[P(T \leq t|S \geq s, X, A = 1)]$, which can be estimated by

$$\widehat{risk}_1(t|S \geq s) = \frac{\sum_{i=1}^{n} \frac{1}{\hat{\pi}(X_i)}(1 - \hat{S}_1(t|S \geq s, X_i))}{\sum_{i=1}^{n} \frac{1}{\hat{\pi}(X_i)}}.$$

This parameter is useful because typically subgroups of interest are defined by having marker response above a threshold. We will plot $\widehat{risk}_1(t_F|S \geq s)$ vs. s with 95% CIs for continuous S with s varying over the range of S in which the number of cases to estimate $\hat{S}_1(t|S \geq s, X_i)$ is 5 or more. This type of analysis is also included because it analyzes the same parameter as the nonparametric threshold estimation method described below, providing a way to address the threshold question both by Cox modeling and by nonparametric analysis.

9.3.3 Univariate CoR: Nonparametric threshold regression modeling

The targeted minimum loss-based estimation (TMLE) method of van der Laan et al. (2022) extension of the nonparametric CoR threshold estimation method of Donovan et al. (2019) is applied to each of the non-baseline subtracted antibody markers at Day 35, using the version that defines the binary outcome $Y = I(T \leq t)$ of interest as $Y = 1$ if a COVID endpoint occurred during the blinded period of follow-up and $Y = 0$ otherwise, and accounts

for right-censoring times of participants. The analyses adjust for the same baseline factors X as used in the Cox model CoR analyses.

The extension adjusts for baseline covariates by estimating the conditional mean function $E[Y|S \geq s, X, A = 1]$ using discrete-SuperLearner and then empirically averaging over the baseline covariates X to estimate the marginal risk $risk_1^Y(S \geq s) = E_X[P(Y = 1|S \geq s, X, A = 1)]$ for each threshold s of the the antibody marker in a specified discrete set. We do not perform pooled regression across the thresholds s, which ensures we are totally nonparametric in estimating the threshold dependence of $risk_1^Y(S \geq s)$ on s. The SuperLearner library includes only L1-penalized logistic regression (glmnet), because of the small number of vaccine breakthrough cases with Day 35 antibody data. An advantage of the nonparametric CoR threshold method compared to Cox modeling that specifies a log linear hazard ratio with the marker is that it can potentially detect a threshold of very low risk. The method is implemented with and without the monotonicity constraint that $risk_1^Y(S \geq s)$ is monotone non-increasing in s, where the results assuming monotonicity are reported unless there is evidence for violation of this assumption.

The results are reported in the same way that Donovan et al. (2019) reports results in its Figure 2, where point estimates, pointwise 95% confidence bands, and simultaneous 95% confidence bands for $risk_1^Y(S \geq s)$ are plotted for a range of threshold values. The simultaneous confidence bands cover the entire curve in s with at least 95% probability and are useful for judging whether risk varies over threshold subgroups, whereas the pointwise 95% confidence bands are useful for quantifying precision at particular threshold values. The method uses the same empirical two-phase sampling estimated weights (IPS weights) as used for the other univariable IPWCC CoR analyses. In addition, for each pre-specified risk threshold c set to take values over a grid with lowest value 0, the method is applied to estimate the inverse function $s_c = inf\{s : E_X[P(Y = 1|S \geq s, A = 1, X] \leq c\}$, where s_c is estimated by substitution of the marginal risk function estimate. Note that the substitution estimator of s_c requires that the marginal risk function is estimated for all thresholds, which is computationally infeasible. Instead,

we estimate the marginal risk function on a sufficiently large discrete set and linearly interpolate to obtain marginal risk estimates for all thresholds outside the discrete set. In order for this estimand to be well defined, we operate (for this estimand only) under the assumption that $s \mapsto risk_1^Y(S \geq s)$ is monotone. For the substitution-based estimator of the inverse function s_c to be well-defined, we require the estimate of $s \mapsto risk_1^Y(S \geq s)$ to be monotone as well. If there is evidence that the function estimate is not monotone then we replace the estimate with its monotone projection, which preserves its theoretical properties (Westling, van der Laan, Carone, 2020).

9.3.4 P-values and Multiple hypothesis testing adjustment for CoR analysis

In general, p-values are only reported from pre-specified and automated (press-button) analyses. For the CoR analyses, p-values are reported for the univariable Cox regression analyses of the four specified Day 35 antibody marker variables. Two-sided p-values for hypothesis testing of a Day 35 marker CoR are calculated both for the Cox regression of quantitative markers (two-sided Wald tests), and for the Cox regression of markers binned into tertiles (two-sided Generalized Wald tests). Therefore a total of eight 2-sided p-values for Day 35 CoRs are calculated. However, if there are fewer than 20 vaccine breakthrough cases with Day 35 antibody data, then no p-values will be reported for the tertilized marker correlates analyses.

It is not completely clear whether to perform multiple hypothesis testing adjustment, given the expectation that the correlations among the markers are high, and possibly very high, meaning that multiplicity correction could incur a relatively high cost on the false negative error rate. However, given that robust evidence supporting an antibody marker as a CoR will be required for qualifying a marker, we will conduct multiplicity adjustment for CoR analysis, as the ability to make an inference that a marker passed pre-specified multiplicity adjusted criteria should aid an overall evidence package for establishing a validated or non-validated surrogate endpoint. Therefore, multiplicity adjustment is performed across the set of 2-sided p-values.

A permutation-based method (Westfall et al., 1993) will be used for both family-wise error rate (Holm-Bonferroni) and false-discovery rate (q-values;

Benjamini-Hochberg) correction. 10^4 replicates of the data under the null hypotheses will be created by randomly resampling the immunologic biomarkers with replacement. For each Cox regression CoR analysis the unadjusted p-value, the FWER-adjusted p-value, and the q-value is reported for whether there is a covariate-adjusted association, where all p-values and q-values are 2-sided. The FWER-adjusted p-values and q-values are computed pooling over both the quantitative marker and tertilized marker CoR analyses. However, if there are fewer than 20 vaccine breakthrough cases with Day 35 antibody data, then no p-values are reported for the tertilized marker CoR analyses, in which case the FWER-adjusted p-values and q-values are computed pooling for the quantitative marker CoR analyses only. As a guideline for interpreting CoR findings, markers with FWER-adjusted p-value ≤ 0.05 are flagged as having statistical evidence for being a CoR. Additionally, markers with unadjusted p-value ≤ 0.05 and q-value ≤ 0.10 are flagged as having a hypothesis generated for being a CoR.

9.4 Implementation of superlearner for baseline risk score development

For baseline risk score development, Superlearner is applied to the placebo arm only, as mentioned in Section 7. The following details are used in the implementation of superlearner:

- Pre-scale each quantitative and ordinal variable to have empirical mean 0 and standard deviation 1.
- For the Novavax PREVENT-19 trial, there are 60 endpoint cases starting Day 1 post-enrollment. So, superlearner modeling was conducted using maximum of 20 risk score variables and 5-fold cross-validation with negative log-likelihood loss function.
- The library of adaptive and non-adaptive learners and the screens selected for superlearning are shown in Table 3. Most of the learners are non-data-adaptive type learning algorithms, such as parametric regression models (e.g., generalized linear models [glms]), which are simple, stable, and advantageous for an application with a limited number of endpoint events. Data-adaptive type algorithms are also included if the

number of endpoint events is high enough, for increasing flexibility of modeling and reducing the risk of model misspecification: SL.ranger, SL.gam, and SL.xgboost. All of the selected learners are coded into the SuperLearner R package.

- Screens used will be: 1) glmnet (lasso) pre-screening (with default tuning parameter selection), 2) logistic regression univariate 2-sided p-value screening (at level $p < 0.10$), and 3) high-correlation variable screening (described below).
- Include high-correlation variable screening, not allowing any pair of input variables to have Spearman rank correlation $r > 0.9$.
- The superlearner is conducted averaging over 10 random seeds, to make results less dependent on random number generator seed.
- No IPS weighting is needed.
- Two levels of cross-validation are used:
  - Outer level: CV-AUC computed over 5-fold cross-validation repeated 10 times to improve stability
  - Inner level: 5-fold inner CV used to estimate ensemble weights with no more than $\max(20, \text{floor}(n_p/20))$ input variables included in each model, where n_p is the number of evaluable placebo arm cases.
- Results for comparing classification accuracy of different models are based on point and 95% confidence interval estimates of cross-validated area under the ROC curve (CV-AUC) and difference in CV-AUC as a predictiveness metric (Hubbard et al., 2016; ?). Results are presented as forest plots of point and 95% confidence interval estimates similar to those used in Figure 3 of Neidich et al. (2019) and Magaret et al. (2019). CV-AUC is estimated using the R package *vimp* available on CRAN.

Table 3: Learning Algorithms in the Superlearner Library of Estimators of the Conditional Probability of Outcome, for Building the Baseline Risk Score Based on the Placebo Arm[1].

Algorithms	Screens/ Tuning Parameters
SL.mean	None
SL.glm	Low-collinearity and (All, Lasso, LR)[2]
SL.glm.interaction	Low-collinearity and (Lasso, LR)
SL.glmnet	(alpha=1; All)
SL.gam	Low-collinearity and (Lasso, LR)
SL.xgboost[3]	All and (maxdepth,shrinkage,balance)= (4, 0.1, no)
SL.ranger[3]	All and balance = no

[1]All continuous and ordinal covariates are pre-standardized to have empirical mean 0 and standard deviation 1.
[2]**All** = include all variables; **Lasso** = include variables with non-zero coefficients in the standard implementation of SL.glmnet that optimizes the lasso tuning parameter via cross-validation; **Low-collinearity** = do not allow any pairs of quantitative variables with Spearman rank correlation > 0.90; **LR** = Univariate logistic regression Wald test 2-sided p-value < 0.10.
[3]Covariate balancing (if requested) is done using option `scale_pos_weight` in SL.xgboost and option `case.weights` in SL.ranger.

Table 4: Learning Algorithms in the Superlearner Library of Estimators of the Conditional Probability of Outcome: Simplified Library in the Event of Fewer than 50 Placebo Arm Cases for an Analysis, for Building a Baseline Behavioral Risk Score in Novavax PREVENT-19[1].

Algorithms	Screens/ Tuning Parameters
SL.mean	None
SL.glm	Low-collinearity and (All, Lasso, LR)[2]
SL.glmnet	alpha=0, 1
SL.xgboost	(maxdepth,shrinkage,balance[3])= (2, 0.1, yes) (2, 0.1, no) (4, 0.1, yes) (4, 0.1, no)
SL.ranger	balance = (yes, no)

[1]All continuous and ordinal covariates are pre-standardized to have empirical mean 0 and standard deviation 1.
[2]**All** = include all variables; **Lasso** = include variables with non-zero coefficients in the standard implementation of SL.glmnet that optimizes the lasso tuning parameter via cross-validation; **Low-collinearity** = do not allow any pairs of quantitative variables with Spearman rank correlation > 0.90; **LR** = Univariate logistic regression Wald test 2-sided p-value < 0.10.
[3]Covariate balancing (if requested) is done using option `scale_pos_weight` in SL.xgboost and option `case.weights` in SL.ranger.

In order to evaluate the performance of the superlearner estimated model, derived using the learning algorithms specified in Table 3, the CV-AUC is estimated with a 95% confidence interval (Hubbard et al., 2016; Williamson et al., 2022). The point and 95% confidence interval estimates of CV-AUC are reported in a forest plot, which provide a way to discern which antibody assays and readouts/markers provide the most information in predicting COVID or other outcomes. As noted above CV-AUC is estimated using the R package *vimp* available on CRAN.

If there are fewer than 50 placebo arm COVID-19 cases included in a correlates analysis, then the library of learners will be simplified to that specified in Table 4.

In addition, for selected variable sets, similar forest plots will be made comparing performance of the various estimated models (e.g., by individual learning algorithm types such as lasso), including discrete superlearner and superlearner models. The plot will be examined to determine which individual learning algorithm types are performing the best.

Cross-validated ROC curves are plotted for the superlearner estimated models for each of the input variable sets. In addition, boxplots of cross-validated estimated probabilities of outcome by case-control status (as estimated from the superlearner models) are plotted.

10 Correlates of Protection: Generalities

In general, for all of the correlate of protection analyses, the same antibody markers are assessed that were analysed as correlates of risk: the Day 35 antibody markers not subtracting for the Day 1 baseline readout are used. Each of the Day 35 antibody biomarkers are separately studied as CoPs by one analysis approach summarized below.

11 Correlates of Protection: Interventional Effects

In these analyses, we seek to understand whether, how, and to what extent Day 35 antibody markers impact vaccine efficacy in causal ways. We describe three approaches to this problem. Each involves consideration of a binary counterfactual outcome $Y(a, s)$ (e.g., indicator of the COVID disease endpoint by a pre-specified time) under a hypothetical intervention that both sets randomization assignment $A = a$ and sets the Day 35 immunologic marker S to a fixed value or based upon a random draw from a analyst-specified distribution. Below, we assume that S is scalar-valued, but some of the approaches below naturally extend to the case where a vector of immunologic markers are considered (currently such analyses are not planned). Given the central goal to develop a parsimonious surrogate endpoint based on a single immunoassay, the main analysis will use each of the methods to assess each of the four quantitative readouts (not baseline-subtracted) separately as CoPs, adjusting for the same set of baseline covariates as used in the CoR analyses previously described in Section 9.

11.1 CoP: Controlled Vaccine Efficacy

We first describe the controlled vaccine efficacy curve defined as

$$\text{CVE}(s) = 1 - \frac{P(Y(1, s) = 1)}{P(Y(0) = 1)} \ .$$

The value of CVE(s) represents the relative decrease in the probability of endpoint occurrence achieved by administering vaccine and setting Day 35 immunologic marker level to s compared to the placebo control intervention, for which the Day 35 immunologic marker level is structurally set equal to the lowest possible value. Under our approach, the value of CVE(s) is assumed to be monotone non-decreasing in s; in other words, vaccine efficacy can only potentially be improved by setting greater marker levels. The extent to which the marker plays a role in determining vaccine efficacy can be determined by the degree of flatness of the graph of CVE(s) versus s.

In addition, because the primary study cohort for correlates analysis is naive to SARS-CoV-2, each of the Day 35 markers S has no variability in the

placebo arm [all values are 'negative,' below the positivity cut-off of the binding antibody variables and below the lower limit of detection (LOD) of the neutralizing antibody variables]. Therefore, advantageously in this setting $CVE(s)$ has a special connection to the mediation literature, where $CVE(s = LOD)$ is the natural direct effect, and vaccine efficacy is 100% mediated through S if and only if $CVE(s = LOD) = 0$. Therefore inference on $CVE(s = LOD)$ evaluates full mediation.

Since $P(Y(0) = 1) = P(Y = 1 \mid A = 0)$ in view of vaccine versus placebo randomization, the controlled vaccine efficacy CVE(s) at level s can be identified using the fact that

$$P(Y(1, s) = 1) = E\left[P(Y = 1 \mid S = s, A = 1, X)\right]$$

whenever $Y(1, s)$ and S are independent given $A = 1$ and a vector X of covariates, and $P(S = s \mid A = 1, X) > 0$ almost surely. In other words, identification of the controlled vaccine efficacy CVE(s) requires that a rich enough set of covariates be available so that deconfounding of the relationship between endpoint Y and marker S is possible in the subpopulation of vaccine recipients (no-unmeasured confounding assumption), and that marker level $S = s$ may occur within each subpopulation defined by values of the covariates X (positivity assumption).

11.1.1 Point and 95% confidence interval estimation of CVE(s) and of $RR_C(s_1, s_2) = (1 - \mathbf{CVE}(s_2))/(1 - \mathbf{CVE}(s_1))$ assuming the causal assumptions hold

In this subsection, we describe how the point and 95% confidence interval estimates for CVE(s) that are reported in the main article (**Fig. 4C**) and the Supplement are calculated, which assume that both causal assumptions mentioned above hold (no unmeasured confounders and positivity). In this subsection we also describe how the point and 95% confidence interval estimates for $RR_C(0, 1) = (1 - \text{CVE}(1))/(1 - \text{CVE}(0))$ for a binary marker S are calculated, with results for $S = 1$ representing the upper tertile and $S = 0$ representing the lower tertile reported in Supplementary Text S2. In the next subsection, we describe how the sensitivity analysis is conducted, which quantifies the sensitivity of the results to potential unmeasured confounding.

Gilbert, Fong, Kenny, and Carone (2022) details the inferential and sensitivity analysis approach, which was applied to the CYD14 and CYD15 dengue phase 3 data sets (Moodie et al., 2018); the same approach was applied to the current Novavax PREVENT-19 trial data set, given that the structure of the problem is the same. We summarize here the key details needed for understanding the analysis of the PREVENT-19 trial. Under the two causal assumptions, the numerator term $P(Y(1,s) = 1)$ of $\text{CVE(s)} = 1 - P(Y(1,s) = 1)/P(Y(0) = 1)$ is

$$P(Y(1,s) = 1) = E\left[P(Y = 1 \,|\, S = s, A = 1, X)\right] = risk_1(t_F|s),$$

as defined in Section 9.3.2, using the notation of Section 9.3.2. That section described the Cox modeling approach that was used to compute an estimate $\widehat{risk}_1(t_F|x)$ of $risk_1(t_F|s)$, where $Y = I(T \leq t_F)$, T is the time from the Day 35 marker measurement date until the COVID outcome starting 7 days post measurement date, and t_F is taken to be the latest COVID outcome event time, as noted earlier.

The same estimate $\widehat{risk}_1(t_F|s)$ is used to estimate the numerator term $P(Y(1,s) = 1)$ of CVE(s). That is, there is a harmonization of the correlate of risk and controlled VE analyses, where the estimate $\widehat{risk}_1(t_F|x)$ used for the former is also used for the numerator term $P(Y(1,s) = 1)$ of CVE(s) for the latter:

$$\widehat{\text{CVE(s)}} = 1 - \frac{\widehat{risk}_1(t_F|x)}{\widehat{P}(Y(0) = 1)}$$

(where we detail the estimator $\widehat{P}(Y(0) = 1)$ next).

To estimate the denominator of CVE(s), $P(Y(0) = 1) = P(Y = 1 \,|\, A = 0) = P(T \leq t_F|A = 0)$, note that there is no concern about unmeasured confounding given the study is randomized. While this denominator may be estimated validly ignoring the potential baseline confounders X, it was estimated with adjustment for the same covariates X adjusted for in the estimation of the numerator $\widehat{risk}_1(t_F|s)$. In particular, $E[P(Y = 1 \,|\, A = 0, X)]$ was estimated with a standard Cox model (without two-phase sampling, i.e., including all baseline negative per-protocol placebo recipients without evidence of infection

by 6 days post Day 35 visit, respectively), with point estimate the average of the fitted values $\widehat{E}[P(Y_i = 1|A_i = 0, X_i)]$ across the included placebo recipients. Then, the point estimate of CVE(s) is computed as one minus the ratio of the numerator point estimate divided by the denominator point estimate. Pointwise 95% confidence intervals for $CVE(s)$ were computed using the same set of bootstrap estimates of the numerator $\widehat{risk}_1(t_F|s)$ as used for the correlates of risk analysis, and also including bootstrap estimates of the denominator $\widehat{E}[P(Y = 1 \mid A = 0, X)]$. The nonparametric percentile bootstrap method was used for the confidence intervals.

11.1.2 Sensitivity analysis (to unmeasured confounding) for the Cox model controlled vaccine efficacy analysis

Sensitivity analysis is generally warranted when a no-unmeasured confounders assumption is made. The sensitivity analysis quantifies the rigor of evidence for a controlled VE CoP after accounting for potential bias from unmeasured confounding. We define S to be a controlled VE CoP if CVE(s) is monotone non-decreasing in s with CVE(s) < CVE(s') for at least some $s < s'$, where point and 95% confidence interval estimates of CVE(s) versus s, with built in robustness to unmeasured confounding, describe the strength of the CoP in terms of the amount and nature of increase. Because the denominator $P(Y(0) = 1)$ of CVE(s) does not depend on s, a controlled VE CoP can equivalently be defined as the numerator $P(Y(1, s) = 1)$ being monotone non-increasing in s with $P(Y(1, s) = 1) > P(Y(1, s') = 1)$ for at least some $s < s'$, where point and 95% confidence interval estimates of $P(Y(1, s) = 1)$ versus s indicate some robustness to unmeasured confounding.

Two sensitivity analyses are conducted, the first of which considers the binary immunologic marker S with 0 indicating the first tertile and 1 indicating the third tertile. The second sensitivity analysis considers the quantitative marker S varying over its full range.

As set-up for both sensitivity analyses, for any two marker values s_1 and s_2, define the controlled risk ratio

$$RR_C(s_1, s_2) = \frac{r_C(s_2)}{r_C(s_1)} = \frac{(1 - \text{CVE}(s_2))}{(1 - \text{CVE}(s_1))},$$

where $r_C(s) = P(Y(1, s) = 1)$ is the controlled risk at $S = s$. From the observed data without the causal assumptions, the statistical parameters $r_M(s) = risk_1(t_F|s)$ (the marginalized conditional risk) and

$$RR_M(s_1, s_2) = \frac{r_M(s_2)}{r_M(s_1)}$$

(the marginalized conditional risk ratio) can be estimated. Moreover, under the causal assumptions (no-unmeasured confounding and positivity), $r_M(s) = r_C(s)$ and $RR_M(s_1, s_2) = RR_C(s_1, s_2)$. Given that CoR analysis is based on observational data — the biomarker value is not randomly assigned — a central concern is that unmeasured or uncontrolled confounding of the association between S and Y could render $r_M(s) \neq r_C(s)$, biasing estimates of the causal parameters of interest $r_C(s)$ and $RR_C(s_1, s_2)$. Because we can never be certain that confounding is adequately adjusted for, sensitivity analysis is warranted, as considered in extensive literature — see, e.g., VanderWeele and Ding (2017) and references therein.

Sensitivity analysis is useful to evaluate how strong unmeasured confounding would have to be to explain away an observed causal association, that is, to determine the strength of association of an unmeasured confounder between S and Y needed for the observed exposure-outcome association to not be causal, $r_M(s) \neq r_C(s)$ and $RR_M(s_1, s_2) \neq RR_C(s_1, s_2)$. We follow the recommendation of VanderWeele and Ding (2017) to report the E-value as a summary measure of the evidence of causality, or, in our application, evidence of whether S is a controlled risk CoP based on variation in the controlled risk curve. We also include other closely related measures of sensitivity.

The E-value is the minimum strength of association, on the risk ratio scale, that an unmeasured confounder would need to have with both the exposure variable (S) and the outcome (Y) in order to fully explain away a specific observed exposure–outcome association, conditional on the measured covariates [VanderWeele and Ding (2017); VanderWeele and Mathur (2020)]. Here, in this section alone, we refer to the antibody marker S as an "exposure" variable following the typical set-up in the causal inference statistical methods literature. If, as in CoP analyses, the estimated marginalized risk ratio

$\widehat{RR}_M(s_1, s_2) = \widehat{r}_M(s_2)/\widehat{r}_M(s_1)$ for $s_1 < s_2$ is less than one, then the E-value for $\widehat{RR}_M(s_1, s_2)$ is calculated as

$$e_{RR}(s_1, s_2) = \frac{1 + \sqrt{1 - \widehat{RR}_M(s_1, s_2)}}{\widehat{RR}_M(s_1, s_2)} \ . \tag{3}$$

We include the argument (s_1, s_2) in the notation, with $s_1 < s_2$ by convention, to be clear that the E-value depends on specification of two specific marker-level subgroups.

To illustrate the interpretation of an E-value, suppose S is binary with levels 0 and 1 and regression analysis yields an estimate $\widehat{RR}_M(0, 1) = \widehat{r}_M(1)/\widehat{r}_M(0) = 0.40$ with 95% confidence interval (CI) $(0.14, 0.78)$. An E-value $e(0, 1)$ of 4.4 means that a marginalized risk ratio $RR_M(0, 1)$ at the observed value 0.40 could be explained away (i.e., $RR_C(0, 1) = 1.0$) by an unmeasured confounder associated with both the exposure and the outcome by a marginalized risk ratio of 4.4-fold each, after accounting for the vector X of measured confounders, but that weaker confounding could not do so.

In addition, we follow the recommendation of VanderWeele and Ding (2017) to also report the E-value $e_{UL}(s_1, s_2)$ for the upper limit $\widehat{UL}(s_1, s_2)$ of the 95% CI for the observed marginalized risk ratio $\widehat{RR}_M(s_1, s_2)$, computed as 1 if $\widehat{UL}(s_1, s_2) \geq 1$ and, otherwise, as

$$\frac{1 + \sqrt{1 - \widehat{UL}(s_1, s_2)}}{\widehat{UL}(s_1, s_2)} \ ,$$

which in the example equals $e_{UL}(0, 1) = 1.88$. This E-value for the upper limit indicates, for given $s_1 < s_2$, the strength of unmeasured confounding at which statistical significance of the inference that $RR_C(s_1, s_2) < 1$ would be lost. The two E-values above are useful for judging how confident we can be that an immunologic biomarker is a controlled risk CoP, with E-values near one suggesting weak support and evidence increasing with greater E-values.

Because $RR_C(s_1, s_2) = (1 - CVE(s_2))/(1 - CVE(s_1))$, evidence for $RR_C(s_1, s_2) < 1$ is equivalently evidence for $CVE(s_1) < CVE(s_2)$. Thus in

a placebo-controlled trial $RR_C(s_1, s_2)$ can be interpreted as the multiplicative degree of superior vaccine efficacy caused by marker level s_2 vs. marker level s_1, and E-values quantify evidence for whether $CVE(s_1)$ is less than $CVE(s_2)$. It is also useful to provide conservative estimates of controlled risk ratios and of the controlled risk curve, accounting for unmeasured confounding. We approach these tasks based on the sensitivity analysis, or bias analysis, approach of Ding and VanderWeele (2016). We give their main result and refer readers to the paper for details.

We begin by defining two (possibly context-specific) fixed sensitivity parameters. First, we set $RR_{UD}(s_1, s_2)$ to be the maximum risk ratio for the outcome Y comparing any two categories of the unmeasured confounders U, within either exposure group $S = s_1$ or $S = s_2$, conditional on the vector X of observed covariates. Second, we set $RR_{EU}(s_1, s_2)$ to be the maximum risk ratio for any specific level of the unmeasured confounder U comparing individuals with $S = s_1$ to those with $S = s_2$, with adjustment already made for the measured covariate vector X. Thus, $RR_{UD}(s_1, s_2)$ quantifies the importance of the unmeasured confounder U for the outcome, and $RR_{EU}(s_1, s_2)$ quantifies how imbalanced the exposure/marker subgroups $S = s_1$ and $S = s_2$ are in the unmeasured confounder U. The values $RR_{UD}(s_1, s_2)$ and $RR_{EU}(s_1, s_2)$ are always specified as greater than or equal to one. We suppose that $RR_M(s_1, s_2) < 1$ for the fixed values $s_1 < s_2$ — this is the case of interest for immune correlates.

Define the bias factor

$$B(s_1, s_2) = \frac{RR_{UD}(s_1, s_2) RR_{EU}(s_1, s_2)}{RR_{UD}(s_1, s_2) + RR_{EU}(s_1, s_2) - 1}$$

for $s_1 \leq s_2$, and define $RR_M^U(s_1, s_2)$ the same way as $RR_M(s_1, s_2)$, except marginalizing over the joint distribution of X and U. Then, $RR_M^U(s_1, s_2) \leq RR_M(s_1, s_2) \times B(s_1, s_2)$, where $RR_M^U(s_1, s_2) = E\{r(s_2, X^*)\}/E\{r(s_1, X^*)\}$ with $X^* = (X, U)$ and $r(s, x, u) = P(Y = 1 \,|\, S = s, A = 1, X = x, U = u)$ conditional risk. Translating this result to our problem context, under the positivity assumption, we have that $RR_M^U(s_1, s_2) = RR_C(s_1, s_2)$ and so, it

follows that

$$RR_C(s_1, s_2) \leq RR_M(s_1, s_2) \times B(s_1, s_2) \ . \tag{4}$$

This inequality states that the controlled risk ratio is bounded above by the marginalized risk ratio multiplied by the bias factor. It follows that a conservative (upper bound) estimate of $RR_C(s_1, s_2)$ is obtained as $\widehat{RR}_M(s_1, s_2) \times B(s_1, s_2)$, and a conservative 95% CI is obtained by multiplying each confidence limit for $RR_M(s_1, s_2)$ by $B(s_1, s_2)$. These estimates for $RR_C(s_1, s_2)$ account for the presumed-maximum plausible amount of deviation from the no unmeasured confounders assumption specified by $RR_{UD}(s_1, s_2)$ and $RR_{EU}(s_1, s_2)$. An appealing feature of this approach is that the bound (4) holds without making any assumption about the confounder vector X or the unmeasured confounder U.

Conservative (bounded) estimation of $r_C(s)$ *and* $RR_C(s_1, s_2)$ *for a quantitative marker* S The above approach does not directly provide a conservative estimate of the controlled risk curve $r_C(s)$, because additional information is needed for absolute versus relative risk estimation. To provide conservative inference for $r_C(s)$, we next select a central value s^{cent} of S such that $\widehat{r}_M(s^{cent})$ matches the observed overall risk, $\widehat{P}(Y = 1|A = 1)$. This value is a 'central' marker value at which the observed marginalized risk equals the observed overall risk. Next, we 'anchor' the analysis by assuming $r_C(s^{cent}) = r_M(s^{cent})$, where picking the central value s^{cent} makes this plausible to be at least approximately true. Under this assumption, the bound (4) implies the bounds

$$r_C(s) \ \leq \ r_M(s)B(s^{cent}, s) \quad \text{if } \ s \geq s^{cent} \tag{5}$$

$$r_C(s) \ \geq \ r_M(s)\frac{1}{B(s, s^{cent})} \quad \text{if } \ s < s^{cent}. \tag{6}$$

Therefore, after specifying $B(s^{cent}, s)$ and $B(s^{cent}, s)$ for all s, we conservatively estimate $r_c(s)$ by plugging $\widehat{r}_M(s)$ into the formulas (5) and (6).

Because $B(s_1, s_2)$ is always greater than one for $s_1 < s_2$, formula (5) pulls the observed risk $\widehat{r}_M(s)$ upwards for subgroups with high biomarker values, and formula (6) pulls the observed risk $\widehat{r}_M(s)$ downwards for subgroups with low

biomarker values. This makes the estimate of the controlled risk curve flatter, closer to the null curve, as desired for a sensitivity/robustness analysis.

To specify $B(s_1, s_2)$, we note that it should have greater magnitude for a greater distance of s_1 from s_2, as determined by specifying $RR_{UD}(s_1, s_2)$ and $RR_{EU}(s_1, s_2)$ increasing with $s_2 - s_1$ (for $s_1 \leq s_2$). We consider one specific approach, which sets $RR_{UD}(s_1, s_2) = RR_{EU}(s_1, s_2)$ to the common value $RR_U(s_1, s_2)$ that is specified log-linearly: $\log RR_U(s_1, s_2) = \gamma(s_2 - s_1)$ for $s_1 \leq s_2$. Then, for a user-selected pair of values $s_1 = s_1^{fix}$ and $s_2 = s_2^{fix}$ with $s_1^{fix} < s_2^{fix}$, we set a sensitivity parameter $RR_U(s_1^{fix}, s_2^{fix})$ to some value above one. It follows that

$$\log RR_U(s_1, s_2) = \left(\frac{s_2 - s_1}{s_2^{fix} - s_1^{fix}}\right) \log RR_U(s_1^{fix}, s_2^{fix}), \quad s_1 \leq s_2.$$

We anchor the analysis by setting $s_1 = s_1^{fix}$ at the 15th percentile of the Day 35 antibody marker and $s_2 = s_2^{fix}$ at the 85th percentile of the Day 35 antibody marker.

Once $r_C(s)$ is conservatively estimated via the formulas (5) and (6), it is immediate how to obtain a conservative estimate of $\text{CVE}(s)$:

$$\widehat{\text{CVE}}(s) = 1 - \frac{\widehat{r}_M(s) B(s^{cent}, s)}{\widehat{P}(Y(0) = 1)},$$

where the estimate of the placebo arm risk, $\widehat{P}(Y(0) = 1)$, is the same as for the controlled VE analysis assuming no-unmeasured confounders.

Sensitivity analyses for controlled vaccine efficacy reported in the article

The sensitivity analysis is done for each of the two Cox model CoR analyses described in Section 9.3.2, first for the binary Day 35 marker and second for the quantitative Day 35 marker. For the former analysis, E-values are reported for both the point estimate and the upper 95% confidence limit for $RR_C(0, 1)$, where category 1 is the upper tertile (vaccine recipients with antibodies S in the top third), category 0 is the lower tertile (vaccine recipients with antibodies in the bottom third), and the intermediate middle tertile subgroup of vaccine recipients is excluded from the analysis. In

addition, we set $RR_{UD}(0,1) = RR_{EU}(0,1) = 2$, such that $B(0,1) = 4/3$, and report conservative estimation and inference on the controlled risk ratio $RR_C(0,1)$ and equivalently on the ratio of controlled vaccine efficacy curves $RR_C(0,1) = (1 - CVE(1))/(1 - CVE(0))$.

Next, we conduct the sensitivity analysis treating S as a quantitative variable, as detailed in the subsection above "*Conservative (bounded) estimation of* $r_C(s)$ *and* $RR_C(s_1, s_2)$ *for a quantitative marker* S." This analysis reports results in terms of point and 95% point-wise confidence interval estimates of CVE(s) vs. s assuming the specified amount of unmeasured confounding that makes the estimates of CVE(s) flatter than under the assumption of no unmeasured confounding.

For validity the controlled risk/vaccine efficacy analyses require the positivity assumption, and thus the methods will only be applied if the data are reasonably supportive of the positivity assumption. To check positivity, we study the antibody marker distribution in vaccine recipients within each subgroup of the covariates X that are adjusted for. For the tertiles analysis we require evidence that within each subgroup some vaccine recipients have lower tertile responses and some vaccine recipients have upper tertile responses. For the quantitative S analysis, we look for evidence that S varies over its full range within each level of the potential confounders that are adjusted for.

The details of the above causal sensitivity analysis are described in the in-press article (Gilbert et al., 2022).

12 Estimating a Threshold of Protection Based on an Established or Putative CoP (Population-Based CoP)

For each antibody marker studied as a CoP, we will apply the Chang-Kohberger (2003) / Siber (2007) method to estimate a threshold of the antibody marker associated with the estimate of overall vaccine efficacy observed in the trial.

This method makes two simplifying assumptions: (1) that a high enough antibody marker value s^* implies that individuals with $S > s^*$ have essentially zero disease risk (perfect protection) regardless of whether they were vacci-

nated; and (2) $P(Y = 1|S \leq s^*, A = 1)/P(Y = 1|S \leq s^*, A = 0) = 1$ (zero vaccine efficacy if $S \leq s^*$). Based on these assumptions, s^* is calculated as the value equating $1 - \widehat{P}(S \leq s^*|A = 1)/\widehat{P}(S \leq s^*|A = 0)$ to the estimate of overall vaccine efficacy. This estimate is supplemented by estimating the reverse cumulative distribution function (RCDF) of S in baseline negative vaccine recipients and calculating a 95% confidence interval for the threshold value s^* as the points of intersection of the estimated RCDF curve with the 95% confidence interval for overall vaccine efficacy (as in the figure in Andrews and Goldblatt, 2014).

This method essentially assumes that S has already been established as a CoP, and under that assumption estimates a threshold that may be considered as a benchmark / study endpoint for future immunogenicity vaccine trial applications.

It is acknowledged that this approach makes simplifying assumptions that are diagnosed to be violated in the PREVENT-19 trial; nonetheless it may yield a useful benchmark and complementary information on a threshold correlate of protection.

13 Considerations for Baseline SARS-CoV-2 Positive Study Participants

As stated above, if enough COVID cases in baseline positive vaccine and/or placebo recipients occur, then additional correlates analyses may be planned in baseline positive individuals. For example, the same or similar correlates of risk analysis plan that is used to analyze Day 35 marker correlates of risk in baseline negative vaccine recipients could be applied to assess Day 1 marker correlates of risk in baseline positive placebo recipients. In addition, analyses could be done to assess how vaccine efficacy in baseline positive participants varies with Day 1 markers. It is straightforward to make this analysis rigorous because Day 1 markers are a baseline covariate, such that regression analyses are valid based on the randomization.

14 Avoiding Bias with Pseudovirus Neutralization Analysis due to Use of Anti-HIV Antiretroviral Drugs

Because the lentivirus-based pseudovirus neutralization assay uses an HIV backbone, the presence of anti-retroviral drugs in serum will give a false positive neutralization signal. This can be easily screened for using an MuLV pseudotype control. Therefore, Day 1 and Day 35 samples of all study participants with data included in correlates analyses will be tested for presence of anti-retroviral drugs. Samples with the control indicating likely anti-retroviral are excluded from analyses, for all analyses that include pseudovirus neutralization. Analyses that do not consider pseudovirus neutralization are unaffected by this issue.

A

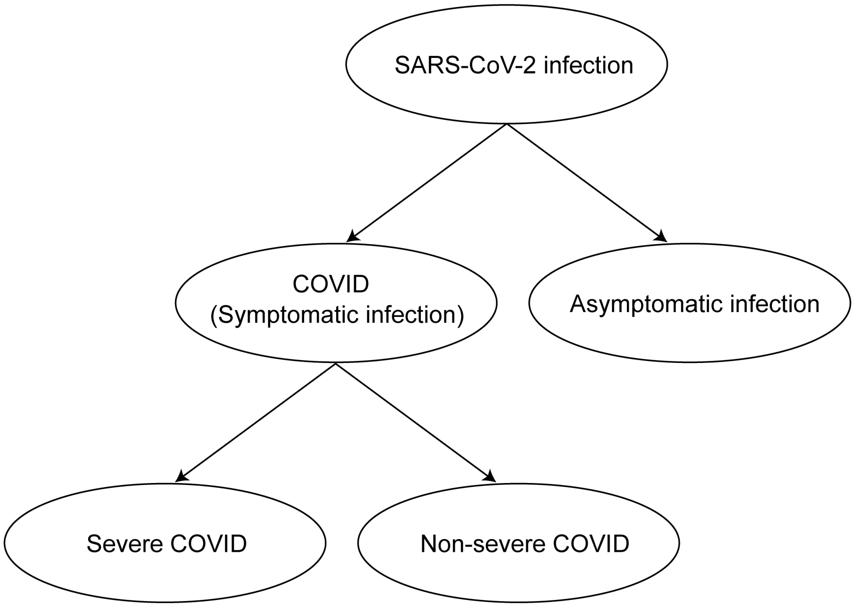

B

**Clinical Endpoint**	**Definition**
SARS-CoV-2 infection	Positive RNA PCR test or SARS-CoV-2 seroconversion*, whichever occurs first
COVID (Symptomatic infection)	Meeting a protocol-specified list of COVID-19 symptoms with virological confirmation of SARS-CoV-2 infection (symptom triggered)
Asymptomatic infection	SARS-CoV-2 seroconversion* without prior diagnosis of the COVID endpoint¶
Severe COVID	COVID endpoint with at least one protocol-specified severe disease event
Non-severe COVID	COVID endpoint with zero protocol-specified severe disease events

*Seroconversion is assessed via a validated assay that distinguishes natural vs vaccine-induced SARS-CoV-2 antibodies

¶ Alternatively, the asymptomatic infection endpoint can also include an RNA PCR+ test result obtained through testing regardless of symptoms (e.g., as a requirement for travel, return to school or work, or elective medical procedures) and follow-up to confirm the participant remains asymptomatic

Figure 2: A) Structural relationships among study endpoints in a COVID-19 vaccine efficacy trial (Mehrotra et al., 2020). B) Study endpoint definitions.

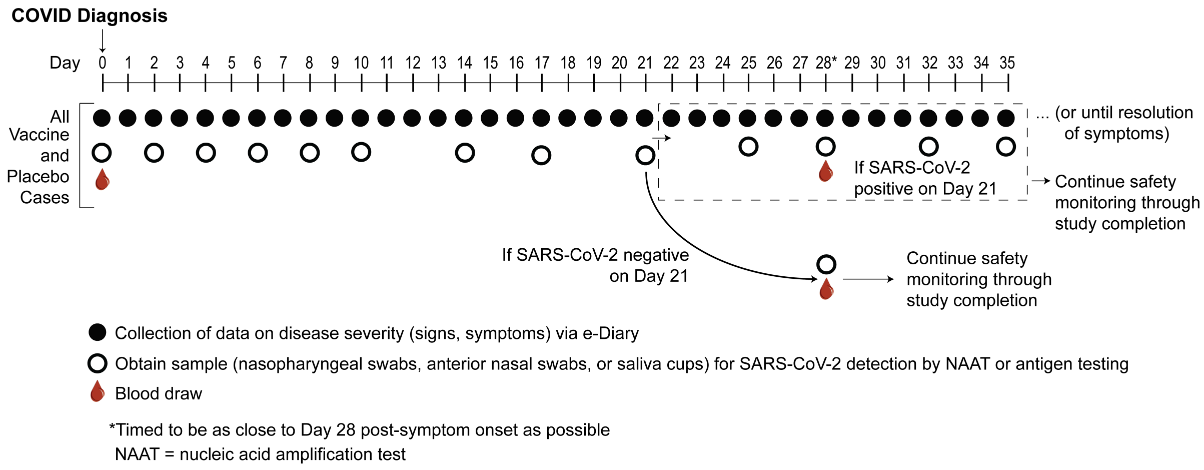

Figure 3: Example at-COVID diagnosis and post-COVID diagnosis disease severity and virologic sampling schedule, in a setting where frequent follow-up of confirmed cases can be assured. Participants diagnosed with virologically-confirmed symptomatic SARS-CoV-2 infection (COVID) enter a post-diagnosis sampling schedule to monitor viral load and COVID-related symptoms (types, severity levels, and durations).

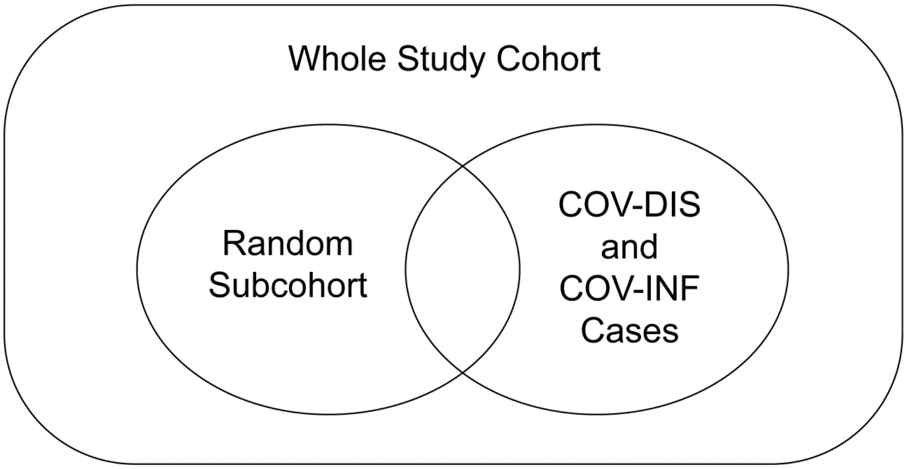

Figure 4: Case-cohort sampling design (Prentice, 1986) that measures Day 1, Day 35 antibody markers in all participants selected into the subcohort and in all COVID and COV-INF cases occurring outside of the subcohort.

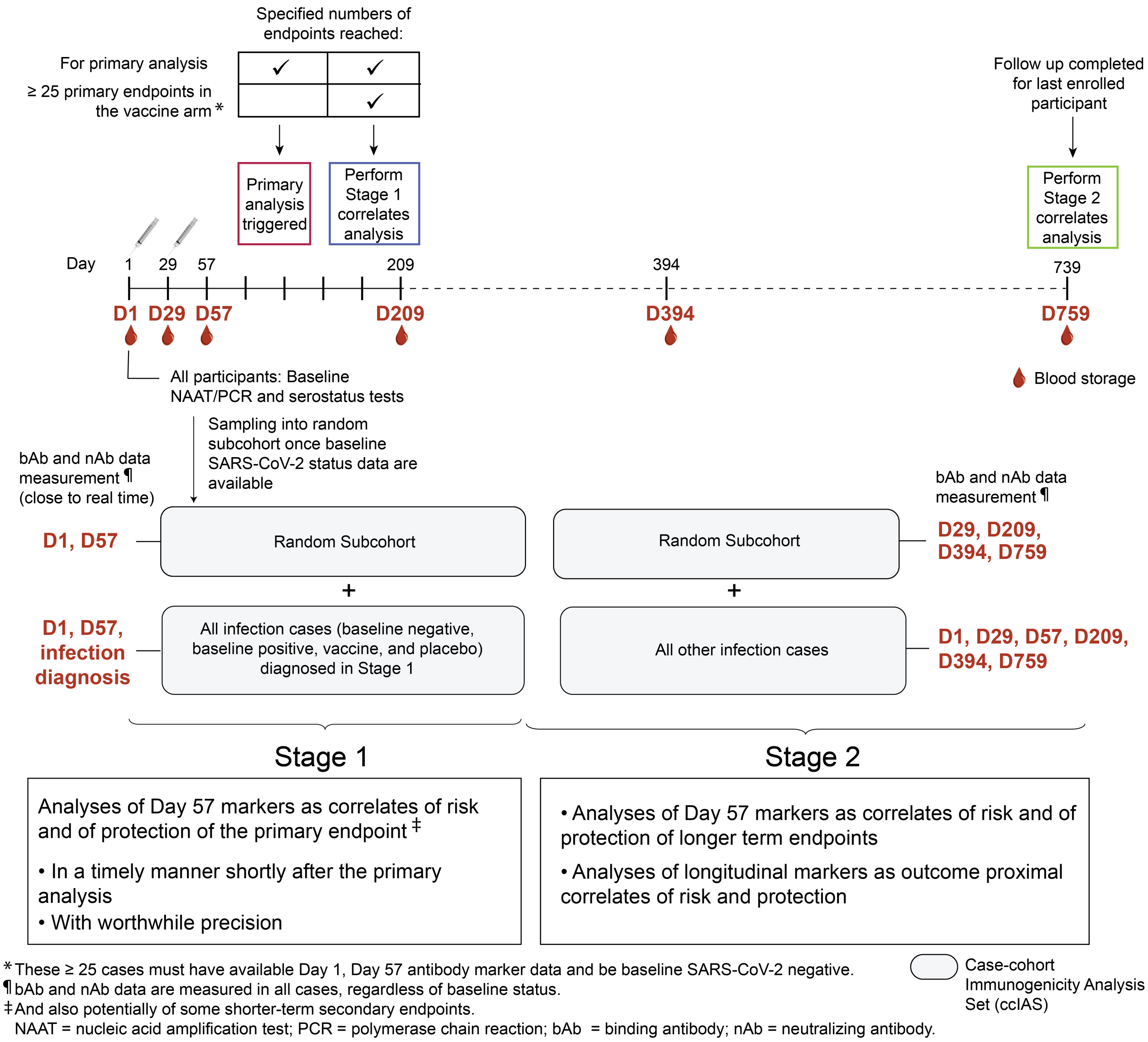

Figure 5: Two-stage correlates analysis. Stage 1 consists of analyses of Day 35 markers as correlates of risk and of protection of the primary endpoint and potentially also of some secondary endpoints, and includes antibody marker data from all COVID and SARS-CoV-2 infection cases (COV-INF) through to the time of the data lock for the first correlates analyses. Stage 2 consists of analyses of Day 35 markers as correlates of risk and of protection of longer term endpoints and analyses of longitudinal markers as outcome-proximal correlates of risk and of protection, and includes antibody marker data from all subsequent COVID and COV-INF cases. Stage 1 measures Day 1, Day 35 antibody markers and COV-INF and COVID diagnosis time point markers; Stage 2 measures antibody markers from all sampling time points and COV-INF plus COVID diagnosis sampling time points not yet assayed. The same immunogenicity subcohort is used for both stages.

References

Andrews, N.J., Waight, P.A., Burbidge, P., Pearce, E., Roalfe, L., Zancolli, M. et al (2014), "Serotype-specific effectiveness and correlates of protection for the 13-valent pneumococcal conjugate vaccine: a postlicensure indirect cohort study," *The Lancet infectious diseases*, 14, 839–846.

Breslow, N., Lumley, T., Ballantyne, C., Chambless, L. and Kulich, M. (2009a), "Improved Horvitz-Thompson Estimation of Model Parameters from Two-phase Stratified Samples: Applications in Epidemiology," *Statistical Biosciences*, 1, 32–49.

Breslow, N., Lumley, T., Ballantyne, C., Chambless, L. and Kulich, M. (2009b), "Using the whole cohort in the analysis of case-cohort data." *American Journal of Epidemiology*, 169, 1398–1405.

Cowling, B., Lim, W., Perera, R., Fang, V., Leung, G., Peiris, J. et al (2019), "Influenza hemagglutination-inhibition antibody titer as a mediator of vaccine-induced protection for influenza B," *Clinical Infectious Diseases*, 68(10), 1713–7.

Ding, P. and VanderWeele, T. (2016), "Sensitivity analysis without assumptions," *Epidemiology*, 27(3), 368.

Donovan, K., Hudgens, M. and Gilbert, P.B. (2019), "Nonparametric inference for immune response thresholds of risk in vaccine studies," *Annals of Applied Statistics*, 13, 1147–1165, PMCID: PMC6613658 [Delayed release (embargo): Available on 2020-06-01].

Feng, S., Phillips, D.J., White, T., Sayal, H., Aley, P.K., Bibi, S. et al (2021), "Correlates of protection against symptomatic and asymptomatic SARS-CoV-2 infection," *Nature medicine*, pp. 1–9.

Fleming, T.R. and Powers, J.H. (2012), "Biomarkers and surrogate endpoints in clinical trials," *Statistics in Medicine*, 31, 2973–2984.

Gilbert, P., Fong, Y., Kenny, A. and Carone, M. (2022), "A Controlled Effects Approach to Assessing Immune Correlates of Protection," *Biostatistics*, under review.

Gilbert, P.B., Montefiori, D.C., McDermott, A.B., Fong, Y., Benkeser, D., Deng, W. et al (2021), “Immune correlates analysis of the mRNA-1273 COVID-19 vaccine efficacy clinical trial,” *Science*, p. eab3435.

Hejazi, N.S., van der Laan, M.J., Janes, H.E. and Benkeser, D.C. (2020), “Efficient nonparametric inference on the effects of stochastic interventions under two-phase sampling, with applications to vaccine efficacy trials,” *Biometrics*, Ahead of print.

Hubbard, A.E., Khered-Pajouh, S. and van der Laan, M.J. (2016), “Statistical inference for data adaptive target parameters,” *The International Journal of Biostatistics*, 12, 3–19.

Jodar, L., Butler, J., Carlone, G., Dagan, R., Goldblatt, D., Kďż″yhty, H. et al (2003), “Serological criteria for evaluation and licensure of new pneumococcal conjugate vaccine formulations for use in infants.” *Vaccine*, 21, 3265–3272.

Li, C. and Shepherd, B.E. (2012), “A new residual for ordinal outcomes,” *Biometrika*, 99, 473–480.

Magaret, C., Benkeser, D., Williamson, B., Borate, B., Carpp, L., Georgiev, I. et al (2019), “Prediction of VRC01 neutralization sensitivity by HIV-1 gp160 sequence features.” *PLoS Computational Biology*, 15, e1006952, PMCID: PMC6459550.

McCallum, M., Walls, A.C., Bowen, J.E., Corti, D. and Veesler, D. (2020), “Structure-guided covalent stabilization of coronavirus spike glycoprotein trimers in the closed conformation,” *Nature structural & molecular biology*, 27, 942–949.

Mehrotra, D.V., Janes, H.E., Fleming, T.R., Annunziato, P.W., Neuzil, K.M., Carpp, L.N. et al (2020), “Clinical Endpoints for Evaluating Efficacy in COVID-19 Vaccine Trials,” *Annals of Internal Medicine*.

Moodie, Z., Juraska, M., Huang, Y., Zhuang, Y., Fong, Y., Carpp, L.N. et al (2018), “Neutralizing antibody correlates analysis of tetravalent dengue

vaccine efficacy trials in Asia and Latin America." *Journal of Infectious Diseases*, 217(5), 742–753.

Neidich, S.D., Fong, Y., Li, S.S., Geraghty, D.E., Williamson, B.D., Young, W.C. et al (2019), "Antibody Fc effector functions and IgG3 associate with decreased HIV-1 risk," *Journal of Clinical Investigation*, 129, 4838–4849.

Newcombe, R. (1998), "Interval Estimation for the Difference Between Independent Proportions: Comparison of Eleven Methods," *Statistics in Medicine*, 17, 873–90.

Prentice, R. (1986), "A case-cohort design for epidemiologic cohort studies and disease prevention trials." *Biometrika*, 73, 1–11.

Sholukh, A.M., Fiore-Gartland, A., Ford, E.S., Hou, Y., Tse, L.V., Lempp, F.A. et al (2020), "Evaluation of SARS-CoV-2 neutralization assays for antibody monitoring in natural infection and vaccine trials," *medRxiv*.

Siber, G., Chang, I., Baker, S., Fernsten, P., O'Brien, K., Santosham, M. et al (2007), "Estimating the protective concentration of anti-pneumococcal capsular polysaccharide antibodies." *Vaccine*, 25, 3816–3826.

van der Laan, L., Zhang, W. and Gilbert, P.B. (2022), "Efficient nonparametric estimation of the covariate-adjusted threshold-response function, a support-restricted stochastic intervention." *Biometrics*, in press.

VanderWeele, T. (2013), "Surrogate measures and consistent surrogates," *Biometrics*, 69, 561–568.

VanderWeele, T. and Ding, P. (2017), "Sensitivity analysis in observational research: introducing the E-value," *Annals of Internal Medicine*, 167(4), 268–74.

VanderWeele, T. and Mathur, M. (2020), "Commentary: developing best-practice guidelines for the reporting of E-values," *International Journal of Epidemiology*, Aug 2.

Westfall, P.H., Young, S.S. et al (1993), *Resampling-based multiple testing: Examples and methods for p-value adjustment*, vol. 279, John Wiley & Sons.

Williamson, B., Gilbert, P., Simon, N. and Carone, M. (2022), "A general framework for inference on algorithm-agnostic variable importance," *JASA*.

15 Novavax Binary Principal Stratification Results

list of variables to include in Baseline behavior risk

Table 5: Novavax PREVENT-19: Correlates of Vaccine Efficacy Results by Gilbert et al. (2020) Method for High vs. Low Marker Subgroups Under No Early Harm Assumption with Sensitivity Analysis Scenarios*

	Marker	Sens	Ilol	Ilou	Elol	Elou	Ihil	Ihiu	Ehil	Ehiu	Icnl	Icnu	Ecnl	Ecnu
1	D57S.4	None	0.79	0.79	0.64	0.88	0.95	0.95	0.92	0.97	4.22	4.22	2.17	8.21
2	D57S.4	Med	0.72	0.84	0.56	0.90	0.95	0.95	0.92	0.97	3.05	5.80	1.73	10.05
3	D57S.4	High	0.58	0.89	0.34	0.94	0.94	0.95	0.92	0.97	1.89	8.99	1.04	15.49
4	D57RBD.4	None	0.77	0.77	0.60	0.86	0.95	0.95	0.93	0.97	4.68	4.68	2.41	9.11
5	D57RBD.4	Med	0.69	0.83	0.52	0.89	0.95	0.95	0.93	0.97	3.38	6.42	1.91	11.14
6	D57RBD.4	High	0.53	0.89	0.28	0.93	0.95	0.95	0.92	0.97	2.09	9.97	1.15	17.18
7	D57ID50.4	None	0.75	0.75	0.58	0.84	0.88	0.88	0.80	0.93	2.12	2.12	1.06	4.24
8	D57ID50.4	Med	0.66	0.81	0.48	0.88	0.87	0.89	0.80	0.93	1.46	3.03	0.81	5.40
9	D57ID50.4	High	0.48	0.88	0.22	0.92	0.85	0.90	0.77	0.93	0.83	4.97	0.45	9.13
10	D57ID80.4	None	0.73	0.73	0.55	0.84	0.89	0.89	0.81	0.93	2.35	2.35	1.16	4.74
11	D57ID80.4	Med	0.64	0.80	0.45	0.87	0.88	0.89	0.81	0.93	1.62	3.36	0.89	6.04
12	D57ID80.4	High	0.45	0.87	0.16	0.92	0.86	0.90	0.78	0.94	0.93	5.51	0.49	10.24
13	D57S.5	None	0.87	0.87	0.79	0.92	0.95	0.95	0.93	0.97	2.87	2.87	1.52	5.42
14	D57S.5	Med	0.83	0.90	0.75	0.93	0.95	0.96	0.92	0.97	2.07	3.93	1.21	6.68
15	D57S.5	High	0.76	0.93	0.65	0.95	0.94	0.96	0.91	0.97	1.28	6.08	0.74	10.31
16	D57RBD.5	None	0.87	0.87	0.80	0.92	0.95	0.95	0.92	0.97	2.65	2.65	1.40	5.00
17	D57RBD.5	Med	0.84	0.90	0.76	0.93	0.95	0.96	0.92	0.97	1.91	3.63	1.12	6.17
18	D57RBD.5	High	0.77	0.93	0.66	0.95	0.94	0.96	0.91	0.98	1.18	5.72	0.68	10.51
19	D57ID50.5	None	0.75	0.75	0.62	0.83	0.91	0.91	0.82	0.95	2.72	2.72	1.26	5.84
20	D57ID50.5	Med	0.67	0.81	0.54	0.86	0.90	0.92	0.82	0.95	1.87	3.88	0.98	7.36
21	D57ID50.5	High	0.53	0.87	0.33	0.91	0.87	0.93	0.78	0.96	1.06	6.34	0.54	12.19
22	D57ID80.5	None	0.78	0.78	0.65	0.86	0.88	0.88	0.80	0.93	1.90	1.90	0.93	3.87
23	D57ID80.5	Med	0.72	0.83	0.58	0.89	0.87	0.90	0.79	0.94	1.31	2.72	0.72	4.93
24	D57ID80.5	High	0.59	0.88	0.40	0.92	0.84	0.91	0.74	0.94	0.74	4.43	0.39	8.16
25	D57S.6	None	0.88	0.88	0.82	0.92	0.97	0.97	0.94	0.98	3.82	3.82	1.87	7.80
26	D57S.6	Med	0.85	0.90	0.80	0.93	0.96	0.97	0.94	0.98	2.76	5.24	1.51	9.51
27	D57S.6	High	0.80	0.92	0.72	0.95	0.96	0.97	0.93	0.98	1.70	8.07	0.93	14.62
28	D57RBD.6	None	0.88	0.88	0.83	0.92	0.97	0.97	0.94	0.98	3.42	3.42	1.71	6.84
29	D57RBD.6	Med	0.85	0.90	0.80	0.93	0.96	0.97	0.93	0.98	2.47	4.68	1.37	8.36
30	D57RBD.6	High	0.80	0.93	0.73	0.95	0.95	0.97	0.92	0.98	1.52	7.22	0.84	12.86
31	D57ID50.6	None	0.78	0.78	0.67	0.85	0.92	0.92	0.81	0.96	2.65	2.65	1.08	6.47
32	D57ID50.6	Med	0.72	0.82	0.62	0.87	0.90	0.93	0.80	0.96	1.82	3.78	0.86	8.00
33	D57ID50.6	High	0.62	0.87	0.47	0.91	0.87	0.94	0.75	0.97	1.03	6.17	0.47	13.30
34	D57ID80.6	None	0.79	0.79	0.69	0.86	0.90	0.90	0.80	0.95	2.07	2.07	0.91	4.69
35	D57ID80.6	Med	0.74	0.84	0.64	0.88	0.88	0.91	0.78	0.95	1.42	2.95	0.71	5.86
36	D57ID80.6	High	0.64	0.88	0.50	0.92	0.85	0.93	0.72	0.96	0.81	4.82	0.39	9.77

*None: beta sensitivity parameters log(1.0) and -log(1.0); Med: beta sensitivity parameters log(0.75) and -log(0.75); High: beta sensitivity parameters log(0.5) and -log(0.5)

Table 6: Novavax PREVENT-19: Correlates of Vaccine Efficacy Results by Gilbert et al. (2020) Method for High vs. Low Marker Subgroups Under No Early Harm Assumption with Sensitivity Analysis Scenarios*

	Marker	Sens	Ilol	Ilou	Elol	Elou	Ihil	Ihiu	Ehil	Ehiu	Icnl	Icnu	Ecnl	Ecnu
1	D29S.3	None	0.78	0.78	0.54	0.89	0.94	0.94	0.92	0.96	3.62	3.62	1.63	8.04
2	D29S.3	Med	0.70	0.84	0.45	0.92	0.94	0.94	0.92	0.96	2.61	4.97	1.30	9.62
3	D29S.3	High	0.54	0.90	0.17	0.95	0.94	0.94	0.91	0.96	1.62	7.72	0.77	14.75
4	D29RBD.3	None	0.85	0.85	0.65	0.93	0.94	0.94	0.91	0.95	2.36	2.36	0.97	5.72
5	D29RBD.3	Med	0.79	0.89	0.59	0.95	0.93	0.94	0.91	0.95	1.70	3.24	0.79	6.78
6	D29RBD.3	High	0.69	0.93	0.38	0.97	0.93	0.94	0.91	0.95	1.05	5.04	0.47	10.40
7	D29ID50.3	None	0.58	0.58	0.34	0.73	0.90	0.90	0.84	0.94	4.23	4.23	2.25	7.95
8	D29ID50.3	Med	0.43	0.69	0.17	0.79	0.89	0.91	0.84	0.94	2.92	6.05	1.71	10.24
9	D29ID50.3	High	0.13	0.80	-0.27	0.87	0.88	0.91	0.83	0.94	1.67	9.82	0.97	16.58
10	D29S.4	None	0.86	0.86	0.76	0.91	0.95	0.95	0.92	0.96	2.60	2.60	1.40	4.83
11	D29S.4	Med	0.81	0.89	0.71	0.93	0.94	0.95	0.92	0.96	1.88	3.58	1.11	5.97
12	D29S.4	High	0.72	0.93	0.57	0.95	0.94	0.95	0.91	0.96	1.16	5.54	0.67	9.23
13	D29RBD.4	None	0.87	0.87	0.78	0.92	0.94	0.94	0.92	0.96	2.29	2.29	1.23	4.29
14	D29RBD.4	Med	0.83	0.90	0.73	0.94	0.94	0.95	0.92	0.96	1.65	3.15	0.97	5.31
15	D29RBD.4	High	0.74	0.93	0.61	0.96	0.93	0.95	0.91	0.96	1.02	4.89	0.59	8.20
16	D29ID50.4	None	0.66	0.66	0.49	0.77	0.91	0.91	0.85	0.95	3.88	3.88	2.01	7.47
17	D29ID50.4	Med	0.55	0.74	0.37	0.82	0.90	0.92	0.85	0.95	2.67	5.54	1.53	9.60
18	D29ID50.4	High	0.33	0.83	0.07	0.88	0.89	0.93	0.82	0.95	1.53	8.98	0.87	15.53
19	D29S.5	None	0.87	0.87	0.81	0.92	0.95	0.95	0.93	0.97	2.72	2.72	1.52	4.86
20	D29S.5	Med	0.84	0.90	0.77	0.93	0.95	0.96	0.93	0.97	1.96	3.74	1.20	6.06
21	D29S.5	High	0.77	0.93	0.68	0.95	0.94	0.96	0.92	0.97	1.21	5.78	0.73	9.36
22	D29RBD.5	None	0.88	0.88	0.81	0.92	0.95	0.95	0.93	0.97	2.68	2.68	1.50	4.78
23	D29RBD.5	Med	0.84	0.90	0.78	0.93	0.95	0.96	0.93	0.97	1.93	3.67	1.18	5.96
24	D29RBD.5	High	0.77	0.93	0.68	0.95	0.94	0.96	0.92	0.97	1.19	5.68	0.72	9.20
25	D29ID50.5	None	0.75	0.75	0.63	0.83	0.90	0.90	0.83	0.94	2.62	2.62	1.33	5.17
26	D29ID50.5	Med	0.68	0.80	0.55	0.86	0.89	0.91	0.82	0.95	1.81	3.75	1.02	6.62
27	D29ID50.5	High	0.54	0.86	0.36	0.90	0.86	0.92	0.78	0.96	1.03	6.11	0.58	11.56
28	D29S.6	None	0.89	0.89	0.84	0.92	0.97	0.97	0.94	0.98	3.29	3.29	1.74	6.23
29	D29S.6	Med	0.86	0.91	0.81	0.93	0.96	0.97	0.94	0.98	2.37	4.52	1.39	7.70
30	D29S.6	High	0.81	0.93	0.74	0.95	0.95	0.97	0.92	0.98	1.46	6.97	0.84	11.87
31	D29RBD.6	None	0.89	0.89	0.84	0.92	0.96	0.96	0.94	0.98	3.01	3.01	1.61	5.62
32	D29RBD.6	Med	0.86	0.91	0.82	0.93	0.96	0.97	0.93	0.98	2.17	4.13	1.28	6.96
33	D29RBD.6	High	0.81	0.93	0.75	0.95	0.95	0.97	0.92	0.98	1.33	6.37	0.78	10.73

*None: beta sensitivity parameters log(1.0) and -log(1.0); Med: beta sensitivity parameters log(0.75) and -log(0.75); High: beta sensitivity parameters log(0.5) and -log(0.5)

Table 7: Novavax PREVENT-19: Cut-points Defining High and Low Marker Subgroups

	Perc. 0.4	Perc. 0.5	Perc. 0.6
D57Spike	1380.38	2094.11	2851.67
D57RBD	1895.40	3087.45	4218.91
D57ID50	157.98	223.46	297.17
D57ID80	381.24	497.62	606.18

Table 8: Novavax PREVENT-19: Cut-points Defining High and Low Marker Subgroups

	Perc. 0.3	Perc. 0.4	Perc. 0.5
D29Spike	55.89	140.02	225.48
D29RBD	52.35	139.38	225.79
D29ID50	5.71	8.67	13.76
D29ID80	16.43	21.77	27.56

Table 9: Novavax PREVENT-19: Cut-points Defining High and Low Marker Subgroups

	Perc. 0.3	Perc. 0.4	Perc. 0.5
D29Spike	1.75	2.15	2.35
D29RBD	1.72	2.14	2.35
D29ID50	0.76	0.94	1.14
D29ID80	1.22	1.34	1.44
