# Supplementary material for: Immune correlates analysis of the PREVENT-19 COVID-19 vaccine efficacy clinical trial
Source: Nat Commun. 2023 Jan 19;14:331. doi: 10.1038/s41467-022-35768-3 (PMC9851580; doi:10.1038/s41467-022-35768-3)
Supplement: Supplementary file 3 — Reporting Summary [file 41467_2022_35768_MOESM3_ESM.pdf]

nature portfolio

Corresponding author(s): Peter B. Gilbert

Last updated by author(s): Oct 31, 2022

Reporting Summary

Nature Portfolio wishes to improve the reproducibility of the work that we publish. This form provides structure for consistency and transparency in reporting. For further information on Nature Portfolio policies, see our Editorial Policies and the Editorial Policy Checklist.

Statistics

For all statistical analyses, confirm that the following items are present in the figure legend, table legend, main text, or Methods section.

n/a	Confirmed	
☐	☒	The exact sample size (n) for each experimental group/condition, given as a discrete number and unit of measurement
☐	☒	A statement on whether measurements were taken from distinct samples or whether the same sample was measured repeatedly
☐	☒	The statistical test(s) used AND whether they are one- or two-sided<br>*Only common tests should be described solely by name; describe more complex techniques in the Methods section.*
☐	☒	A description of all covariates tested
☐	☒	A description of any assumptions or corrections, such as tests of normality and adjustment for multiple comparisons
☐	☒	A full description of the statistical parameters including central tendency (e.g. means) or other basic estimates (e.g. regression coefficient) AND variation (e.g. standard deviation) or associated estimates of uncertainty (e.g. confidence intervals)
☐	☒	For null hypothesis testing, the test statistic (e.g. F, t, r) with confidence intervals, effect sizes, degrees of freedom and P value noted<br>*Give P values as exact values whenever suitable.*
☒	☐	For Bayesian analysis, information on the choice of priors and Markov chain Monte Carlo settings
☒	☐	For hierarchical and complex designs, identification of the appropriate level for tests and full reporting of outcomes
☐	☒	Estimates of effect sizes (e.g. Cohen's d, Pearson's r), indicating how they were calculated

Our web collection on statistics for biologists contains articles on many of the points above.

Software and code

Policy information about availability of computer code

Data collection	For the SARS CoV-2 Spike, RBD and Nucleocapsid assay: The MSD MESO Sector S 600 detection system quantitates the amount of light emitted and reports the ECL unit response as a result for each test sample, control sample and reference standard of each plate. The system software (MSD Discovery Workbench) is proprietary to MSD: https://www.mesoscale.com/en/products_and_services/software.
Data analysis	For the SARS CoV-2 Spike, RBD and Nucleocapsid assay: Data analysis is performed with the Molecular Devices software, SoftMaxPro GxP, Version 6.5.1.<br>For the correlates analyses: The analysis was implemented in R version 4.0.3; code was verified using mock data.<br>All analyses were done reproducibly based on publicly available R scripts hosted on the GitHub collaborative programming platform (https://github.com/CoVPN/correlates_reporting2).

For manuscripts utilizing custom algorithms or software that are central to the research but not yet described in published literature, software must be made available to editors and reviewers. We strongly encourage code deposition in a community repository (e.g. GitHub). See the Nature Portfolio guidelines for submitting code & software for further information.

Data

Policy information about availability of data

All manuscripts must include a data availability statement. This statement should provide the following information, where applicable:

- Accession codes, unique identifiers, or web links for publicly available datasets
- A description of any restrictions on data availability
- For clinical datasets or third party data, please ensure that the statement adheres to our policy

The Data will be posted on clinicaltrials.gov (https://clinicaltrials.gov/ct2/show/NCT04611802) when the trial is completed. Source data files for each figure are provided with this paper.

Human research participants

Policy information about studies involving human research participants and Sex and Gender in Research.

Reporting on sex and gender	Information on participant sex distribution in the US cohort is provided in Supplementary Table 2 of the manuscript.<br>Table 2 in the Statistical Analysis Plan describes baseline subgroups that were included in analyses of the immunogenicity data (e.g. immunogenicity tables by subgroup for baseline-negative and for baseline-positive individuals). Sex (male, female; where "sex" refers to sex assigned at birth, as self-reported), and age x sex (18-64 male, 18-64 female, >=65 female, >=65 male) were among these baseline subgroups. However, these tables are not included in the present article. The SAP specified reporting comparing baseline negative vaccine vs. baseline negative placebo and baseline positive vaccine vs. baseline positive placebo for comparing antibody levels between groups.<br>In addition, sex was included as a baseline covariate potentially relevant for SARS-CoV-2 exposure and risk of COVID in development of the baseline risk score (see section 7 of the SAP).<br>Data are not reported disaggregated for sex because the individual-level data cannot be shared as the trial is ongoing.<br>Sex-based correlates analyses were not performed due to the low number of primary endpoints across both sexes (12 vaccine recipient breakthrough COVID cases), which would reduce the statistical power for such analyses to such an extent that the conclusions would likely not be meaningful.
Population characteristics	The demographic and clinical characteristics of the US cohort are provided in Supplementary Table 2 of the manuscript.
Recruitment	The recruitment strategy was described in the primary paper, Dunkle et al. 2022 NEJM. As stated in the Supplementary Appendix of Dunkle et al., "At least 25% of the study population was originally intended to be in the ≥65 years age group; however, availability of vaccines under the Emergency Use Authorization (EUA) during the first weeks of the study required the reprioritization of this population for public health and ethical reasons. Prioritization was given to enrollment of individuals at overall high risk for Covid-19, e.g., high risk for acquisition of Covid-19 of any severity due to living circumstances common to the Black/African American or American Indian/Alaska Native communities (including Native Americans of Mexican origin), Hispanic of Latino ethnicity, or other living or working conditions involving known frequent exposure to SARS-CoV-2 (e.g., factory or meat packing plants, essential retail workers, etc.), or at high risk of developing severe Covid-19 complications by virtue of comorbid conditions (e.g., obesity [BMI >30 kg/m2], chronic kidney or lung disease, cardiovascular disease, and diabetes mellitus type 2).<br>The fact that the trial was a randomized trial, with careful allocation concealment, minimizes the potential for selection bias. Randomization was used to minimize bias in the assignment of participants to vaccine groups, to increase the likelihood that known and unknown participant attributes (eg, demographic and baseline characteristics) were evenly balanced across vaccine groups, and to enhance the validity of statistical comparisons across vaccine groups. As stated in the study protocol, available with Dunkle et al. NEJM 2022: "This is an observer-blinded study. To maintain the blind, placebo vaccination via IM route will be included and unblinded site personnel will manage vaccine logistics, preparation, and administration according to the Pharmacy Manual so as to maintain the blind from the remainder of the site personnel and participants. The unblinded site personnel will not be involved in study-related assessments or have participant contact for data collection after administration of trial vaccine. Participants will be randomized according to a list produced by ICON. Prior to production, the randomization specification will be reviewed and agreed by the study team (sponsor and ICON). As block size is considered potentially unblinding information, it will be known to the Study Biostatistician only. An IWRS will be responsible for the allocation of randomization numbers to individual participants. Randomization will take place at baseline after confirmation that the participant continues to meet the inclusion/exclusion criteria. Participants will be randomized in a 2:1 ratio to receive either SARS-CoV-2 rS with Matrix-M1 adjuvant or placebo, administered via IM route. A copy of the randomization code with true treatment allocations will be held by ICON during the study. Another randomization list (containing treatment) will be provided to clinical supplies."
Ethics oversight	The following Institutional Review Boards (IRBs)/Independent Ethics committees reviewed and approved the study: Western Copernicus Group IRB, US; Great Plains IRB, US; Comite de etica en investigacion del Instituto Nacional de Ciencias Medicas y Nutricion, Salvador Zubiran, Mexico; Comite de etica en investigacion de la Unidad de Atencion Medica e Investigacion en Salud S.C., Mexico; Comite de etica en investigacion del Instituto Nacional de Salud Publica, Mexico; Comite de etica en investigacion de Medica Rio Mayo S.C., Mexico; Comite de etica en investigacion del Hospital La Mision S.A. de C.V., Mexico.

Note that full information on the approval of the study protocol must also be provided in the manuscript.

Field-specific reporting

Please select the one below that is the best fit for your research. If you are not sure, read the appropriate sections before making your selection.

☒ Life sciences ☐ Behavioural & social sciences ☐ Ecological, evolutionary & environmental sciences

For a reference copy of the document with all sections, see nature.com/documents/nr-reporting-summary-flat.pdf

Life sciences study design

All studies must disclose on these points even when the disclosure is negative.

Sample size	In general, approximately 25 evaluable (with Day 0 and Day 35 Ab marker data available) vaccine recipient cases are desired to achieve good precision for correlate of risk analyses. For instance, the HVTN 505 trial serves as a precedent where 25 evaluable vaccine recipient cases provided enough data to reasonably characterize correlates of risk for a preventive candidate HIV vaccine (Janes et al., 2017; Fong et al., 2018; Neidich et al., 2019; Gilbert et al., 2020). In addition, simulation studies show that correlates analyses at 20 endpoints have notably lower precision. However, there were only 12 vaccine breakthrough COVID-19 endpoints with D0 and D35 antibody data available for correlates analyses in the PREVENT-19 trial. Therefore, only a subset of the statistical methods were applied. Specifically, the mediation analyses and the multivariable marker correlates of risk analyses were included for COVE and ENSEMBLE and excluded for PREVENT-19.
Data exclusions	As stated in Section 6 of the SAP, "In two-phase sampling data analysis nomenclature, the “phase 1 ptids” are the per-protocol individuals excluding individuals with a COVID failure event or any other evidence of SARS-CoV-2 infection < 7 days post Day 35 visit (the RT-PCR assay is used to define any evidence of SARS-CoV-2 infection). The “phase 2 ptids” are then the subset of these phase 1 ptids in the immunogenicity subcohort with Day 1 and Day 35 Ab marker data available. Thus, marker data for the COVID endpoint cases outside the subcohort will not be used in immunogenicity analyses; these cases are excluded from immunogenicity analyses."<br>For the binding antibody assay: Any plates and samples that did not meet the following acceptance criteria were excluded and repeated:<br>• Plate calibrator curve fit R2 ≥ 0.98<br>• Calibrator replicate signal CV (coefficient of variation) and back-calculated concentration ≤ 20% for standards within LSL (lower sensitivity level) and USL (upper sensitivity level) range<br>• Calibrator replicate signal CV (coefficient of variation) and back-calculated concentration ≤ 25% for LSL and USL standards.<br>• Plate controls signal CV (coefficient of variation) ≤ 20%.<br>• Recoveries of plate controls within +/-30% of the nominal values for MSD-high, MSD-mid, and MSD-low. Recoveries of plate controls within +/-30% of the nominal values for Sercare-high, Seracare-Mid, and In-house prepared serum control. Recoveries of plate controls within +/-25% of the nominal values for Seracare-Negative.<br>• Sample replicate CVs ≤ 20%.
Replication	All of the immune correlates analyses are implemented in automated and reproducible press-button fashion. The analyses code are hosted in a github repo that is open to the public (https://github.com/CoVPN/correlates_reporting2).<br>For the binding antibody assay: Reproducibility was ensured by running 8 controls in all plates assayed including: MSD-high, MSD-mid, MSD-low, Sercare-high, Seracare-Mid, Seracare-Negative, and In-house prepared serum control sample
Randomization	As stated in Dunkle et al. 2022 NEJM, participants "were randomly assigned with the use of a Web-based interactive response system in a 2:1 ratio to receive two 0.5-ml intramuscular injections of either NVX-CoV2373 (5 μg of SARS-CoV-2 recombinant spike protein adjuvanted with 50 μg of Matrix-M) or saline placebo 21 days apart. "
Blinding	The PREVENT-19 trial was an observer-blinded phase 3 efficacy trial. The treatment arm assignment was blinded to the labs running the assays for the correlates analyses.

Reporting for specific materials, systems and methods

We require information from authors about some types of materials, experimental systems and methods used in many studies. Here, indicate whether each material, system or method listed is relevant to your study. If you are not sure if a list item applies to your research, read the appropriate section before selecting a response.

Materials & experimental systems

n/a	Involved in the study
☐	☒ Antibodies
☐	☒ Eukaryotic cell lines
☒	☐ Palaeontology and archaeology
☒	☐ Animals and other organisms
☐	☒ Clinical data
☒	☐ Dual use research of concern

Methods

n/a	Involved in the study
☒	☐ ChIP-seq
☒	☐ Flow cytometry
☒	☐ MRI-based neuroimaging

Antibodies

Antibodies used	MSD SULFO-TAG anti-human IgG antibody 200x. Meso Scale Diagnostics, LLC. Catalog number D21ADF-3. Mouse monoclonal antibody. Diluted 200-fold to prepare 1x solution from stock.
Validation	Certificates of analysis and technical notes are available at https://www.mesoscale.com/en/products/msd-gold-sulfo-tag-nhs-ester-r91ao/

Eukaryotic cell lines

Policy information about cell lines and Sex and Gender in Research

Cell line source(s)	HEK-293 T; source: Master Cell Bank established by Monogram Biosciences circa 1996
Authentication	No formal authentication. Cell line in continuous use since establishment of Master Cell Bank.
Mycoplasma contamination	The HEK-293T cell line tested negative for mycoplasma contamination. Mycoplasma testing is routinely performed per MGRM SOP.
Commonly misidentified lines (See ICLAC register)	None.

Clinical data

Policy information about clinical studies

All manuscripts should comply with the ICMJE guidelines for publication of clinical research and a completed CONSORT checklist must be included with all submissions.

Clinical trial registration: ClinicalTrials.gov number NCT04611802

Study protocol: The protocol is available with the primary publication, Dunkle et al. 2022 NEJM: https://www.nejm.org/doi/full/10.1056/nejmoa2116185. The specific link to the study protocol is: https://www.nejm.org/doi/suppl/10.1056/NEJMoa2116185/suppl_file/nejmoa2116185_protocol.pdf

Data collection:

Data collection is described in the primary publication, Dunkle et al. 2022 NEJM. As stated in Dunkle et al, the PREVENT-19 study was conducted at 113 clinical study sites in the US and 6 in Mexico. Participants received initial injections between December 27, 2020, and February 18, 2021, and were followed through April 19, 2021. The 119 clinical study sites are also listed below:

MEX Cuernavaca Instituto Nacional de Salud Publica (INSP) - Cuernavaca - Centro de Investigacion en Salud P
MEX Guadalajara PanAmerican Clinical Research Mexico S.A de C.V
MEX Merida Unidad de Atencion Medica e Investigacion en Salud (UNAMIS)
MEX Mexico City CAIMED Investigacion en Salud S.A de C.V
MEX Queretaro PanAmerican Clinical Research Mexico
MEX Veracruz FAICIC S. DE R.L. DE C.V.
USA Akron Synexus Clinical Research, US, Inc.
USA Anaheim Anaheim Clinical Trials
USA Anderson Synexus Clinical Research US, Inc.
USA Ann Arbor VA Medical Center
USA Atlanta Atlanta Center for Medical Research
USA Atlanta Morehouse School Of Medicine
USA Atlanta Ponce de Leon Center
USA Atlanta Synexus Clinical Research US, Inc.
USA Aurora University of Colorado Hospital CRS
USA Austin Benchmark Research
USA Baltimore University Of Maryland School Of Medicine
USA Banning Advanced Clinical Research - Rancho Paseo
USA Baton Rouge Meridian Clinical Research, LLC
USA Birmingham Accel Research Sites
USA Bossier City Willis-Knighton Physician Network
USA Boston Beth Israel Deaconess Medical Center
USA Bristol PMG Research of Bristol
USA Chapel Hill University of North Carolina
USA Charleston Medical University of South Carolina, SCTR Research Nexus
USA Charlotte The Charlotte-Mecklenburg Hospital Authority d/b/a Atrium Health
USA Chattanooga WR Clinsearch, LLC
USA Cheney MultiCare Institute for Research & Innovation
USA Chicago Cedar Crosse Research Center
USA Chicago Synexus
USA Chula Vista GW Research Inc
USA Cincinnati Sterling Research Group, Ltd
USA Cincinnati Sterling Research Group, Ltd.
USA Cincinnati Synexus Clinical Research US, Inc.
USA Cleveland Rapid Medical Research, Inc.
USA Colorado Springs Lynn Institute of the Rockies

USA Columbia The Curators of University of Missouri
USA Columbus IACT Health
USA Commack Stony Brook WTC Health Program-Stony Brook Medicine
USA Deland University Clinical Research-Deland, LLC dba Accel Research Sites
USA Detroit Wayne State University/ Children's Hospital of Michigan
USA Durham M3-Emerging Medical Research, LLC
USA Eagle Butte American Indian Clinical Trials Research Network
USA Evansville Synexus USA
USA Evergreen Park Providea Health Partners LLC
USA Fayettville Carolina Institute for Clinical Research
USA Fort Bragg Womack Army Medical Center
USA Fort Worth Benchmark Research
USA Gainesville SIMED Health, LLC / SIMED Research
USA Gulfport MedPharmics, LLC-Biloxi
USA Hallandale Beach M D Clinical
USA Henderson Synexus Clinical Research US, Inc.
USA Hollywood Research Centers of America
USA Houston Baylor College of Medicine
USA Houston Texas Center for Drug Development, Inc
USA Iowa City University of Iowa Medical Center
USA Jacksonville Jacksonville Center for Clinical Research
USA Knoxville PMG Research, Inc. d/b/a PMG Research of Knoxville
USA La Mesa Velocity Clinical Research
USA Lakeland Meridien Research/Accel Research
USA Las Vegas Clinical Research Consortium
USA Lenexa Johnson County Clin-Trials, Inc.
USA Little Rock Lynn Institute of the Ozarks
USA Little Rock Preferred Research Partners, Inc.
USA Los Alamitos WR-PRI, LLC
USA Los Angeles National Research Institute
USA McAllen Centex Studies, Inc
USA Medford CRISOR, LLC - Clinical Research Institute of Southern Oregon, PC
USA Memphis Clinical Neurosciecne Solutions, Inc. dba CNS Healthcare
USA Meridian Advanced Clinical Research
USA Metairie Med Pharmics, LLC
USA Miami Miami Veterans Affairs Medical Center
USA Miami Suncoast Research Associates, LLC
USA Miami Suncoast Research Group, LLC
USA Minneapolis University of Minnesota
USA Nashville Meharry Medical College
USA New York Weill Cornell Chelsea CRS
USA Newport News Health Research of Hampton Roads, Inc
USA Norfolk Meridian Clinical Research
USA North Hollywood Transitional Research Group, Inc.
USA Oklahoma City Lynn Health Science Institute
USA Oklahoma City Medical Research International
USA Omaha Meridian Clinical Research Associates, LLC
USA Omaha University Of Nebraska Medical Center
USA Orlando Clinical Neuroscience Solutions Inc
USA Orlando Headlands Research Orlando
USA Phoenix HOPE Research Institute
USA Pinellas Park Synexus Clinical Research US, Inc
USA Plano Research Your Health
USA Pomona Empire Clinical Research
USA Ponce Ponce School Of Medicine
USA Providence The Miriam Hospital
USA Raleigh M3 Wake Research, Inc
USA Richfield Synexus Clinical Research US, Inc.
USA Rochester Rochester Clinical Research, Inc.
USA Rocky Mount PMG Research of Rocky Mount, LLC
USA Sacramento Benchmark Research
USA Sacramento University of California Davis Medical Center
USA San Antonio Synexus, US - San Antonio
USA San Antonio University of Texas Health Science Center San Antonio
USA San Diego California Research Foundation
USA San Juan Universidad de Puerto Rico - Recinto de Ciencias Medicas (UPR-MSC) - Centro Mujer y Salud (C
USA Seattle University of Washington VTEU - Seattle and King County Public Health STD clinic
USA Sioux City Meridian Clinical Research
USA St. Louis Sundance Clinical Research, LLC
USA Stockbridge Clinical Research Atlanta
USA Tampa Jedidiah Clinical Research
USA Tampa University of South Florida
USA Tempe AMR Tempe Clinical Research Consortium
USA The Villages Synexus Clinical Research US, Inc.
USA Tomball DM Clinical Research
USA Valparaiso Buynak Clinical Research, P.C.

USA Vista Synexus Clinical Research US, Inc.
USA Warwick Velocity Clinical Research, Providence
USA Washington Howard University Hospital Howard/ University College of Medicine
USA West Jordan Advanced Clinical Research
USA West Palm Beach Comprehensive Clinical Trials, Llc
USA Wilmington PMG Research of Wilmington, LLC
USA Woodland Woodland Clinic Medical Group

Outcomes

The primary, secondary, and exploratory objectives and endpoints of the PREVENT-19 trial are all described in the Supplementary Appendix of the primary publication, Dunkle et al. 2022 NEJM. The specific link to the Appendix is: https://www.nejm.org/doi/suppl/10.1056/NEJMoa2116185/suppl_file/nejmoa2116185_appendix.pdf. The primary and secondary objectives, along with their methods of assessment, are also listed below:
Primary objective: To evaluate the efficacy of a two-dose regimen of SARS-CoV-2 rS adjuvanted with Matrix-M™ compared to placebo against RT-PCR-confirmed symptomatic Covid-19 illness diagnosed ≥7 days after completion of the second injection in the initial set of vaccinations of adult participants ≥18 years of age.
Primary end point: • First episode of RT-PCR-positive mild, moderate, or severe Covid-19, where severity is defined as:
Mild Covid-19 (≥1 of the following):
• Fever (defined by subjective or objective measure, regardless of use of anti-pyretic medications)
• New onset cough
• ≥2 additional Covid-19 symptoms:

o New onset or worsening of shortness of breath or difficulty breathing compared to baseline.
o New onset fatigue.
o New onset generalized muscle or body aches.
o New onset headache.
o New loss of taste or smell.
o Acute onset of sore throat, congestion, or runny nose.
o New onset nausea, vomiting, or diarrhea.
OR Moderate Covid-19 (≥1 of the following):
• High fever (≥38.4°C) for ≥3 days (regardless of use of anti-pyretic medications, need not be contiguous days).
• Any evidence of significant LRTI:
o Shortness of breath (or breathlessness or difficulty breathing) with or without exertion (greater than baseline).
o Tachypnea: 24 to 29 breaths per minute at rest.
o SpO_2: 94% to 95% on room air.
o Abnormal chest X-ray or chest CT consistent with pneumonia or LRTI.
• Adventitious sounds on lung auscultation
(e.g., crackles/rales, wheeze, rhonchi, pleural rub, stridor).
OR Severe Covid-19 (≥1 of the following):
• Tachypnea: ≥30 breaths per minute at rest.
• Resting heart rate ≥125 beats per minute.
• SpO_2: ≤93% on room air or PaO_2/FiO_2 <300 mmHg.
• High flow O_2 therapy or NIV/NIPPV (e.g., CPAP or BiPAP).
• Mechanical ventilation or ECMO.
• One or more major organ system dysfunction or failure to be defined by diagnostic testing/clinical syndrome/interventions, including any of the following:
o Acute respiratory failure, including ARDS.
o Acute renal failure.
o Acute hepatic failure.
o Acute right or left heart failure.
o Septic or cardiogenic shock (with shock defined as SBP <90 mm Hg OR DBP <60 mm Hg).
o Acute stroke (ischemic or hemorrhagic).
o Acute thrombotic event: AMI, DVT, PE.
o Requirement for: vasopressors, systemic corticosteroids, or hemodialysis.
• Admission to an ICU.
Death.

Key Secondary Objective:
• To evaluate the efficacy of a two-dose regimen of SARS-CoV-2 rS adjuvanted with Matrix-M™ compared to placebo against RT-PCR-confirmed symptomatic Covid-19 illness due to a SARS-CoV-2 variant not considered as a “variant of concern / interest” according to the CDC Variants Classification, diagnosed ≥7 days after completion of
the second injection in the initial set of vaccinations of adult participants ≥18 years of age.
Key secondary endpoint:
• First episode of RT-PCR-positive Covid-19, as defined under the primary end point, shown by gene sequencing to represent a variant not considered as a “variant of concern / interest” according to the CDC Variants Classification.
