# Supplementary material for: Immune correlates analysis of the PREVENT-19 COVID-19 vaccine efficacy clinical trial
Source: Nat Commun. 2023 Jan 19;14:331. doi: 10.1038/s41467-022-35768-3 (PMC9851580; doi:10.1038/s41467-022-35768-3)
Supplement: Supplementary file 2 — Peer review file [file 41467_2022_35768_MOESM2_ESM.pdf]

nature portfolio

Peer Review File

Immune Correlates Analysis of the PREVENT-19 COVID-19 Vaccine Efficacy Clinical Trial

Open Access This file is licensed under a Creative Commons Attribution 4.0 International License, which permits use, sharing, adaptation, distribution and reproduction in any medium or format, as long as you give appropriate credit to the original author(s) and the source, provide a link to the Creative Commons license, and indicate if changes were made. In the cases where the authors are anonymous, such as is the case for the reports of anonymous peer reviewers, author attribution should be to 'Anonymous Referee' followed by a clear attribution to the source work. The images or other third party material in this file are included in the article's Creative Commons license, unless indicated otherwise in a credit line to the material. If material is not included in the article's Creative Commons license and your intended use is not permitted by statutory regulation or exceeds the permitted use, you will need to obtain permission directly from the copyright holder. To view a copy of this license, visit http://creativecommons.org/licenses/by/4.0/.

REVIEWER COMMENTS

Reviewer #1 (Remarks to the Author):

This study assessed the correlates of protection for a licensed recombinant spike protein vaccine, using data from 12 breakthrough cases and sampled non-cases from a phase 3 trial in the US. Using a case-cohort study design and established statistical approaches, the authors reported correlates of risk and correlates of protection by ranges of anti-spike IgG and neutralizing antibody responses. Estimates were compared with correlates estimates of covid vaccines by other platforms using the same study design and statistical methods. This study adds value to pre-existing correlates estimates, and supports the potential of vaccine approval across platforms. The paper is in general well-written. It would be helpful to clarify a few points as below (and apologies if I missed anything).

1. Page 3. Abstract. “However, the relatively few breakthrough cases in PREVENT-19 limited the ability to infer a stronger correlate.” Could the authors clarify if here “stronger” correlate means higher statistical power?

2. Page 6. Introduction. “it was only possible to apply a subset of the correlates statistical methods specified in the harmonized Statistical Analysis Plan”. It seems mediation analysis has not been done due to small numbers of breakthrough cases, and it might help to summarize which part has been done/not done.

3.Page 13. Results. “COV002 in the United Kingdom (two doses of AstraZeneca AZD1222/ChAdOx1 nCoV-19 at D0 and D28)” As far as I know, COV002 in the UK is a phase 2/3 trials of varied intervals between 2 doses ranging from 28 days to longer than 9 months (see reference 29). Did the correlates for COV002 re-analyzed here restricting to vaccines schedule of D0-D28 only?

4. Page 21. Methods. I didn’t find a definition of primary endpoint, and thus would suggest add the definition including what symptoms are eligible for testing etc.

5. Page 23. Covariate adjustment. Providing relevant references on ensemble learning would be helpful, and also how these input variables were measured, e.g continuous/categorical variable, and the specific categories if applicable.

Reviewer #2 (Remarks to the Author):

This work shows the analysis of IgG and nAb as potential surrogate endpoints of vaccine efficacy of a recombinant spike protein nanoparticle vaccine (the NVX-CoV2373) from the analysis of the PREVENT-19 trial and then in comparison with other vaccines. Therefore, it provides very important results for future development of COVID19 vaccines.

A big force of this work is its integration on a very straightforward program for the analysis of COVID19 surrogate endpoint led by Dr Gilbert. The SAP has been publicly available a long time ago before several trials data have been analyzed. The code and data are all available. The quality and precision of the information provided is outstanding.

The weakness of the present paper, clearly recognized by the authors, is the very low number of endpoints (N=12) used in the analysis leading to an important uncertainty of the statistical estimations and the impossibility to perform proper stratified analysis (e.g. by age, see sup fig 2). I fully agree with the authors recognizing the importance of the signal in regards of the low number of events. However, it sounds to me that the conclusions drawn based on the results given in figure 4 C, D, E, F are too far from the data (ie line 275 "these results suggest that …. High spike IgG appears to be a better marker…") because the CI are very wide due to the restricted number of events when vaccine recipients were divided into subgroups defined by marker level above a specific threshold. In other words, the hypothesis that the risk decreases incrementally as antibody level increase like in COVE cannot be excluded. In the same spirit, I am not comfortable with the lines 378-381 in the discussion, whereas the follow-up about the precision of spike IgG is fine.

It sounds to me that the discussion could be clarified between the statistical surrogate and the mechanistic surrogate. The role of binding IgG as statistical surrogate, reinforced by the present analysis, is making a lot of sense. Binding IgG may capture also other immune responses, correlated to this marker, that are protecting against the infection. And this surrogacy may still be relevant with other variants such as delta and omicron. It is different for neutralization which is more specific to the variant considered. It may be interested to evaluate the joint contribution of the two markers in same model unless their correlation precludes the identifiability of each effect in the same model.

The limitation due to the short follow-up could be mitigated. Actually, in my point of view, the work done with such surrogate analysis is to estimate the relationship (that could be causal) between the surrogate marker and the efficacy of the vaccine as measured in the trial (without long follow-up). This is done here. The interest of a longer follow-up would be to confirm that while the Ab concentration is decreasing, the risk of symptomatic infection is increasing. Obviously, the relationship can change over time (due to an increasing role of the T cell response for instance). Therefore, it is wise to recommend analyses with longer follow-up but should it be really necessary to confirm that we get good statistical surrogate endpoint for early endpoint?

Minor comments:

Methods: covariate adjustment: add the information about the statistical model used in addition with the Ensemble learning approach.

Line 359: delete “RNA”

Fig 3B: add in the legend that vaccine Med and vaccine High are confounded (vaccine High is indeed not visible)

P33 SAP: a reference is missing after Hubbard 2016

The supplementary ref 3 (arxiv) could be updated by the Biostatistics ref.

Reviewer #3 (Remarks to the Author):

This is a very important, well designed and methodologically very solid study to analyze the role of Bau and nAb as correlates of protection against PCR confirmed and symptomatic breakthrough infection.

The study evaluates the protection associated with different levels of Bau and nAb, confirming their role of CoP (albeit recognizing that this is capturing most but not all of the protection). The study first analyses a large phase 3 data of NVX-CoV2373, and the methodology is then applied to 2 other phase 3 trials involving different vaccine classes (nRNA1273 and Ad26.CoV2.S). Although some differences exist across vaccines (in particular for binding IgG), the cutoffs associated with >90% protection are largely consistent.

There are inherent limitations to this study, which are recognized and discussed by the authors. In particular the small number of breakthrough and the absence of omicron variants, as well as the absence of severe covid-19 cases limit the precision of the predictions, as well as their use to guide public health decision. In addition the study is limited to SARS-CoV-2 naïve patients, and cannot consider the protection conferred by vaccination and infection (which is becoming the majority of cases nowadays in Western world). Nonetheless, this does not hamper the importance of these findings, which will stimulate research on IgG and nAb as correlates of protection against symptomatic Covid-19 infection.

I have only minor comments on the form:

• The differences found in HR associated with IgG protection between the different vaccines are surprising (Table 3). These differences are recognized by the authors, and attributed to parameter uncertainty and wide confidence interval, as well as difference in the horizon time used for infection. However, the same argument apply to nAb, for which results are nonetheless aligned. Despite these results the authors seem to value IgG (at least for 2373) in the discussion (“spike IgG may be a more discriminating correlate”). Please address and perhaps tone down these statements.

• It is not clear how the cutoff values for efficacy have been selected. In my opinion, it would be easier to communicate on the values associated with a given level of VE, say 80, 90 and 95%, which could be used as pharmacodynamic targets, instead of giving the levels of protection associated with some quantiles of the IgG/nAb observed distribution.

• Given the quality of the study, I think that some efforts could be done to improve the readability of the figures. Sometimes there is an inner grid, sometimes not ; sometimes this x-axis has “threshold” sometimes not (while in my understanding could be the same) ; Fig 4E,F are difficult to read, same for most labels in inner graphs. Be consistent in axis for fig5 and 6 : “controlled VE” and “Controlled VE after 59 days” while this is the same results for 2373, LLOQ in xaxis but 0.1 and 1 in the other figure. Be consistent in axis

REVIEWER COMMENTS

Reviewer #1 (Remarks to the Author):

This study assessed the correlates of protection for a licensed recombinant spike protein vaccine, using data from 12 breakthrough cases and sampled non-cases from a phase 3 trial in the US. Using a case-cohort study design and established statistical approaches, the authors reported correlates of risk and correlates of protection by ranges of anti-spike IgG and neutralizing antibody responses. Estimates were compared with correlates estimates of covid vaccines by other platforms using the same study design and statistical methods. This study adds value to pre-existing correlates estimates, and supports the potential of vaccine approval across platforms. The paper is in general well-written. It would be helpful to clarify a few points as below (and apologies if I missed anything).
1. Page 3. Abstract. “However, the relatively few breakthrough cases in PREVENT-19 limited the ability to infer a stronger correlate.” Could the authors clarify if here “stronger” correlate means higher statistical power?

Response: In the need to adhere to the 150-word limit for the Abstract, this sentence had to be deleted. However, we have added the following sentence to the Discussion: “Another limitation is that the relatively few breakthrough cases in PREVENT-19 limited precision in the estimates of how vaccine efficacy changes with the correlate.”

2. Page 6. Introduction. “it was only possible to apply a subset of the correlates statistical methods specified in the harmonized Statistical Analysis Plan”. It seems mediation analysis has not been done due to small numbers of breakthrough cases, and it might help to summarize which part has been done/not done.

Response: We added the sentence: “Specifically, the mediation analyses and the multivariable marker correlates of risk analyses were included for COVE and ENSEMBLE and excluded for PREVENT-19.”

3.Page 13. Results. “COV002 in the United Kingdom (two doses of AstraZeneca AZD1222/ChAdOx1 nCoV-19 at D0 and D28)” As far as I know, COV002 in the UK is a phase 2/3 trials of varied intervals between 2 doses ranging from 28 days to longer than 9 months (see reference 29). Did the correlates for COV002 re-analyzed here restricting to vaccines schedule of D0-D28 only?

Response: We appreciate that it is necessary to describe the cohort of vaccine recipients that was included in the analysis. Our objective was to provide the identical results as in the Feng et al. (2021, *Nature Medicine*) paper, and their analysis included all per-protocol two-dose recipients, aggregating over the different dose intervals. We added the sentence: “COV002 included variable time intervals between the first and second doses of vaccine; to replicate the COV002-specific correlates analysis of Feng et al.[26] we present the results including all dose intervals.”

4. Page 21. Methods. I didn't find a definition of primary endpoint, and thus would suggest add the definition including what symptoms are eligible for testing etc.

Response: We concur that complete clarity is needed on the symptoms defining the endpoint for which correlates were studied. The Dunkle et al. (2021, *NEJM*) article provides the definition, the same definition that we used. Therefore it seems unnecessary to repeat the list provided in Dunkle et al., and instead we clarified the language pointing to the fact that we used the same endpoint as used in Dunkle et al.: "D35 antibody measurements were evaluated as correlates for the same primary virologically-confirmed symptomatic COVID-19 endpoint that was studied in the primary efficacy analysis[6], with the same set of symptoms defining the endpoint as listed in Dunkle et al.[6]"

5. Page 23. Covariate adjustment. Providing relevant references on ensemble learning would be helpful, and also how these input variables were measured, e.g continuous/categorical variable, and the specific categories if applicable.

Response: We have added Supplementary Table 6, which includes the definitions of the individual baseline variables used in the ensemble machine learning. We have also added details of the ensemble learning approach, along with a reference (van der Laan, Polley, Hubbard; *Stat Appl Genet Mol Biol* 2007), in the "Covariate adjustment" subsection of the Methods section.

Reviewer #2 (Remarks to the Author):

This work shows the analysis of IgG and nAb as potential surrogate endpoints of vaccine efficacy of a recombinant spike protein nanoparticle vaccine (the NVX-CoV2373) from the analysis of the PREVENT-19 trial and then in comparison with other vaccines. Therefore, it provides very important results for future development of COVID19 vaccines.

A big force of this work is its integration on a very straightforward program for the analysis of COVID19 surrogate endpoint led by Dr Gilbert. The SAP has been publicly available a long time ago before several trials data have been analyzed. The code and data are all available. The quality and precision of the information provided is outstanding.

The weakness of the present paper, clearly recognized by the authors, is the very low number of endpoints (N=12) used in the analysis leading to an important uncertainty of the statistical estimations and the impossibility to perform proper stratified analysis (e.g. by age, see sup fig 2). I fully agree with the authors recognizing the importance of the signal in regards of the low number of events. However, it sounds to me that the conclusions drawn based on the results given in figure 4 C, D, E, F are too far from the data (ie line 275 "these results suggest that …. High spike IgG appears to be a better marker…") because the CI are very wide due to the

restricted number of events when vaccine recipients were divided into subgroups defined by marker level above a specific threshold. In other words, the hypothesis that the risk decreases incrementally as antibody level increase like in COVE cannot be excluded. In the same spirit, I am not comfortable with the lines 378-381 in the discussion, whereas the follow-up about the precision of spike IgG is fine.

Response: Thank you for the complimentary comments. We agree with the comments about how some statements suggest results that do not have enough statistical precision to warrant the statements. Accordingly, we deleted the sentence "These results suggest that for NVX-CoV2373, high spike IgG appears to be a better marker of very low risk than high nAb ID50." We also revised (as underlined) the later sentence: "The apparently stronger correlate of protection for NVX-CoV2373 than for mRNA-1273 is indicated by the steeper estimated vaccine efficacy curve, although the true vaccine efficacy curves could readily be the same as there is inadequate statistical precision to determine whether the curves differ."
Regarding the comment about lines 378-381 in the Discussion, we have made the following revision (underlined):
"Along the same vein, the level of precision of the data limits the ability to uncover differences in the performance of the three antibody markers, and in terms of point estimates the results are similar, with possible exceptions of more steeply decreasing risk with threshold of spike IgG and with threshold of RBD IgG than with threshold of nAb ID50 in the nonparametric threshold analysis (Figure 4 Panels D and E vs. Panel F)."

It sounds to me that the discussion could be clarified between the statistical surrogate and the mechanistic surrogate. The role of binding IgG as statistical surrogate, reinforced by the present analysis, is making a lot of sense. Binding IgG may capture also other immune responses, correlated to this marker, that are protecting against the infection. And this surrogacy may still be relevant with other variants such as delta and omicron. It is different for neutralization which is more specific to the variant considered. It may be interested to evaluate the joint contribution of the two markers in same model unless their correlation precludes the identifiability of each effect in the same model.

Response: These are interesting points, and we agree that the neutralization marker should be closer to a mechanistic correlate of protection than binding IgG. We have some reticence to add discussion on this point given the Discussion is already fairly long. In addition, while it would be interesting to study binding IgG and nAb ID50 in the same model of the COVID-19 outcome, the markers are positively correlated (Spearman rank 0.80, which is reported), and moreover we would like to not add post-hoc analyses.

The limitation due to the short follow-up could be mitigated. Actually, in my point of view, the work done with such surrogate analysis is to estimate the relationship (that could be causal) between the surrogate marker and the efficacy of the vaccine as measured in the trial (without long follow-up). This is done here. The interest of a longer follow-up would be to confirm that while the Ab concentration is decreasing, the risk of symptomatic infection is increasing.

Obviously, the relationship can change over time (due to an increasing role of the T cell response for instance). Therefore, it is wise to recommend analyses with longer follow-up but should it be really necessary to confirm that we get good statistical surrogate endpoint for early endpoint?

Response: We take your point, that this work does define a surrogate endpoint for short follow-up, which is a distinct question from studying a surrogate endpoint for longer follow-up. Therefore the longer-follow-up analysis does not need to be thought of as supporting or refuting the results on short follow-up; instead it answers a different question. We revised the discussion to enhance clarity.

Minor comments:

Methods: covariate adjustment: add the information about the statistical model used in addition with the Ensemble learning approach.

Response: We have added Supplementary Table 7, which provides information on the ensemble Superlearner classification model, including the individual learners sorted by weight and the predictors within each learner. We have also added the following details of the Ensemble learning approach in the "Covariate adjustment" subsection of the Methods section:

"Briefly, the ensemble Superlearner classification model (Supplementary Table 7) was developed using the binary outcome of COVID-19 endpoint occurrence post-enrollment and limiting to no more than 20 input variables. Each quantitative and ordinal variable was pre-scaled to have empirical mean = 0 and standard deviation = 1. The ensemble model was trained using placebo arm data. CV-predictions were made on placebo arm data, while predictions were made on vaccine arm data. Superlearner modeling was conducted using the negative log-likelihood loss function and results were averaged over 10 random seeds. A library of adaptive and non-adaptive learners/classifiers from the SuperLearner R package was used with and without screens (Supplementary Table 8). Two levels of cross-validation were used: 5-fold outer level to compute CV-AUC, and 5-fold inner level to estimate ensemble weights. Classification accuracy of the ensemble model (Superlearner), discrete Superlearner and top two best-performing individual learners were presented as ROC curves (CV-AUC for placebo arm, AUC for vaccine arm) (Supplementary Figure 5). CV-AUC[40,41] was estimated using the R package vimp available on CRAN."

Line 359: delete "RNA"

Response: Deleted the typo.

Fig 3B: add in the legend that vaccine Med and vaccine High are confounded (vaccine High is indeed not visible)

Response: Added a clarifying note to the legend of Fig 3B.

P33 SAP: a reference is missing after Hubbard 2016

Response: Added back the reference after Hubbard 2016.

The supplementary ref 3 (arxiv) could be updated by the Biostatistics ref.

Response: Updated, thank you for the catch.

Reviewer #3 (Remarks to the Author):

This is a very important, well designed and methodologically very solid study to analyze the role of Bau and nAb as correlates of protection against PCR confirmed and symptomatic breakthrough infection.
The study evaluates the protection associated with different levels of Bau and nAb, confirming their role of CoP (albeit recognizing that this is capturing most but not all of the protection). The study first analyses a large phase 3 data of NVX-CoV2373, and the methodology is then applied to 2 other phase 3 trials involving different vaccine classes (nRNA1273 and Ad26.CoV2.S). Although some differences exist across vaccines (in particular for binding IgG), the cutoffs associated with >90% protection are largely consistent.
There are inherent limitations to this study, which are recognized and discussed by the authors. In particular the small number of breakthrough and the absence of omicron variants, as well as the absence of severe covid-19 cases limit the precision of the predictions, as well as their use to guide public health decision. In addition the study is limited to SARS-CoV-2 naïve patients, and cannot consider the protection conferred by vaccination and infection (which is becoming the majority of cases nowadays in Western world). Nonetheless, this does not hamper the importance of these findings, which will stimulate research on IgG and nAb as correlates of protection against symptomatic Covid-19 infection.
I have only minor comments on the form:
• The differences found in HR associated with IgG protection between the different vaccines are surprising (Table 3). These differences are recognized by the authors, and attributed to parameter uncertainty and wide confidence interval, as well as difference in the horizon time used for infection. However, the same argument apply to nAb, for which results are nonetheless aligned. Despite these results the authors seem to value IgG (at least for 2373) in the discussion ("spike IgG may be a more discriminating correlate"). Please address and perhaps tone down these statements.

Response: Agree it is helpful to tone down that statement; revised it to: "Along the same vein, the level of precision of the data limits the ability to uncover differences in the

performance of the three antibody markers, and in terms of point estimates the results are similar, with possible exceptions of more steeply decreasing risk with threshold of spike IgG and with threshold of RBD IgG than with threshold of nAb ID50 in the nonparametric threshold analysis (Figure 4 Panels D and E vs. Panel F).”

• It is not clear how the cutoff values for efficacy have been selected. In my opinion, it would be easier to communicate on the values associated with a given level of VE, say 80, 90 and 95%, which could be used as pharmacodynamic targets, instead of giving the levels of protection associated with some quantiles of the IgG/nAb observed distribution.

Response: Agreed that is useful, and we added the following sentence to the results in section *For all three markers, vaccine efficacy increases with D35 level* : “Additionally, the D35 marker values associated with 80% and 90% VE were: spike IgG, 340 BAU/ml (<LLOQ=1.35, 1287) and 1577 BAU/ml (26, >ULOQ=6934), respectively; RBD IgG, 456 BAU/ml (<LLOQ=30.6, 1595) and 2185 BAU/ml (100, >ULOQ=9801), respectively; and nAb ID50, 81 IU50/ml (<LOD=2.612, 369) and 444 IU50/ml (<LOD=2.612, >7230), respectively.”

• Given the quality of the study, I think that some efforts could be done to improve the readability of the figures. Sometimes there is an inner grid, sometimes not ; sometimes this x-axis has “threshold” sometimes not (while in my understanding could be the same) ; Fig 4E,F are difficult to read, same for most labels in inner graphs. Be consistent in axis for fig5 and 6 : “controlled VE” and “Controlled VE after 59 days” while this is the same results for 2373, LLOQ in xaxis but 0.1 and 1 in the other figure. Be consistent in axis

Response: We have made the following revisions to improve the readability/consistency of the figures:

- Removed any inner grids, so that no figure panels have an inner grid
- Increased font size on x- and y-axis labels
- On Fig. 4G-I: We have removed the log10 values of threshold (as this information is redundant to include) and revised the layout to improve readability, including removing scientific notation, combining columns, and removing redundant rows
- On Fig. 6: We have relabeled the x-axis tick marks to include “LLOQ” and “LOD” in panels A and B, respectively, for consistency with Figs. 4 and 5.

Regarding “Be consistent in axis for fig5 and 6 : “controlled VE” and “Controlled VE after 59 days” while this is the same results for 2373”: All panels that exclusively present PREVENT-19 data do have harmonized y-axis notation (Fig. 4A-4F, Fig. 5). However, Fig. 6 presents data from four different phase 3 COVID-19 vaccine efficacy trials, and the follow-up periods for VE assessment differed. As stated in the legend of Fig. 6: “The follow-up periods for the VE assessment were: COVE (doses D1, D29), 7 to 100 days post D57; ENSEMBLE-US (one dose, D1), 1 to 53 days post D29; PREVENT-19 (doses D0, D21), 7 to 59 days post D35; COV002 (doses D0, D28; 28 days post D28 until the end of the study period).” As this information would

be too complicated to include on the y-axis, we have opted to keep the y-axis label concise and keep this information in the figure legend.

Regarding, “threshold”, the x-axis labels of Fig. 4D-F are correct as is. Panels A through C present the probability of COVID-19 by 59 days post Day 35 when the antibody marker is *at* the given x-value. In contrast, panels D through F present the probability of COVID-19 by 59 days post Day 35 when the antibody marker has a value *above* that of the given x-value. For clarity, we have made the following revisions:

“When vaccine recipients were divided into subgroups defined by their D35 antibody marker level *above a specific threshold* (vs. at a given value, as in Figure 4A-C) and varying the threshold over the range of values…” (first paragraph of Results subsection “For all three markers, D35 level is inversely correlated with risk of COVID-19 in vaccine recipients”).

** See Nature Portfolio’s author and referees' website at www.nature.com/authors for information about policies, services and author benefits.
